# Colorful seashells: Identification of haem pathway genes associated with the synthesis of porphyrin shell color in marine snails

**DOI:** 10.1002/ece3.3552

**Published:** 2017-10-30

**Authors:** Suzanne T. Williams, Anne E. Lockyer, Patricia Dyal, Tomoyuki Nakano, Celia K. C. Churchill, Daniel I. Speiser

**Affiliations:** ^1^ Department of Life Sciences Natural History Museum London UK; ^2^ Institute of Environment, Health and Societies Brunel University London Uxbridge UK; ^3^ Core Research Laboratories Natural History Museum London UK; ^4^ Seto Marine Biological Laboratory Kyoto University Nishimuro Wakayama Prefecture Japan; ^5^ Marine Science Institute University of California Santa Barbara CA USA; ^6^ Department of Biological Sciences University of South Carolina Columbia SC USA

**Keywords:** color, heme, mollusk, pigment, porphyrin, shell, uroporphyrin

## Abstract

Very little is known about the evolution of molluskan shell pigments, although Mollusca is a highly diverse, species rich, and ecologically important group of animals comprised of many brightly colored taxa. The marine snail genus *Clanculus* was chosen as an exceptional model for studying the evolution of shell color, first, because in *Clanculus margaritarius* and *Clanculus pharaonius* both shell and foot share similar colors and patterns; and second, because recent studies have identified the pigments, trochopuniceus (pink‐red), and trochoxouthos (yellow‐brown), both comprised of uroporphyrin I and uroporphyrin III, in both shell and colored foot tissue of these species. These unusual characteristics provide a rare opportunity to identify the genes involved in color production because, as the same pigments occur in the shell and colored foot tissue, the same color‐related genes may be simultaneously expressed in both mantle (which produces the shell) and foot tissue. In this study, the transcriptomes of these two *Clanculus* species along with a third species, *Calliostoma zizyphinum*, were sequenced to identify genes associated with the synthesis of porphyrins. *Calliostoma zizyphinum* was selected as a negative control as trochopuniceus and trochoxouthos were not found to occur in this species. As expected, genes necessary for the production of uroporphyrin I and III were found in all three species, but gene expression levels were consistent with synthesis of uroporphyrins in mantle and colored foot tissue only in *Clanculus*. These results are relevant not only to understanding the evolution of shell pigmentation in *Clanculus* but also to understanding the evolution of color in other species with uroporphyrin pigmentation, including (mainly marine) mollusks soft tissues and shells, annelid and platyhelminth worms, and some bird feathers.

## 1 BACKGROUND

Color and pattern are important features of morphological adaptive variation and are often associated with crypsis, aposematism, and mating displays (Ruxton, Sherratt, & Speed, 2004). Significant advances have been made toward the characterization of pigments and their biosynthetic pathways for plants, vertebrates, and certain invertebrate groups (e.g., Braasch, Schartl, & Volff, 2007; Grotewold, 2006; Hodges & Derieg, 2009; Joron et al., 2006; Nijhout, 2010; Wittkopp & Beldade, 2009; Wittkopp, Williams, Selegue, & Carroll, 2003); however, the molecular pathways leading to shell pigmentation have not been completely elucidated for any mollusk (Mann & Jackson, 2014). The phylum Mollusca is highly diverse, species rich, ecologically important, and abounding in colorful exemplars, so our lack of understanding about pigment evolution in this clade is a serious gap in our knowledge of how color has evolved in the natural world (Williams, 2017).

Most molluskan groups are immediately recognizable by their calcareous shells, many of which are strongly pigmented. Mollusk shells are secreted by the outer fold of the mantle, and both pigmentation and shell growth are under neurosecretory control (Boettiger, Ermentrout, & Oster, 2009; Budd, McDougall, Green, & Degnan, 2014). Although shell color can be affected by food intake, and in some species may depend entirely on diet (e.g., Ino, 1949, 1958; Leighton, 1961), breeding studies in both bivalves and gastropods have shown that color variation in many species is a heritable trait, and in some cases, inheritance patterns can be explained by variation at a single locus (e.g., Kobayashi, Kawahara, Hasakura, & Kijima, 2004; Liu, Wu, Zhao, Zhang, & Guo, 2009).

The molecular processes involved in the synthesis of shell color have been studied in some detail in only a few mollusks. Generally, a suite of potential genes have been identified that may have some control over shell pigmentation, although in most studies it is not possible to rule out that some of these genes may rather be involved in biomineralization (e.g., Bai, Zheng, Lin, Wang, & Li, 2013; Guan, Huang, & He, 2011; Lemer, Saulnier, Gueguen, & Planes, 2015; Qin, Liu, Zhang, Zhang, & Guo, 2007; Richards et al., 2013; Yuan, He, & Huang, 2012; Zou et al., 2014). A study on *Haliotis asinina* (abalone) showed that more than one‐quarter of the genes expressed in the mantle encode secreted proteins, indicating that hundreds of proteins may be contributing to shell construction (Jackson et al., 2006). Only one of these genes was found to map precisely to gastropod shell pigmentation patterns (Jackson, Worheide, & Degnan, 2007; Jackson et al., 2006), although the pigment is unknown. Despite in‐depth molecular investigations trying to determine the genes involved in color production, to date, no study has been able to completely elucidate the molecular pathway used in shell pigmentation for mollusks (Mann & Jackson, 2014).

**Figure 1:**
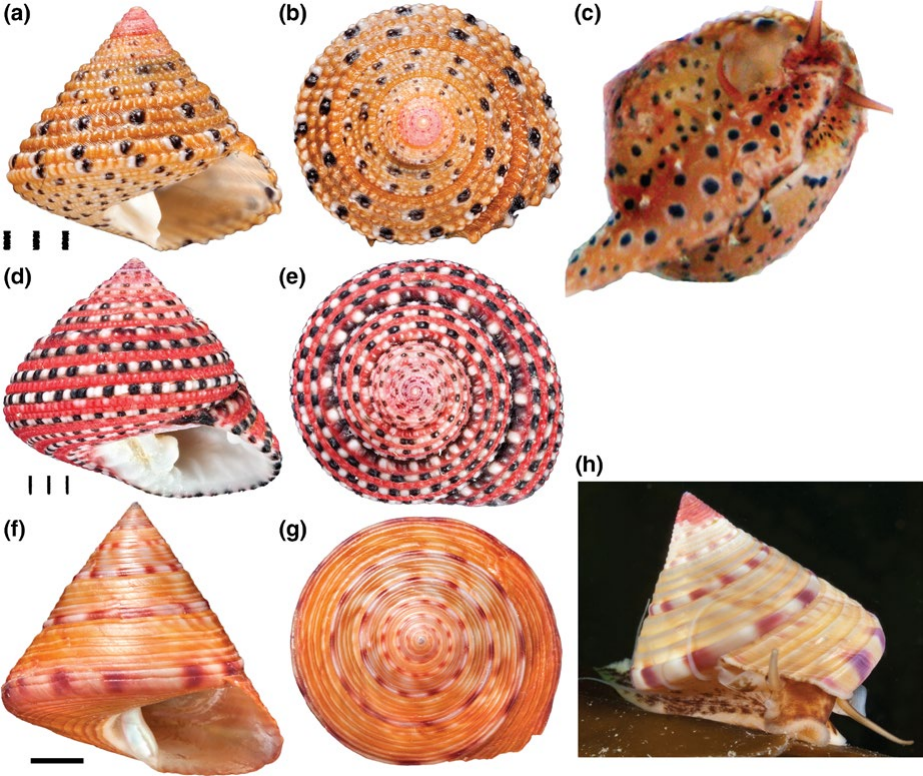
Photographs of species used in this study. (a–c) *Clanculus margaritarius* C. (a, b) Two views of a shell of *Clanculus margaritarius* C (specimen #2). Note that this specimen is subadult. (c) Colored foot of a live animal. Note that the color and pattern are the same as found on the shell. (d, e) Two views of a *Clanculus pharaonius* shell (specimen #4). (f–h) *Calliostoma zizyphinum*. (f, g) Two views of a shell of *Calliostoma zizyphinum* (specimen #2). (h) Living animal showing foot color (not the same specimen). Note that the foot color and pattern in this species do not match the shell. Scale bars for *Clanculus* spp are in mm. Scale bar for *Calliostoma* is 1 cm

The vetigastropod genus, *Clanculus*, and in particular the species *C. margaritarius* and *C. pharaonius* (Trochidae, Trochoidea)*,* are suitable models for studying the synthesis and evolution of molluskan shell color (Figure 1) because their shell pigments are known. A recent study used a combination of biochemical and multimodal spectroscopic methods to identify pigments responsible for three predominant shell colors in these species (Williams et al., 2016). Two pigments, trochopuniceus and trochoxouthos, are responsible for the dominant colors of pink‐red and yellow‐brown, respectively, and traces of eumelanin are likely responsible for black spots on the shells. Trochopuniceus and trochoxouthos are both comprised of uroporphyrin I and uroporphyrin III, but likely differ in the substituents on the porphyrin ring, which can affect color. The substituents are not known. The same porphyrin pigments were also found in colored foot tissue from these species. Conversely, only traces of uroporphyrin were found in the shell of a third species, *Calliostoma zizyphinum* (Calliostomatidae:Trochoidea)*,* despite the fact that it is from the same superfamily and has superficially similar coloration, suggesting that shell color in this species is due to different shell pigments (Williams et al., 2016). The congruence of colors arising from different pigments suggests that there may be selective pressures leading to convergent evolution in these taxa (Williams et al., 2016). Apart from *Clanculus*, uroporphyrin pigments are also responsible for coloration of soft tissues and shells of other (mostly marine) mollusks (reviewed in Williams, 2017), the integument of some annelids (Fox, 1979), and turaco bird feathers (Nicholas & Rimington, 1951).

**Figure 2:**
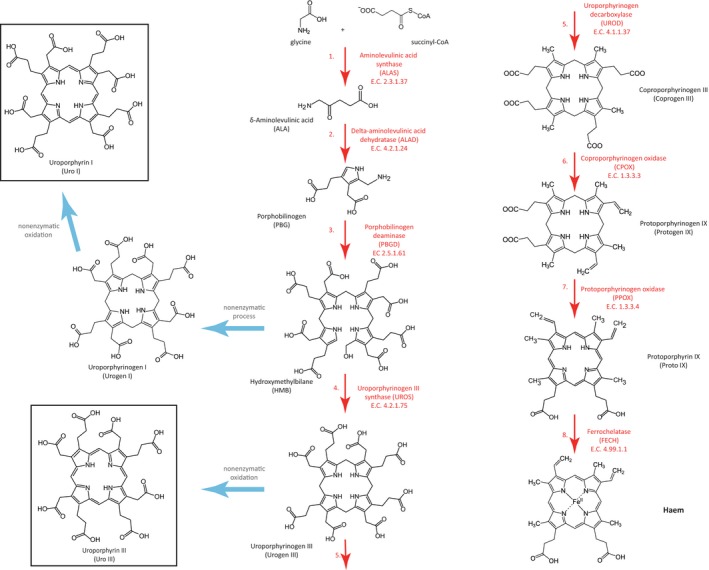
Haem synthetic pathway. The eight enzymatic reactions needed to produce haem and the nonenzymatic side paths resulting in the synthesis of uroporphyrin I and uroporphyrin III pigments (chemical structures marked with a black box). Light blue arrows indicate nonenzymatic processes. Enzyme names are in red font, metabolite names are in black font. Enzyme Commission (EC) numbers provide a numerical classification scheme for enzymes, based on the chemical reactions they catalyze

The identification of shell pigments offers an enormous advantage when searching for genes involved in color synthesis, as some biochemical pathways involved in pigment production are known. In particular, uroporphyrin I and uroporphyrin III are produced in several forms of porphyria, a metabolic disorder affecting humans, and their synthesis has been well studied (Layer, Reichelt, Jahn, & Heinz, 2010). They are synthesized as side products of the evolutionarily ancient heme pathway, which is conserved among metazoans and has been well characterized in humans, *Mus* and *Drosophila* (Ajioka, Phillips, & Kushner, 2006; Heinemann, Jahn, & Jahn, 2008; Figure 2). Normally in the eight‐step haem pathway (Figure 2), the third enzyme porphobilinogen deaminase (PBGD) condenses two molecules of porphobilinogen to form hydroxymethylbilane (HMB), which is highly unstable. The fourth enzyme in the pathway, uroporphyrinogen‐III synthase (UROS) then converts HMB to uroporphyrinogen III by closing the linear tetrapyrrole molecule to form a ring (Ajioka et al., 2006). Uroporphyrin I is produced when activity of UROS is diminished (Warner, Yoo, Roberts, & Desnick, 1992) or the activity of PBGD is increased (Siersema, de Rooij, Edixhoven‐Bosdijk, & Wilson, 1990) leading to an accumulation of HMB. Excess HMB can then be converted into uroporphyrinogen I by the nonenzymatic, spontaneous closure of the ring (Warner et al., 1992). Uroporphyrinogen I can then be oxidised to form uroporphyrin I (Figure 2; Layer et al., 2010). Uroporphyrin III is produced by oxidation of uroporphyrinogen III that builds up when there is reduced activity of the fifth enzyme uroporphyrinogen decarboxylase (UROD), although in humans iron and certain forms of cytochrome P‐450 may also enhance the oxidation of uroporphyrinogen III to uroporphyrin III (Ponka, 1997; Figure 2). The overproduction of intermediate metabolites δ‐aminolevulinic acid, porphobilinogen, and HMB can also enhance the formation of uroporphyrin I, uroporphyrin III, and coproporphyrin III (Hibino, Petri, Buchs, & Ohtake, 2013; Piao, Kiatpapan, Yamashita, & Murooka, 2004). In humans, regulation of the haem pathway occurs primarily at the first step catalyzed by aminolevulinic acid synthase (ALAS), the rate‐limiting enzyme, by downregulating its transcription, upregulating mRNA breakdown, blocking its uptake into mitochondria, and increasing breakdown of the protein (Besur, Hou, Schmeltzer, & Bonkovsky, 2014).

We predict that in porphyrin‐producing tissues in *Clanculus,* the first enzyme in the pathway (ALAS) may be upregulated in tissue producing uroporphyrin I and uroporphyrin III, but UROS or UROD will be downregulated. Our prediction is based on information known about the metabolic disorders known generally as porphyrias. These disorders arise from a deficiency in one of the eight enzymes in the haem pathway and are usually inherited although some forms may be acquired, and environmental factors may play an important role (Balwani & Desnick, 2012). Uroporphyrins are produced in porphyrias with decreased activity of PBGD, UROS, and UROD (Balwani & Desnick, 2012). Upregulation of ALAS has also been associated with increased severity of particular types of porphyria that produce uroporphyrin (To‐Figueras et al., 2011). We do not make a prediction about PBGD as both increased (Siersema et al., 1990) and decreased (Balwani & Desnick, 2012) levels have been associated with uroporphyrin production. Even though UROS and UROD might be downregulated, we still expect all eight genes in the haem pathway to be expressed in all tissues irrespective of porphyrin production, because haem serves as a precursor to cytochrome prosthetic groups which are necessary for cell function (Layer et al., 2010).

In order to test this hypothesis, we used transcriptomics in combination with phylogenetically informed annotation (Speiser et al., 2014) to determine whether *C. margaritarius, C. pharaonius,* and *Ca. zizyphinum* express the enzymes necessary to produce uroporphyrin I and uroporphyrin III. We then used qPCR to compare expression levels within all three species between three tissue types: mantle tissue, which is responsible for shell construction and potentially pigment production, colored foot tissue (in *Clanculus* only), and unpigmented columellar muscle tissue. Our expectations were that in *Clanculus* species, UROS and UROD would be expressed at lower levels in tissue that produces porphyrins (mantle and colored foot), than tissue that does not produce porphyrins (columellar muscle), whereas the reverse might be expected for ALAS. We did not expect to necessarily observe the same pattern in *Ca. zizyphinum*, which has unknown shell pigments.

## 2 MATERIALS AND METHODS

### 2.1 Samples

*Clanculus pharaonius* were collected from Saudi Arabia, *C. margaritarius* were collected from two localities in Japan, and *Calliostoma zizyphinum* specimens came from the United Kingdom (see Table 1 for list of specimens). In Williams et al. (2016), the authors noticed small differences in shell sculpture and pattern in specimens nominally described as *C. margaritarius* and suggested that there may be two groups of uncertain specific status. Given concerns for the possibility of cryptic species, part of the mitochondrial gene cytochrome oxidase was sequenced for *C. margaritarius* specimens #3, 6, 14, and 15 following published methods (Williams & Ozawa, 2006) and compared with homologous transcripts for specimens #1 and 2 in order to confirm species identity.

**Table 1:** Sample details for *Clanculus margaritarius, C. pharaonius,* and *Calliostoma zizyphinum*

Species	#	Sampling locality	Analyses
*C. margaritarius* C	1	Kitahama, Shirahama, Nishimuro‐gun, Wakayama Pref., Japan (CMAR.SHI.1)	HPLC for melanins in shell; confocal on shell (not shown); transcriptome of mantle and foot tissue; COI GenBank accession number: KY200867
*C. margaritarius* A	2	Kitahama, Shirahama, Nishimuro‐gun, Wakayama Pref., Japan (CMAR.SHI.2)	HPLC for melanins in shell; confocal on shell; transcriptome of mantle tissue. Note that this specimen is subadult.
*C. margaritarius* A	3	Kitahama, Shirahama, Nishimuro‐gun, Wakayama Pref., Japan (CMAR.SHI.3)	Confocal on shell (not shown); EDS on shell; HPLC for porphyrins in shell and foot tissue; PCR COI (=AB505297)
*C. margaritarius* A	4	Kitahama, Shirahama, Nishimuro‐gun, Wakayama Pref., Japan (CMAR.APR.1)	Laser ablation; qPCR
*C. margaritarius* A	5	Kitahama, Shirahama, Nishimuro‐gun, Wakayama Pref., Japan (CMAR.APR.2)	Laser ablation; UV‐visible spectrometry; qPCR
*C. margaritarius* A	6	Fukushima, Saeki‐shi, Oita Pref., Japan (CMAR.FUG.1)	HPLC foot (ethanol; not shown); PCR COI (=AB505297)
*C. margaritarius* A	7	Fukushima, Saeki‐shi, Oita Pref., Japan (CMAR.FUG.2)	Raman on shell; confocal on shell (not shown); UV and visible light photograph; laser ablation; NHMUK 20150502
*C. margaritarius* B	8	Not known – unlocalized NHMUK specimen (shell only)	Confocal on shell (not shown); HPLC for melanins on shell
*C. margaritarius* B	9	Not known – unlocalized NHMUK specimen (shell only)	Confocal on shell (not shown); HPLC for porphyrins in shell
*C. margaritarius* B	10	Not known – unlocalized NHMUK specimen (shell only)	Confocal on shell; NHM EDS
*C. margaritarius* B	11	Not known – unlocalized NHMUK specimen (shell only)	UoM EDS; ToF‐SIMS; FTIR; Microfocus Synchrotron
*C. margaritarius* B	12	Not known – unlocalized NHMUK specimen (shell only)	Pigmented layer removed using EDTA; confocal on nacreous layers of shell (not shown); HPLC for porphyrins on dissolved pigment layer (not shown); control shell HPLC for melanins
*C. margaritarius* B	13	Not known – unlocalized NHMUK specimen (shell only)	Confocal on shell (not shown)
*C. margaritarius* A	14	Kitahama, Shirahama, Nishimuro‐gun, Wakayama Pref., Japan (CMAR.SHI.4)	PCR COI (=AB505297)
*C. margaritarius* A	15	Kitahama, Shirahama, Nishimuro‐gun, Wakayama Pref., Japan (CMAR.SHI.5)	PCR COI (=AB505297)
*C. pharaonius*	1	Rose Reef, Thuwal, Saudi Arabia (CPHA.KAU.2)	HPLC for porphyrins in foot; visible/UV photograph; transcriptome of mantle tissue. NHMUK 20150503
*C. pharaonius*	2	Rose Reef, Thuwal, Saudi Arabia (CPHA.KAU.6)	HPLC for porphyrins in shell, qPCR
*C. pharaonius*	3	Rose Reef, Thuwal, Saudi Arabia (CPHA.KAU.4)	HPLC for melanins in shell, qPCR
*C. pharaonius*	4	Rose Reef, Thuwal, Saudi Arabia (CPHA.KAU.1)	Confocal on shell, qPCR
*C. pharaonius*	5	Rose Reef, Thuwal, Saudi Arabia (CPHA.KAU.7)	Laser ablation; UV‐visible spectrometry; qPCR
*C. pharaonius*	6	Rose Reef, Thuwal, Saudi Arabia (CPHA.KAU.3)	qPCR
*C. pharaonius*	7	Rose Reef, Thuwal, Saudi Arabia (CPHA.KAU.5)	qPCR
*Ca. zizyphinum*	1	Shetland Islands, 60°14.9′N, 01°5.1′W, UK (CZIZ.SHT.1)	Confocal on shell (data not shown); transcriptome of mantle tissue. NHMUK 20160315
*Ca. zizyphinum*	2	Shetland Islands, 60°14.9′N, 01°5.1′W, UK (CZIZ.SHT.2)	HPLC for porphyrins in shell (not shown)
*Ca. zizyphinum*	3	Shetland Islands, 60°14.9′N, 01°5.1′W, UK (CZIZ.SHT.q1)	qPCR
*Ca. zizyphinum*	4	Shetland Islands, 60°14.9′N, 01°5.1′W, UK (CZIZ.SHT.q2)	qPCR
*Ca. zizyphinum*	5	Shetland Islands, 60°14.9′N, 01°5.1′W, UK (CZIZ.SHT.q3)	qPCR
*Ca. zizyphinum*	6	Shetland Islands, 60°14.9′N, 01°5.1′W, UK (CZIZ.SHT.q4)	qPCR

Specimen number, sampling locality, analyses undertaken in this study (red font) and in (Williams et al., 2016; black font).

### 2.2 Transcriptome sequencing

Total RNA was extracted from mantle edge tissue from specimens of two *C. margaritarius,* one *C. pharaonius* and one *Ca. zizyphinum,* and from colored tissue from the side of the foot of one specimen of *C. margaritarius*, which is also colored by porphyrins (Williams et al., 2016; Table 1). RNA was extracted using the RNeasy Fibrous Tissue Mini Kit (Qiagen) according to the manufacturer's instructions. Total RNA was quantified using a Qubit^®^ 2.0 Fluorometer RNA assay kit and the RNA integrity assessed on an Agilent 2200 Tapestation using a high sensitivity R6K Screen Tape. Gastropod mRNA was isolated using the Dynabeads^®^ mRNA DIRECT^™^ Micro Kit (Ambion, Life Technologies) according to the 100 ng–1 μg total RNA samples protocol from the manufacturer.

Illumina‐compatible indexed libraries were prepared for each tissue sample using the ScriptSeq^™^ v2 RNA‐Seq Library Preparation Kit from Epicentre (Epicentre Biotechnologies, Madison, WI, USA). The libraries were size checked on an Agilent 2200 Tapestation with a Tapestation HS D1K kit and quantified using qPCR. The libraries were loaded onto a MiSeq V2 500 cycle sequencing run taking one‐fifth of a run according to the manufacturer's instructions. The libraries for *Ca. zizyphinum* and the two *C. margaritarius* mantle tissue samples were then run additionally on a second MiSeq run. All libraries in each run were equimolar.

### 2.3 Transcriptome assembly

Transcriptomes were assembled using tools implemented in Galaxy, an open‐source workflow management system (Blankenberg et al., 2010; Giardine et al., 2005; Goecks, Nekrutenko, & Taylor, 2010). Reads for the two separate sequencing runs were concatenated (where applicable). Filtering removed reads that were identical or differed at <3 bases. Further filtering using Trimmomatic (Lohse et al., 2012) implemented the initial ILLUMINACLIP step (with default options selected), used a sliding window to trim reads (averaging across four bases and requiring an average quality score of 24), and then removed all reads with a length of <30 bases. Transcriptomes were assembled using Trinity (Grabherr et al., 2011), with default settings and a minimum contig length of 200 bases. Finally, ORFs were identified (for nucleotides and predicted amino acid sequences) using the program TransDecoder (Haas et al., 2013; predicted proteins >30 amino acids or longer). Assembly statistics for transcriptomes are given in Table 2. Raw reads for all transcriptomes have been published online NCBI‐SRA SRP092238, SRP092881, and SRP092239.

**Table 2:** Assembly statistics for transcriptomes

Assembly statistics	*Clanculus pharaonius* mantle	*Clanculus margaritarius* C #1 mantle	*Clanculus margaritarius* C #1 foot	*Clanculus margaritarius* A #2 mantle	*Calliostoma zizyphinum* mantle
Minimum contig length	201	201	201	201	201
Maximum contig length	12,989	10,772	12,734	10,772	10,772
Mean contig length	485.23	415.35	417	402.05	444.24
Standard deviation of contig length	475.35	344.32	353.33	321.35	371.52
Median contig length	332	307	307	298	323
N50 contig length	541	434	435	415	481
Number of contigs	90,248	113,249	129,021	85,061	118,165
Number of contigs ≥1 kb	6,884	5,090	5,967	3,547	6,761
Number of contigs in N50	20,970	30,436	34,360	23,060	30,427
Number of bases in all contigs	43,791,415	47,038,272	53,801,440	34,198,859	52,494,157
Number of bases in contigs ≥1 kb	11,897,645	8,110,215	9,547,572	5,514,410	10,673,841
GC Content of contigs (%)	40.49	39.74	39.40	39.35	40.99

### 2.4 Identification of genes associated with pigment synthesis

We searched for potential orthologs of the eight genes in the haem pathway using phylogenetically informed annotation (PIA), a tree‐based approach for annotating transcriptomes and genomes (Speiser et al., 2014). Briefly, PIA involves a set of precalculated gene trees produced using tools for phylogenetic analysis by maximum likelihood available in the Osiris package for Galaxy (Oakley et al., 2014). These trees incorporated sequences from the predicted protein databases associated with 29 fully sequenced genomes, including those from 24 metazoans, two choanoflagellates, and three fungi (see Speiser et al., 2014 for details). Each analysis also included “landmark” sequences from GenBank, which represent genes that have been well characterized functionally. The BLASTp algorithm searched translated versions of the transcriptomes using the same queries that were used to identify sequences for the precalculated gene trees described above. For searches using BLASTp, the ten hits that had the lowest e‐value scores were retained, provided that these e‐values were below 1e−4. Next, MAFFT‐profile (Katoh & Standley, 2013) was used to align the hits from BLAST searches against the sequences that comprised the precalculated gene trees. Finally, the evolutionary placement algorithm (Berger, Krompass, & Stamatakis, 2011; Berger et al., 2010; implemented in RAxML; Stamatakis, 2014) was used to place hits from the BLAST searches on to the precalculated gene trees using maximum likelihood. Hits from the transcriptomes were scored as potential orthologs of genes if they: (1) had an orthologous relationship to genes with well‐characterized functions and (2) fell on short branches that were in positions consistent with established relationships between species. Apparent multiple hits were conservatively scored only once because without a completely sequenced genome it is not possible to tell whether these sequences represent different loci, different alleles or isoforms of the same locus, or sequence or assembly errors. For comparison, we also performed BLASTx searches against all sequences on GenBank, recording the top score with an identified gene.

### 2.5 Quantitative analysis of gene expression

Total RNA was extracted from columellar, mantle, and colored foot tissue samples using the RNeasy Fibrous Tissue Mini Kit (Qiagen) according to the manufacturer's protocol from *C. pharaonius* (*n* = 6 individuals), *C. margaritarius* A (*n* = 2 individuals), and *Ca. zizyphinum* (*n* = 4 individuals, no colored foot tissue). This kit includes DNAse treatment to eliminate genomic DNA contamination. cDNA synthesis was carried out using 30 ng total RNA from each individual using QuantiTect Reverse Transcription Kit, (Qiagen) according to the manufacturer's protocol. This kit also has an integrated genomic DNA removal step prior to cDNA synthesis.

Specific primers were designed to amplify the five haem genes using Primer3Plus (Untergasser et al., 2007). One set of primers worked for both *Clanculus* species and a separate set of primers were designed for *Calliostoma* (Table 3). We used 18S as the reference gene and the same amount of RNA (quantified by nanodrop) was used for each cDNA synthesis, with all giving very similar *C*_T_ results for 18S. Primers were designed for 18S that worked on all species.

**Table 3:** Primer pairs used for qPCR, amplicon product size, and PCR annealing temperature

Gene	Forward primer	Reverse primer	Product size (bp)	Annealing temperature (°C)
*Clanculus* (2 species)				
ALAS	CACAGCCCCAGTCACATCAT	AGTTGGGGCCACACGAAGTC	156	61
ALAD	CGGATCACGCAGTTCTTCAC	AAGTGGTTCAATGGCTTCTTGTAG	200	61
PBGD‐1	GGCCCCGAATATGAGAAGAG	CTCTCGGCGACGACTCTGAT	170	61
UROS	TGAATTTGCAGTGTTCTTCAGTCC	TGGTTCTGGTTTGGCTGTAA	172	61
UROD	GGTTATCCCCCTTGCCTTG	ACCCAGCTCCTTGTGAATATCA	133	61
*Calliostoma*				
ALAS	GTGCCTAAAATTGTTGCCTTTGA	CCCACAACATAGCCTCCCATATT	245	61
ALAD	ATGTCACGGACACTGTGGTATTC	AGGTCCATAAAAACTGGATGCAAA	241	65
PBGD‐2	AGACCTGCCCACATCACTTC	ACCCACAACACTTCCCTCTG	166	61
UROS	GGATTGCCCGAGTTTGCAGT	GGCTGTGATACCATGGAGTTTGAA	168	64
UROD	TTTTGGTTATCCCCCTTGC	GGCTTCATACACATAGCCCAGTTC	152	59
All species				
18S	AAACGGCTACCACATCCAAG	CCAGACTTGCCCTCCAATAG	165	57

Two divergent sequences of PBGD easily separable by alignment of amino acids were found in transcriptomes of *C. pharaonius* mantle and *C. margaritarius* A #1 mantle. As PBGD is known to exist as two isoforms in humans (Deybach & Puy, 1995), primers were made specifically for PBGD‐1 for *Clanculus* (the most highly expressed isoform in this species where the two isoforms were found) and PBGD‐2 for *Calliostoma* (the only isoform expressed in this species). Primers for PBGD‐1 and PBGD‐2 were designed to match to regions that varied between the two isoforms, so that amplification of only the target isoform was possible (see Appendix S1 for primer positions in gene alignments).

PCRs of 20 μl contained Power SYBR^®^ Green Master Mix according to the manufacturers protocol (Applied Biosystems), 10 pmol of each primer, and 2 μl of 1/10 dilution of cDNA synthesized from individual snails. PCR cycling conditions were as follows: 50°C for 2 min, 95°C for 10 min, then 40 cycles of 95°C for 30 s, X°C for 1 min, using the CFX96 real‐time PCR detection system (Bio‐Rad), where X is the optimized annealing temperature for each primer pair (see Table 3). A dissociation curve was generated in each case to check that only a single band was amplified. The qPCRs were performed in triplicate for each individual snail/tissue and normalized to 18S using qGene (Muller, Janovjak, Miserez, & Dobbie, 2002) taking into account amplification efficiency, which was calculated using a dilution series for each primer pair (Pfaffl, 2001). 18S was chosen as a commonly used reference gene, and no consistent variation was found in its expression between tissue types or species. The individual mean normalized gene expression levels between tissues within each species were compared for each gene using paired two‐tail Student's *t* tests performed in Excel (T.TEST function).

## 3 RESULTS

### 3.1 Deep divergence in *C. margaritarius*

Two divergent genetic lineages were found in *C. margaritarius*, here referred to as “A” or “C”. A third morphologically divergent group referred to as “B” was identified in (Williams et al., 2016). We were not able to include *C. margaritarius* B in this study as only dry shells are available and there are no tissue samples for genetic studies. All COI sequences corresponding to *C. margaritarius* A were identical to each other and to a published COI sequence on GenBank for the same species (AB505297), but differed from the single specimen of *C. margaritarius* C by 22 synonymous substitutions over the 658 bp used as a barcode in most molluskan studies (GenBank accession number for *C. margaritarius* C: KY200867; photographs showing exemplar shells in Appendix S2). This difference (~3.3% uncorrected) was unexpected and may reflect cryptic species or highly divergent populations. Further studies are needed to confirm the status of these groups. Both *C. margaritarius* A and C were included in transcriptomics studies, but only *C. margaritarius* A was used in qPCR studies as no further specimens of *C. margaritarius* C were available.

### 3.2 Identification of genes associated with pigment synthesis

**Figure 3:**
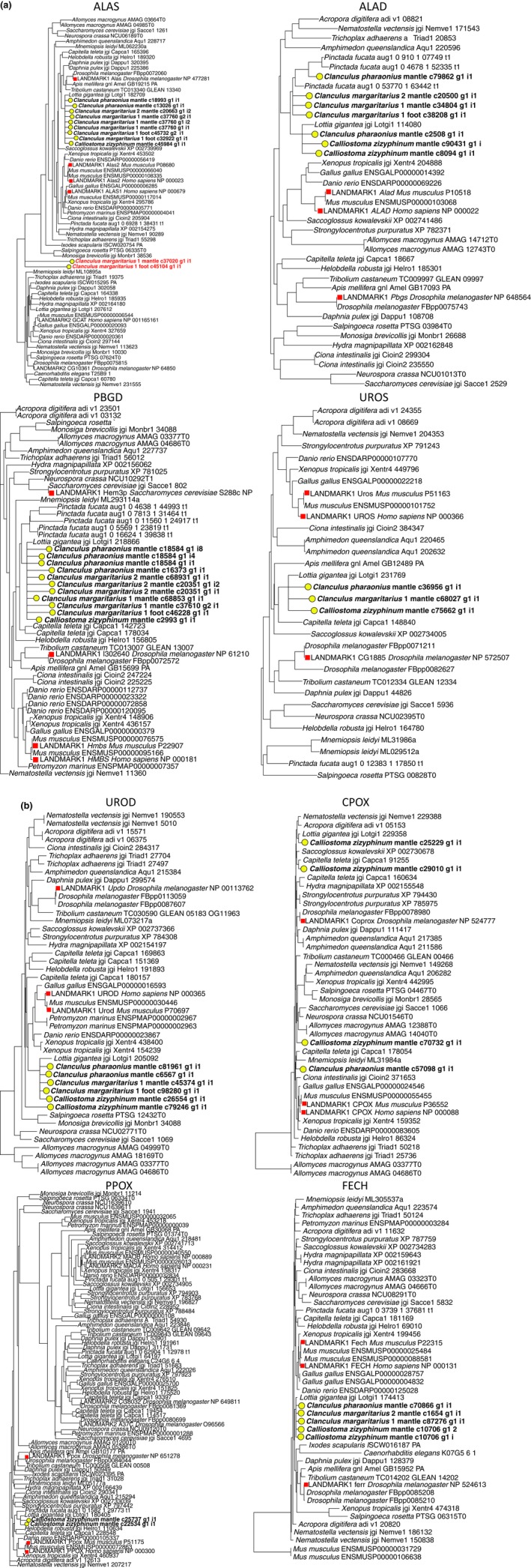
Maximum likelihood trees for enzymes used in the haem pathway. Sequences in each tree are from predicted protein databases associated with complete genomes. The exceptions are sequences marked “LANDMARK” and highlighted with red squares; these are sequences from GenBank that have been well characterized functionally. Sequences highlighted with yellow circles represent assembled transcripts from our transcriptomes. (a) First four enzymes in the haem biosynthetic pathway. Enzymes are aminolevulinic acid synthase, aminolevulinic acid dehydratase, porphobilinogen deaminase, and uroporphyrinogen‐III synthase. (b) Last four enzymes in the pathway. Enzymes are uroporphyrinogen decarboxylase, coproporphyrinogen oxidase, protoporphyrinogen oxidase, and ferrochelatase. Sequences in red font are likely not haem genes

We used transcriptomics in combination with phylogenetically informed annotation (Speiser et al., 2014) to determine whether *C. margaritarius, C. pharaonius,* and *Ca. zizyphinum* express the enzymes necessary to produce uroporphyrin I and uroporphyrin III. Transcripts that code for enzymes comprising the haem synthesis pathway (Figure 2) were expressed in all species and tissues, but all eight genes were found only in *Ca. zizyphinum* mantle (Figure 3; Table 4; Appendix S3). Transcripts corresponding to the first three enzymes (aminolevulinic acid synthase—ALAS, aminolevulinic acid dehydratase—ALAD, porphobilinogen deaminase—PBGD) were found in all transcriptomes, for all species (Figure 3; Table 4; Appendix S3). We did not limit the number of potential orthologues, and in some cases, more than one was identified. These orthologues were then included in a phylogenetic analysis including published sequences from mollusks and other organisms in order to confirm their evolutionary relationships. For comparison, we also performed BLASTx searches against all sequences in GenBank, recording the top hit with an identified gene (Table 5). All transcripts were most similar to molluskan or invertebrate haem genes as expected, with only two exceptions (Table 5); two transcripts identified in the PIA analysis of ALAS were more similar to serine palmitoyltransferase than ALAS (marked with red font in Figure 3a).

**Table 4:** Summary showing the presence or absence of transcripts corresponding to the eight haem genes

Gene/transcriptome	*Clanculus pharaonius* mantle	*Clanculus margaritarius* 1 mantle	*Clanculus margaritarius* 1 foot	*Clanculus margaritarius* 2 mantle	*Calliostoma zizyphinum* mantle
ALAS	+	+	+	+	+
ALAD	+	+	+	+	+
PBGD	+	+	+	+	+
UROS	+	+	−	−	+
UROD	+	+	+	−	+
CPOX	o	−	−	−	+
PPOX	−	−	−	−	+
FECH	+	+	−	+	+

+, sequence identified and clusters with other molluskan sequences; −, no sequences found in transcriptome; o, transcript found but does not cluster with other molluskan sequences.

**Table 5:** Details for BLASTx hits for transcripts identified in PIA analyses as putative haem genes

Transcripts	Gene	Species	GenBank Acc. No.	Conserved domains	Other conserved regions
*ALAS transcripts*					
Clanculus_margaritarius_1_mantle_c37020_g1_i1	Serine palmitoyltransferase 2‐like	*Aplysia californica* (gastropod)	XP_012942343	PLN02483, AAT_I superfamily	Pyridoxal 5′‐phosphate‐binding site, catalytic residue
Clanculus_margaritarius_1_mantle_c37760_g1_i1	5‐aminolevulinate synthase, erythroid‐specific, mitochondrial‐like	*Aplysia californica* (gastropod)^a^	XP_005104982	AAT_I superfamily	–
Clanculus_margaritarius_1_mantle_c37760_g1_i2	5‐aminolevulinate synthase, erythroid‐specific, mitochondrial‐like	*Aplysia californica* (gastropod)^a^	XP_005104982	AAT_I superfamily	–
Clanculus_margaritarius_1_mantle_c37760_g2_i1	5‐aminolevulinic acid synthase	*Scylla paramamosain* (decapod)	AEI88095	AAT_I superfamily	–
Clanculus_margaritarius_1_foot_c32922_g1_i1	5‐aminolevulinate synthase, erythroid‐specific, mitochondrial‐like isoform X1	*Lingula anatina* (brachiopod)	XP_013397246	AAT_I superfamily Preseq_ALAS superfamily	–
Clanculus_margaritarius_1_foot_c45104_g1_i1	Serine palmitoyltransferase 2‐like	*Aplysia californica* (gastropod)	XP_012942343	PLN02483, AAT_I superfamily	Pyridoxal 5′‐phosphate‐binding site, catalytic residue
Clanculus_margaritarius_1_foot_c45732_g2_i1	5‐aminolevulinate synthase, erythroid‐specific, mitochondrial‐like	*Aplysia californica* (gastropod)^a^	XP_005104982	AAT_I superfamily	Catalytic residue
Clanculus_margaritarius_2_mantle_c20663_g1_i2	5‐aminolevulinate synthase, erythroid‐specific, mitochondrial‐like isoform X1	*Mizuhopecten yessoensis* (bivalve)	XP_021356935	AAT_I superfamily	Catalytic residue
Calliostoma_zizyphinum_mantle_c45984_g1_i1	5‐aminolevulinate synthase, erythroid‐specific, mitochondrial‐like isoform X1	*Mizuhopecten yessoensis* (bivalve)^a^	XP_021356935	AAT_I superfamily	Catalytic residue, pyridoxal 5′‐phosphate‐binding pocket
Clanculus_pharaonius_mantle_c13026_g1_i1	5‐aminolevulinate synthase, erythroid‐specific, mitochondrial‐like isoform X1	*Lingula anatina* (brachiopod)	XP_013397246	Preseq_ALAS, AAT_I superfamily	–
Clanculus_pharaonius_mantle_c18993_g1_i1	5‐aminolevulinate synthase, erythroid‐specific, mitochondrial‐like	*Aplysia californica* (gastropod)^a^	XP_005104982	AAT_I superfamily	Catalytic residue
*ALAD transcripts*					
Clanculus_margaritarius_1_mantle_c34804_g1_i1	Delta‐aminolevulinic acid dehydratase‐like	*Mizuhopecten yessoensis* (bivalve)	XP_021366088	ALAD‐PBGS superfamily	Active site, Schiff base residues
Clanculus_margaritarius_1_foot_c38208_g1_i1	Delta‐aminolevulinic acid dehydratase‐like	*Mizuhopecten yessoensis* (bivalve)	XP_021366088	ALAD‐PBGS superfamily	Active site, Schiff base residues
Clanculus_margaritarius_2_mantle_c20500_g1_i1	delta‐aminolevulinic acid dehydratase‐like	*Mizuhopecten yessoensis* (bivalve)	XP_021366088	ALAD‐PBGS superfamily	Active site, Schiff base residues
Calliostoma_zizyphinum_mantle_c8094_g1_i1	delta‐aminolevulinic acid dehydratase‐like	*Biomphalaria glabrata* (gastropod)^a^	XP_013070705	ALAD‐PBGS superfamily	–
Calliostoma_zizyphinum_mantle_c90431_g1_i1	delta‐aminolevulinic acid dehydratase‐like	*Mizuhopecten yessoensis* (bivalve)	XP_021366088	ALAD‐PBGS superfamily	–
Clanculus_pharaonius_mantle_c2508_g1_i1	delta‐aminolevulinic acid dehydratase‐like	*Biomphalaria glabrata* (gastropod)	XP_013070705	ALAD‐PBGS superfamily	Schiff base residues
Clanculus_pharaonius_mantle_c79862_g1_i1	delta‐aminolevulinic acid dehydratase‐like	*Mizuhopecten yessoensis* (bivalve)	XP_021366088	ALAD‐PBGS superfamily	–
*PBGD transcripts*					
Clanculus_margaritarius_1_mantle_c37610_g2_i1	Porphobilinogen deaminase	*Crassostrea gigas* (bivalve)^a^	XP_011444315	Type 2 periplasmic binding fold superfamily	–
Clanculus_margaritarius_1_mantle_c68853_g1_i1	Porphobilinogen deaminase‐like isoform X2	*Biomphalaria glabrata* (gastropod)	XP_013082888	Type 2 periplasmic binding fold superfamily	–
Clanculus_margaritarius_1_foot_c46228_g1_i1	Porphobilinogen deaminase	*Crassostrea gigas* (bivalve)	XP_011444315	Type 2 periplasmic binding fold superfamily	–
Clanculus_margaritarius_2_mantle_c20351_g1_i1	Porphobilinogen deaminase	*Crassostrea gigas* (bivalve)^a^	XP_011444315	Type 2 periplasmic binding fold superfamily	–
Clanculus_margaritarius_2_mantle_c20351_g1_i2	Porphobilinogen deaminase	*Crassostrea gigas* (bivalve)^a^	XP_011444315	Type 2 periplasmic binding fold superfamily	–
Clanculus_margaritarius_2_mantle_c68931_g1_i1	Porphobilinogen deaminase	*Crassostrea gigas* (bivalve)	XP_011444315	Type 2 periplasmic binding fold superfamily	–
Calliostoma_zizyphinum_mantle_c2993_g1_i1	Porphobilinogen deaminase	*Crassostrea gigas* (bivalve)	XP_011444315	Type 2 periplasmic binding fold superfamily	–
Clanculus_pharaonius_mantle_c16373_g1_i1	Porphobilinogen deaminase	*Python bivittatus* (reptile)^a^	XP_007441554	Type 2 periplasmic binding fold superfamily	–
Clanculus_pharaonius_mantle_c18584_g1_i1	Porphobilinogen deaminase‐like isoform X2	*Biomphalaria glabrata* (gastropod)	XP_013082888	Type 2 periplasmic binding fold superfamily	–
Clanculus_pharaonius_mantle_c18584_g1_i4	Porphobilinogen deaminase‐like isoform X2	*Biomphalaria glabrata* (gastropod)	XP_013082888	Type 2 periplasmic binding fold superfamily	–
Clanculus_pharaonius_mantle_c18584_g1_i8	Porphobilinogen deaminase‐like	*Aplysia californica* (gastropod)	XP_005097343	Type 2 periplasmic binding fold superfamily	–
*UROS transcripts*					
Clanculus_margaritarius_1_mantle_c68027_g1_i1	Uroporphyrinogen‐III synthase‐like isoform X2	*Aplysia californica* (gastropod)	XP_005098107	HemD superfamily	–
Calliostoma_zizyphinum_mantle_c75662_g1_i1	Uroporphyrinogen‐III synthase‐like isoform X2	*Aplysia californica* (gastropod)^a^	XP_005098107	HemD superfamily	–
Clanculus_pharaonius_mantle_c36956_g1_i1	Uroporphyrinogen‐III synthase‐like isoform X2	*Aplysia californica* (gastropod)^a^	XP_005098107	HemD superfamily	–
*UROD transcripts*					
Clanculus_margaritarius_1_mantle_c45374_g1_i1	Uroporphyrinogen decarboxylase‐like	*Aplysia californica* (gastropod)^a^	XP_005105887	URO‐D_CIMS_like protein superfamily	–
Clanculus_margaritarius_1_foot_c98280_g1_i1	Uroporphyrinogen decarboxylase‐like	*Biomphalaria glabrata* (gastropod)^a^	XP_013083863	URO‐D_CIMS_like protein superfamily	–
Calliostoma_zizyphinum_mantle_c26554_g1_i1	Uroporphyrinogen decarboxylase‐like	*Aplysia californica* (gastropod)^a^	XP_005105887	URO‐D_CIMS_like protein superfamily	–
Calliostoma_zizyphinum_mantle_c79246_g1_i1	Uroporphyrinogen decarboxylase	*Clonorchis sinensis* (platyhelminth)^b^	GAA51972	URO‐D_CIMS_like protein superfamily	–
Clanculus_pharaonius_mantle_c6567_g1_i1	Uroporphyrinogen decarboxylase‐like	*Aplysia californica* (gastropod)^a^	XP_005105887	URO‐D_CIMS_like protein superfamily	–
Clanculus_pharaonius_mantle_c81961_g1_i1	Uroporphyrinogen decarboxylase‐like	*Aplysia californica* (gastropod)^a^	XP_005105887	URO‐D_CIMS_like protein superfamily	–
*CPOX transcripts*					
Calliostoma_zizyphinum_mantle_c25229_g1_i1	Oxygen‐dependent coproporphyrinogen‐III oxidase‐like	*Octopus bimaculoides* (octopus)	XP_014771663	–	–
Calliostoma_zizyphinum_mantle_c29010_g1_i1	Coproporphyrinogen‐III oxidase	*Mizuhopecten yessoensis* (bivalve)	OWF48267	Coprogen oxidase superfamily	–
Calliostoma_zizyphinum_mantle_c70732_g1_i1	Coproporphyrinogen‐III oxidase	*Opisthorchis viverrini* (platyhelminth)^b^	OON21227	Coprogen oxidase superfamily	–
Clanculus_pharaonius_mantle_c57098_g1_i1	Oxygen‐dependent coproporphyrinogen‐III oxidase‐like	*Octopus bimaculoides* (octopus)	XP_014771663	–	–
*PPOX transcripts*					
Calliostoma_zizyphinum_mantle_c22534_g1_i1	Protoporphyrinogen oxidase‐like	*Aplysia californica* (gastropod)	XP_005096895	HemY superfamily	–
Calliostoma_zizyphinum_mantle_c25737_g1_i1	Protoporphyrinogen oxidase‐like	*Aplysia californica* (gastropod)	XP_005096895	HemY superfamily	–
*FECH transcripts*					
Clanculus_margaritarius_1_mantle_c87276_g1_i1	Ferrochelatase, mitochondrial‐like	*Aplysia californica* (gastropod)	XP_005090207	Ferrochelatase superfamily	–
Clanculus_margaritarius_2_mantle_c1654_g1_i1	Ferrochelatase, mitochondrial‐like	*Aplysia californica* (gastropod)	XP_005090207	Ferrochelatase superfamily	–
Calliostoma_zizyphinum_mantle_c10706_g1_i1	Ferrochelatase, mitochondrial‐like	*Aplysia californica* (gastropod)^a^	XP_005090207	Ferrochelatase superfamily	–
Calliostoma_zizyphinum_mantle_c10706_g1_i2	Ferrochelatase, mitochondrial‐like	*Aplysia californica* (gastropod)^a^	XP_005090207	Ferrochelatase superfamily	–
Clanculus_pharaonius_mantle_c70866_g1_i1	Ferrochelatase, mitochondrial	*Crassostrea gigas* (bivalve)^a^	EKC30122	Ferrochelatase superfamily	–

Details listed are gene identification, species identification, GenBank accession number, conserved domains, and any other conserved regions identified. Nucleotide sequences are in Appendix S3. Transcripts in red font did not cluster with other molluskan sequences and are not haem genes.

a Top hit is *Lottia gigantea* (gastropod) hypothetical protein.

b Top hit is *Opisthorchis viverrini* (platyhelminth) hypothetical protein.

No transcripts corresponding to uroporphyrinogen‐III synthase (UROS) were identified in transcriptomes from *C. margaritarius* C #1 foot or *C. margaritarius* A #2 mantle, and uroporphyrinogen decarboxylase (UROD) was also missing from the latter (Figure 3). No sequences corresponding to coproporphyrinogen oxidase (CPOX) were recovered from *C. margaritarius* transcriptomes, protoporphyrinogen oxidase (PPOX) was missing from all *Clanculus* transcriptomes, and ferrochelatase (FECH) was missing from *C. margaritarius* C #1 foot (Figure 3). Predicted translated sequences of the identified haem synthesis genes showed a high degree of amino acid conservation with human proteins, which have been characterized experimentally (Appendix S1; sequences in Appendix S3).

In all trees, transcripts corresponding to potential orthologues of haem synthesis genes form clusters with each other and with published genomic sequences for the limpet *Lottia gigantea* and pearl oyster *Pinctada fucata*, and with landmark sequences from other taxa, which represent genes that have been characterized experimentally (Figure 3). The only exceptions were in the CPOX and ALAS trees. In the ALAS tree, all our transcripts, except *Clanculus margaritarius* 1 mantle c37020 g and *Clanculus margaritarius* 1 foot c45104 g1 cluster together, and BLASTx results suggest that these are not haem genes, confirming PIA results. Primers used in qPCR were designed to avoid amplification of these two sequences. The four transcripts initially identified as CPOX do not come out in a cluster, and only one, *Calliostoma zizyphinum* mantle c25229 g1 i1, clusters with a published molluskan transcript (*Lottia gigantea*); however, BLASTx results suggest that all are CPOX genes, although they may represent different loci.

### 3.3 Quantification of gene expression levels

**Figure 4:**
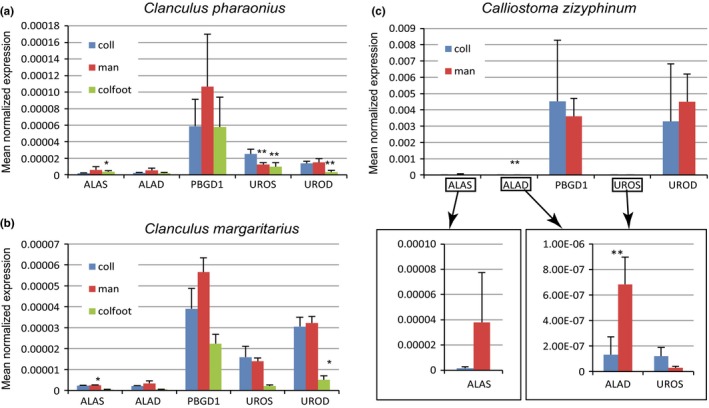
Relative expression levels for the first five genes in the heme synthesis pathway compared between tissues predicted to produce porphyrin (*Clanculus* mantle (man) & coloured foot (colfoot)) versus those that are not (*Clanculus* columellar muscle (coll)). *Calliostoma zizyphinum* is included as a negative control as none of its tissues are predicted to produce porphyrins. Normalised expression was calculated relative to expression of 18S and comparisons were made between columellar tissue and mantle and between columellar tissue and coloured foot. The P values from a students t‐test demonstrated * < 0.05 or ** < 0.01 significance. (a) *Clanclus pharaonius*. (b) *Clanclus margaritarius*. (c) *Calliostoma zizyphinum*. The smaller boxed graphs for *Calliostoma zizyphinum* show the same data, but with a changed y‐axis to allow the lower normalised gene expression levels to be visualised.

Quantitative real‐time PCR (qPCR) was used to estimate the expression levels of transcripts for the first five enzymes in the haem synthesis pathway (Figure 2) in columellar muscle, colored foot tissue, and mantle tissue from *C. pharaonius, C. margaritarius* A, and *Ca. zizyphinum* (no foot tissue; Figure 4, Appendix S4). Mantle tissue was tested because it is responsible for shell construction and potentially pigment production, if pigments are produced de novo by the animal. Colored foot tissue was also used because previous studies have shown that the two *Clanculus* species used in this study also produce uroporphyrin I and III in colored foot tissue (Williams et al., 2016). Our expectations were that in *Clanculus* species, UROS and UROD would be expressed at lower levels in tissue that produces porphyrins (mantle and colored foot) than tissue that does not produce porphyrins (columellar muscle). In humans, an upregulation of the first gene in the haem pathway (ALAS) has been associated with increased severity of particular types of porphyria that produce uroporphyrin (To‐Figueras et al., 2011), so an increase in ALAS, along with a reduction in UROS and/or UROD would also be consistent with porphyrin production. We did not expect to necessarily observe the same patterns in *Ca. zizyphinum*, which has only trace amounts of uroporphyrin I and III in the shell (Williams et al., 2016).

Our results are consistent with the idea that *Clanculus* species are producing uroporphyrin I and III de novo in colored foot and mantle tissue. Several comparisons of relative levels of gene expression between tissue types (comparing putatively porphyrin‐producing tissues versus control, nonporphyrin‐producing tissue) were statistically significant using two‐tail Student's *t* tests (see Table 6 for *p* values). Namely, in *C. pharaonius*, both colored foot and mantle tissue show significantly lower expression levels than columellar muscle for UROS (*p* < .01), as does colored foot for UROD (*p* < .001). Conversely, ALAS levels were higher in colored foot than columellar muscle (*p* < .05). Similarly, in *C. margaritarius*, expression levels were lower in colored foot than columellar muscle for both UROS and UROD, although only the value for UROS was significant (*p* < .05). Values of ALAS were also significantly different between columellar muscle and mantle (*p* < .05), with ALAS upregulated in mantle. Conversely, in *Ca. zizyphinum,* only ALAD was significantly upregulated in mantle versus columellar muscle (*p* < .05), which is not consistent with the production of uroporphyrin.

**Table 6:** Probabilities for two‐tailed, paired *t* tests for differences in gene regulation among tissues in three trochoidean species of gastropod

Species	Tissue comparisons	ALAS	ALAD	PBGD	UROS	UROD
*Clanculus margaritarius*	Columellar muscle versus mantle	0.03667*	0.28708	0.07258	0.57494	0.79846
	Columellar muscle versus colored foot	0.09060	0.08156	0.34498	0.07430	0.04432*
*Clanculus pharaonius*	Columellar muscle versus mantle	0.05125	0.07516	0.23290	0.00531**	0.47822
	Columellar muscle versus colored foot	0.02389*	0.12462	0.90237	0.00050**	0.00003**
*Calliostoma zizyphinum*	Columellar muscle versus mantle	0.16621	0.01222*	0.54429	0.07822	0.59242

Significant values are marked with an asterisk.

## 4 DISCUSSION

### 4.1 Biosynthesis of uroporphyrin I and III

Understanding the evolution of shell color first requires identification of pigments and then linking those pigments to a biosynthetic pathway. In this study, we investigated the biosynthesis of two dominant shell porphyrin pigments that have recently been found to contribute to shell color in the marine snails *Clanculus pharaonius* and *C. margaritarius*. The shell pigments uroporphyrin I and uroporphyrin III are produced as side products of the haem pathway (Hendry & Jones, 1980). Therefore, we expected to find differences in the expression of the haem pathway genes in tissues producing uroporphyrins when compared to nonporphyrin‐producing tissues in *Clanculus* species. We identified the first four genes of the haem pathway, which are necessary for the production of uroporphyrin I and III, in at least one transcriptome from each of the three study species, although the fourth enzyme, UROS, was not recovered in all tissues. All four remaining genes in the haem pathway were also identified in *Ca. zizyphinum* but were not recovered from all *Clanculus* transcriptomes. In two transcriptomes (*C. margaritarius* C #1 foot and *C. margaritarius* A #2 mantle), transcripts for UROS were not recovered, and UROD was not recovered from *C. margaritarius* A #2 mantle. The absence of transcripts corresponding to some haem genes does not mean these genes are completely absent, as the synthesis of haem is essential for life, because haem serves as a precursor to cytochrome prosthetic groups which are necessary for cell function (Layer et al., 2010). Our inability to find some transcripts corresponding to some genes may suggest either low levels of expression, or that the depth of sequencing was insufficient, or that sequences were too divergent to be recognized using BLAST‐based searches.

Comparison of relative expression levels between tissue types demonstrated differences in gene expression levels between pigmented and nonpigmented tissues consistent with our hypothesis. This was most pronounced in *C. pharaonius* colored foot tissue where the first enzyme in the haem pathway (ALAS) was significantly upregulated, and UROS and UROD were significantly downregulated. Based on human clinical trials, increased levels of ALAS, along with decreased levels of UROS and UROD would likely result in increased production of uroporphyrin (To‐Figueras et al., 2011). UROS was also significantly downregulated in mantle tissue versus columellar muscle. ALAS levels were also higher in mantle than columellar muscle, although the values were marginally nonsignificant (*p* = .05125; Table 6).

The second species, *C. margaritarius,* showed similar differences, but, perhaps due to only being able to obtain a few samples of this rare species, only the upregulation of ALAS in mantle and downregulation of UROS in colored foot (both compared to collumellar tissue) were statistically significant. The effect on uroporphyrin production of an increase in activity of ALAS, without a concomitant decrease in UROS or UROD, is not known, and further samples are needed to see whether this result is typical. Even with the caveat that numbers of individuals examined were small, the results for the colored foot are consistent with our expectations for porphyrin‐producing tissues.

Contrary to observations for *Clanculus*, expression levels of haem genes did not differ significantly between mantle and columellar muscle from *Ca. zizyphinum*, with the exception of ALAD, which was expressed more highly in mantle tissue. Increased activity of ALAD is not associated with production of uroporphyrins, although reduced activity is associated with some forms of porphyria (Balwani & Desnick, 2012). We did not expect to observe the same patterns of haem gene expression in *Ca. zizyphinum*, a species that does not produce shell porphyrins. The shell pigments of *Ca. zizyphinum* are unknown, although based on the observation that some pigments appear to be phylogenetically distributed (Williams, 2017), they may include bilins, which are found in other trochoidean families (Tixier, 1952). Bilins may be taken up in the diet (Fox, 1979; MacColl et al., 1990) or produced by the organism through the breakdown of hemoproteins (Bandaranayake, 2006; Hudson & Smith, 1975), and the pathways for production of trochoidean bilins are unknown.

Taken together, the genetic data presented here along with previously published chemical data that identified shell pigments as uroporphyrin I and III (Williams et al., 2016) support the suggestion that shell porphyrins are produced de novo by two species of *Clanculus*. An interesting side effect of the incorporation of trochopuniceus and trochoxouthos into the shell and colored foot tissue of the two *Clanculus* species is that the production of haem may be reduced in mantle and foot tissues as neither uroporphyrin I nor uroporphyrin III can be converted into heme. Earlier authors (Comfort, 1949; Hendry & Jones, 1980; Kennedy, 1975) noted that molluskan taxa that have uroporphyrin I in shell and integument generally do not use hemoglobin as their respiratory pigment. Many mollusks, including many vetigastropods, use hemocyanin, which, despite the name, is not synthesized via the haem biosynthetic pathway (Hanzlik, 1976). It has been suggested that the haem biosynthetic pathway is likely of lesser importance in such organisms and therefore that haem production can be reduced without harm to the animal (Hendry & Jones, 1980).

### 4.2 Diet versus de novo synthesis—evidence from other studies

Although the evidence obtained in this study suggests that porphyrin shell pigmentation is produced de novo by the animal, the origin of porphyrin pigments in molluskan shells was considered uncertain throughout the last century (Fox, 1976). For instance, Comfort (1950) suggested that the uroporphyrin I found in vetigastropod shells is derived from the animal's diet, but tellingly, admitted that there was no evidence that uroporphyrins can be synthesized from chlorophyll (a porphyrin derivative), and no relationship was observed between pigment type and molluskan feeding mode.

Two more recent studies by Underwood and Creese also suggested that shell uroporphyrin is derived from the diet. Underwood and Creese (1976) showed that the width of black bands on shells of the trochid *Austrocochlea porcata* (as *A. constricta*) differed between estuary and open coast habitats and was correlated with chlorophyll availability at each site. These authors detected and measured uroporphyrin I in the shells and showed that concentrations were also correlated with shell banding pattern (Creese & Underwood, 1976). On the basis of their results, the authors suggested that shell pigmentation was not under genetic control but rather affected by environmental factors (Creese & Underwood, 1976). The authors did not, however, show that the distribution of uroporphyrin I was congruent with pigmentation patterns, although they did show that the black pigment dissolved in acid, as would be expected for a porphyrin. However, shells of this species examined under ultraviolet light do not demonstrate the strong fluorescence seen in *Clanculus*. It is possible that uroporphyrin occurs in the shell without contributing color, and that instead banding patterns are due to bilins (also known as bile pigments); both classes of shell pigments have been found to co‐occur in another trochoidean, *Cittarium pica* (Comfort, 1951). A biliprotein (bilin complexed to a protein) has also been found in foot and shell of *Phorcus turbinatus* (as *Monodonta turbinata*; Bannister, Bannister, & Micallef, 1968a,b, 1970), a species from the same subfamily as *Austrocochlea*. Unlike porphyrins, bilins can be obtained from the diet. For instance, the bilin portions of three biliproteins in the ink of the sea hare *Aplysia californica* are derived from bilins occurring as biliproteins in red algae consumed by the animals (MacColl et al., 1990). Another (not mutually exclusive) explanation is that the synthesis of pigmentation in the banding pattern occurs at some energetic cost to the animal (Williams, 2017). In favor of this, hypothesis is the fact that synthesis of porphyrins and bile pigments has been shown to be energetically costly in other taxa (e.g., in bird egg shell pigments Miksik, Holáň, & Deyl, 1994; Morales, Velando, & Torres, 2010; Moreno & Osorno, 2003; Soler, Navarro, Contreras, Avilés, & Cuervo, 2008).

Further evidence for the de novo synthesis of porphyrin pigments in mollusks comes from studies on marine pearl oyster shells. Porphyrins have been found in oyster shells and pearls and are thought to contribute to visible pigmentation in some species (Fischer & Haarer, 1932; Kosaki, 1947; Miyoshi, Matsuda, & Komatsu, 1987; Nicholas & Comfort, 1949; Tixier, 1945), and heritability of shell and pearl color has been confirmed by breeding experiments (Ky et al., 2013, 2015; Lemer et al., 2015). De novo synthesis of shell pigments is confirmed by aquaculture techniques, where mantle tissue from one animal is grafted into another to produce pearls that match the color of those obtained from the donor animal (Ky et al., 2013, 2015). These even include xenografts between species, where black pearls were produced by placing mantle tissue of *Pinctada margaritifera* in gonad tissue of *Pinctada maxima* (McGinty, Zenger, Jones, & Jerry, 2012). Black shell coloration in *P. margaritifera* is due to porphyrins, but porphyrins are not found in *P. maxima* (Miyoshi et al., 1987), which does not normally produce black pearls (McGinty et al., 2012).

### 4.3 Color pattern

The production of color patterns has been the focus of many computational and modeling studies that have sought to find a mechanism for the production of shell patterns (e.g., Meinhardt, 1995). Based on these ideas, we can hypothesize on how color patterns are formed in *Clanculus* species. Three pigments are responsible for the dominant colors in the two *Clanculus* species studied: trochopuniceus for the pink‐red, trochoxouthos for yellow‐brown, and eumelanin for black (Williams et al., 2016). In both species, trochopuniceus is produced in spots in early whorls. The color pattern in later whorls of *C. pharaonius* shells would suggest that some mantle cells are producing only eumelanin and others only trochopuniceus. The production of eumelanin is switched on and off producing black and white spots that are congruent with small granules in the shell sculpture. The production of trochopuniceus is almost continuous. On the other hand, in *C. margaritarius*, in later whorls, all mantle cells alternate between production of eumelanin and trochoxouthos. Shell patterns suggest that pigment‐producing cells pause in pigment production when shifting from one pigment to the other, with a greater break in pigment production after the production of eumelanin, creating unevenly sized patches of white on either side of the black spots.

## 5 CONCLUSIONS

Our work advances studies on the evolution of shell color in mollusks by matching known shell pigments with the identification of the genetic pathway responsible for their biosynthesis. We identify genes associated with the production of porphyrin pigments in colored foot tissue and mantle tissue in two species known to have porphyrin pigmentation and one that does not. Relative expression data based on qPCR are consistent with the evidence of taxonomic distribution of porphyrin as determined in recent biochemical studies and with the hypothesis that porphyrin‐based pigments uroporphyrin I and uroporphyrin III are synthesized de novo by two *Clanculus* species but not by *Ca. zizyphinum*. These results are relevant not only to understanding the evolution of shell pigmentation in *Clanculus* but also to understanding the evolution of color in other species with uroporphyrin pigmentation, including (mainly marine) mollusks, annelid and platyhelminth worms, and turaco bird feathers.

We recommend the use of species such as *C. margaritarius* and *C. pharaonius* that share the same pigments in the foot and shell as an aid to identify the genes involved in pigment production. Although a foot that shares the same color and pattern as the shell is unusual, occurring in only a few molluskan groups, where it does occur, it provides an almost unique opportunity to identify the genes involved in the inheritance and control of color. In such taxa, it becomes possible to distinguish between genes that are involved in pigmentation, and those involved in biomineralization as the latter will not be expressed in the foot. This may be particularly useful in identification of novel proteins that may be associated with bilins or carotenoids.

## CONFLICT OF INTEREST

The authors have declared that no competing interests exist.

## AUTHOR CONTRIBUTIONS

STW conceived the project, produced the final figures (other than Figure 4, produced by AEL), and wrote the first draft of the manuscript and STW, DIS, and AEL edited final drafts. AEL undertook qPCR studies and PD and TN contributed to other molecular laboratory work. AEL, DIS, STW, and CKCC analyzed molecular data and TN provided live‐collected specimens and photographs of living specimens.

## DATA ACCESSIBILITY

Details of specimens are available in Table 1. Voucher specimens have been deposited in the Natural History Museum, London (registration numbers in Table 1). Transcriptome assembly statistics are available in Table 2. Alignments of haem sequences are available in Appendix S1. Photographs of shells corresponding to divergent lineages within *Clanculus margaritarius* are available in Appendix S2. Open‐reading frames for all haem transcripts are available in Appendix S3. Normalized qPCR results and probabilities for *t* tests are given in Table 6 and Appendix S4. Raw reads for all transcriptomes have been published online in the NCBI Sequence read Archive: NCBI‐SRA SRP092238, SRP092881, and SRP092239.
