# Supplementary figures and images for: Colorful seashells: Identification of haem pathway genes associated with the synthesis of porphyrin shell color in marine snails
Source: Ecol Evol. 2017 Oct 30;7(23):10379–97. doi: 10.1002/ece3.3552 (PMC5723588; doi:10.1002/ece3.3552)

## ALAS alignment

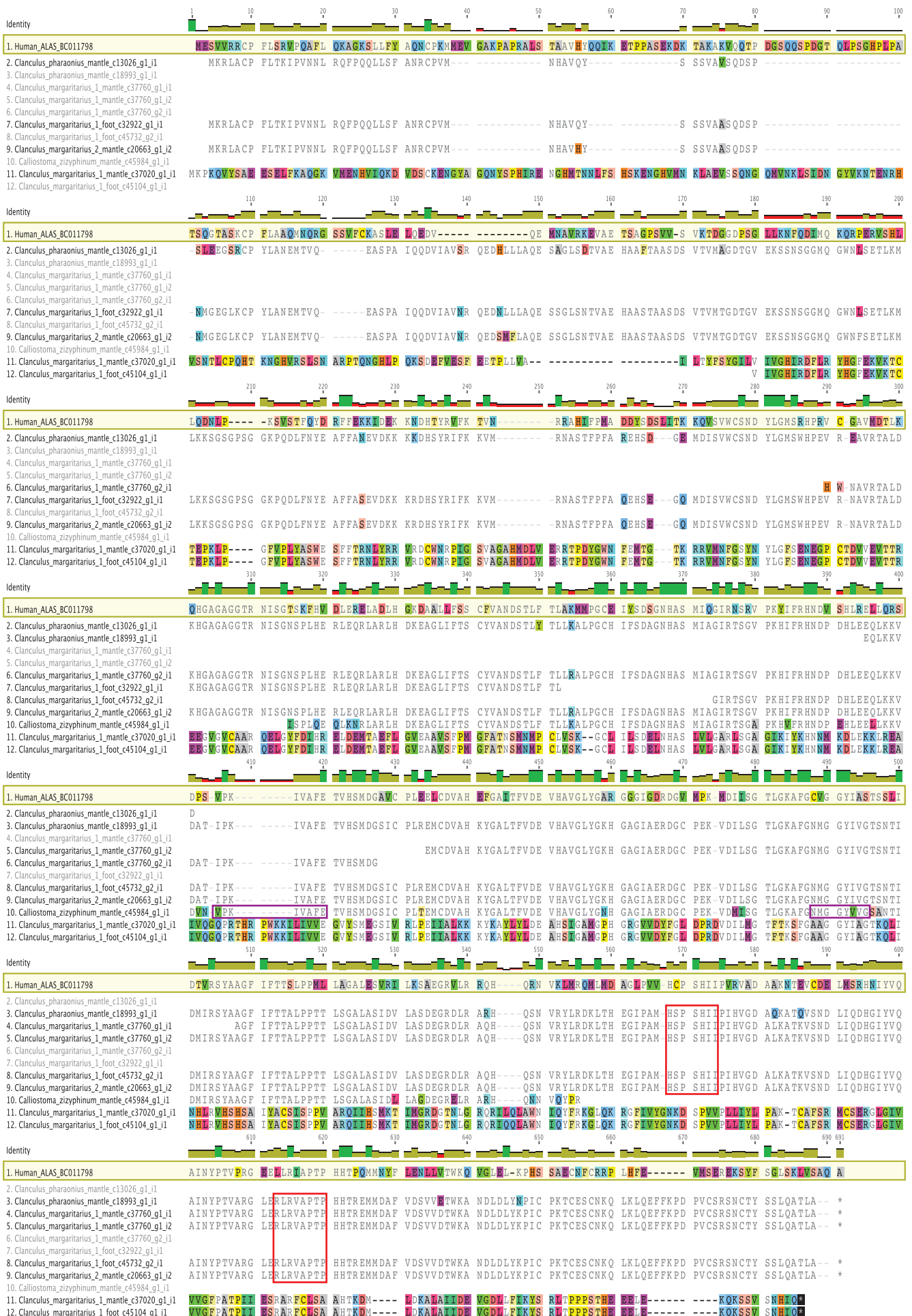

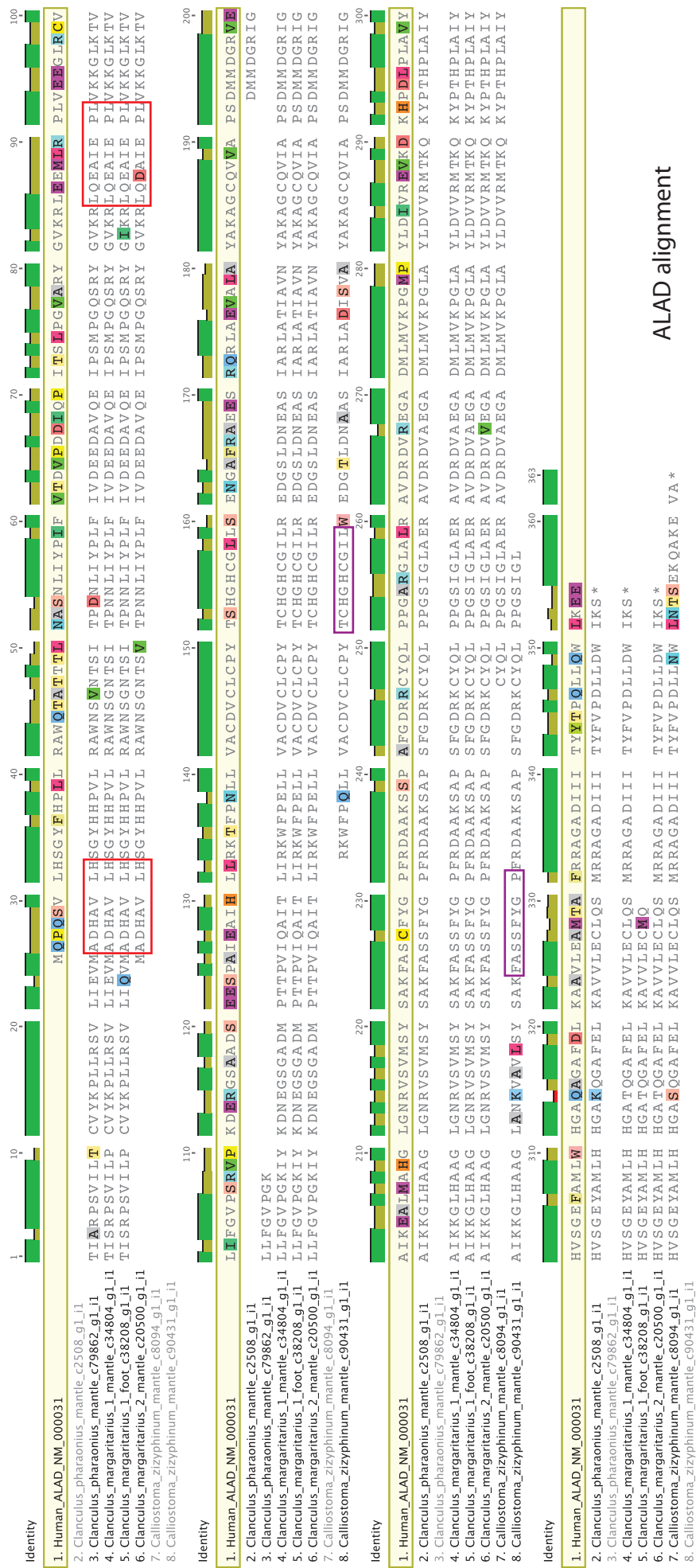

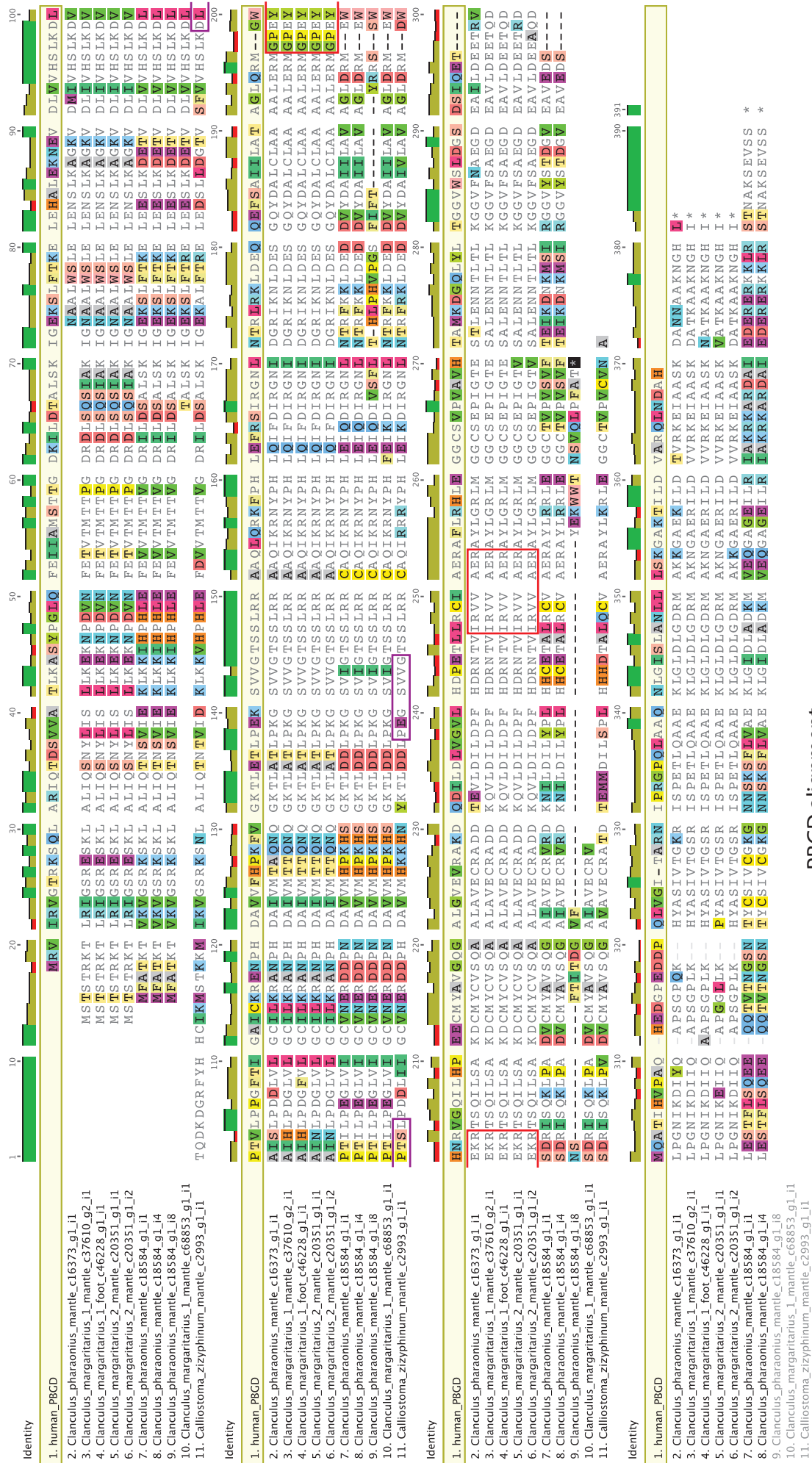

## PBGD alignment

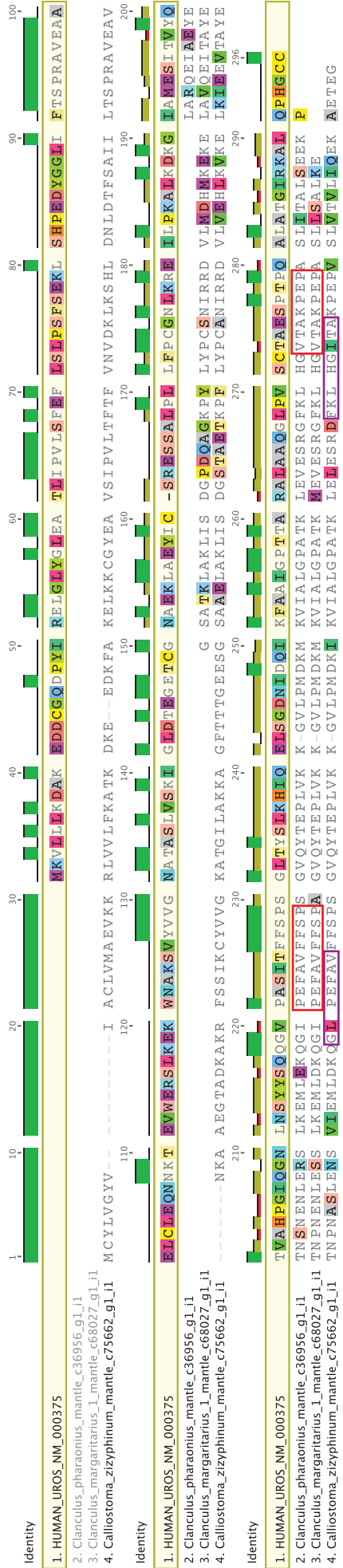

UROS alignment

## UROD alignment

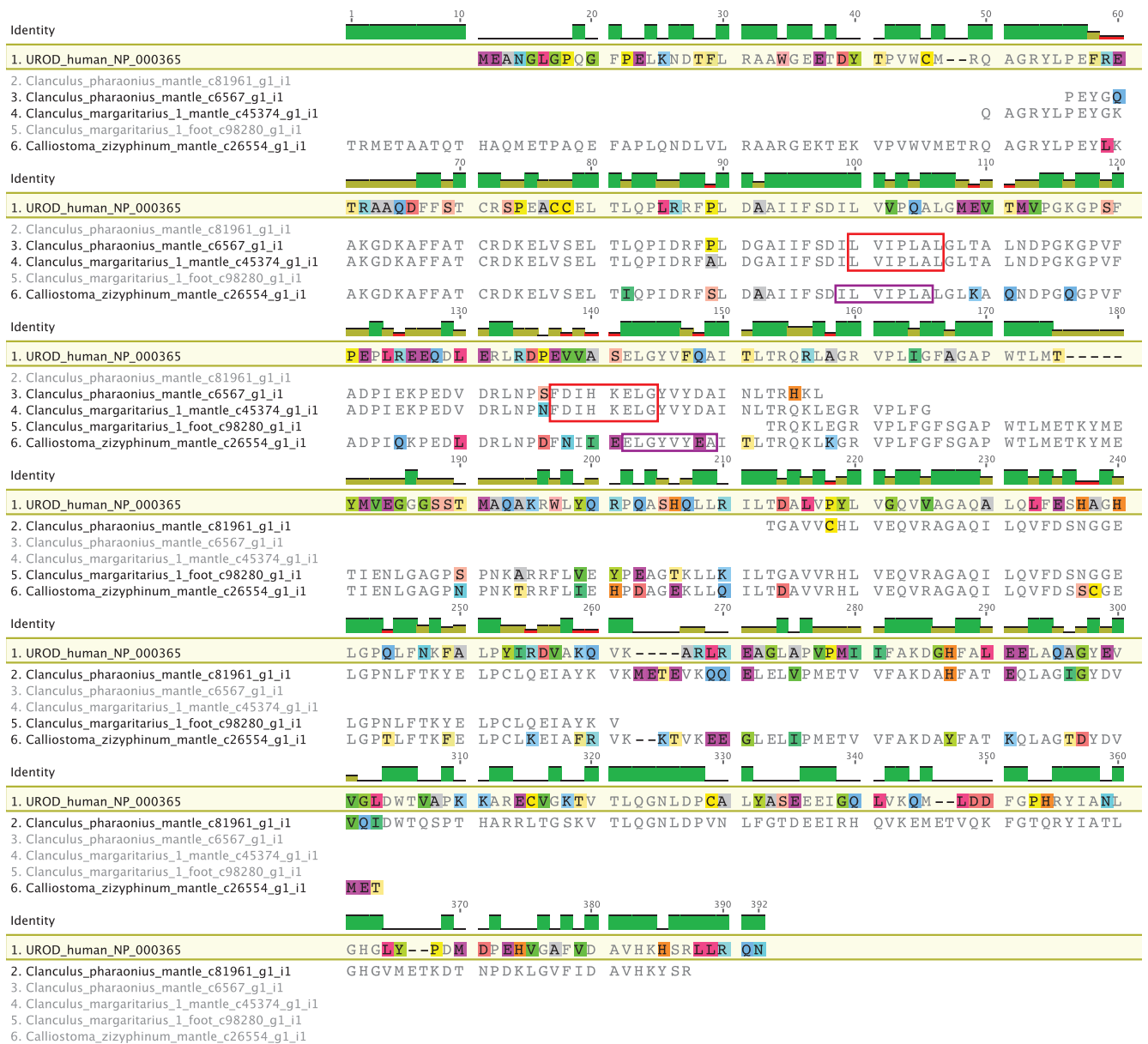

Supplement: Supplementary file 1 [file ECE3-7-10379-s001.pdf]

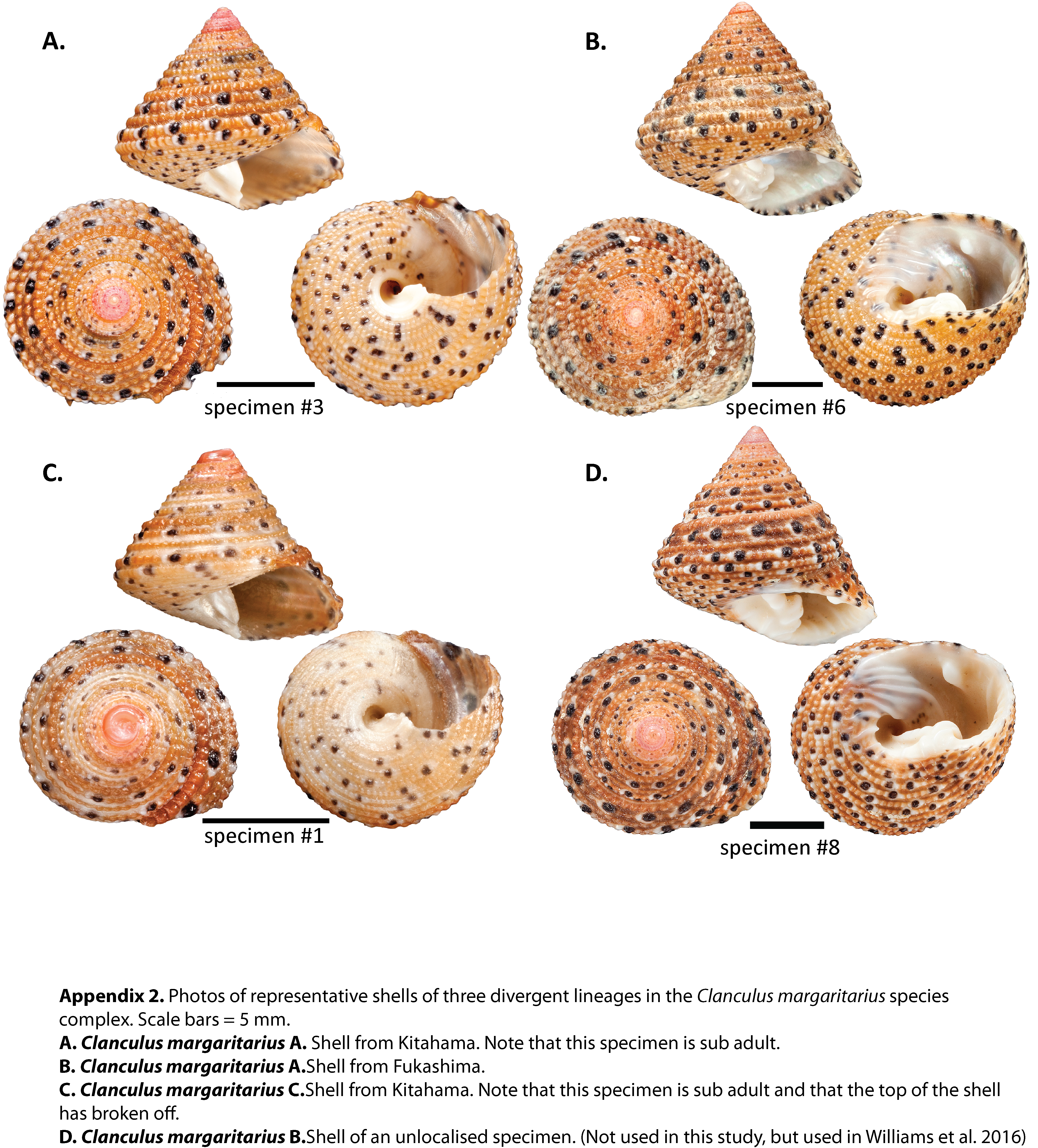

Supplement: Supplementary file 2 [file ECE3-7-10379-s002.tif]
