# Supplementary material for: Colorful seashells: Identification of haem pathway genes associated with the synthesis of porphyrin shell color in marine snails
Source: Ecol Evol. 2017 Oct 30;7(23):10379–97. doi: 10.1002/ece3.3552 (PMC5723588; doi:10.1002/ece3.3552)
Supplement: Supplementary file 3 [file ECE3-7-10379-s003.pdf]

>Clanculus_margaritarius_1_mantle_c34804_g1_i1 m.4035 c34804_g1_i1|g.4035 ORF
c34804_g1_i1|g.4035 c34804_g1_i1|m.4035 type:5prime_partial len:354 (+)
c34804_g1_i1:3-1064(+)
GCACGATATCGAGACCCAGCGTCATATTGCCGTGTGTTTACAAACCCTTGCTTAGGTCAGTCCTGATTGAAGTCATGGCG
GATCACGCAGTTCTTCACAGTGGCTATCACCATCCGGTTCTACGGGCGTGGAATTCAGGCAACACTTCAATAACGCCAAA
CAATCTCATATACCCGCTATTTATCGTTGACGAGGAGGATGCAGTCCAAGAAATACCCAGCATGCCTGGCCAGTCCAGAT
ATGGCGTCAAGCGTCTACAAGAAGCCATTGAACCACTTGTTAAGAAAGGGCTGAAAACTGTACTCCTTTTTGGTGTTCCT
GGAAAAATTTATAAGGACAATGAAGGCAGTGGTGCTGACATGCCAACAACTCCAGTTATACAAGCCATCACATTAATACG
CAAGTGGTTCCCGGAACTGTTAGTAGCGTGTGATGTGTGTCTGTGTCCTTACACCTGTCACGGACACTGTGGTATTCTAA
GAGAAGATGGCTCGTTAGATAACGAGGCGAGTATTGCCCGACTTGCAACTATTGCAGTCAACTATGCCAAAGCAGGCTGT
CAGGTAATCGCCCCTTCTGACATGATGGACGGGAGGATCGGGGCAATAAAGAAAGGTCTCCATGCGGCAGGGCTTGGTAA
CAGAGTCAGTGTAATGAGCTACAGTGCCAAGTTTGCATCTAGTTTCTATGGACCTTTCCGTGATGCAGCTAAAAGTGCCC
CATCATTCGGCGATAGGAAGTGTTACCAACTGCCGCCTGGATCTATTGGTCTCGCAGAGAGGGCAGTGGACCGAGACGTG
GCTGAAGGGGCAGACATGTTGATGGTGAAACCTGGCCTTGCATACTTAGATGTCGTCAGAATGACAAAACAAAAATACCC
AACCCACCCGCTAGCAATCTATCACGTATCTGGGGAGTACGCCATGTTGCATCATGGCGCCACGCAGGGAGCGTTTGAGT
TGAAGGCAGTGGTACTGGAGTGTCTGCAGAGTATGAGACGGGCAGGTGCTGATATAATTATCACCTACTTCGTTCCTGAT
CTCCTGGACTGGATTAAGTCATAGACTATCAGTGTCAGTTGTCATTGCACTTGAAAGTTGTCTACATTTTGGTCATTGGA
AATATTACACATGGGCAGAACAAACTACCCTTTTGTGTGCTGAATTAAACTTTTGTAAGGGCTTTATGCCATCCTGTTAT
GAATTAACTTGTAATCAGCTCATTTAACCCTTAAACTGTGGGGCCTTCTTCTAATGATCTCTGATCATGTAGAGGTCATC
TTGCCACAGTTAAAGGATGAACAAGTGTCTCCATTTCCAAATTCATTACATTAGGCCT
>Clanculus_margaritarius_1_mantle_c37020_g1_i1 m.1709 c37020_g1_i1|g.1709 ORF
c37020_g1_i1|g.1709 c37020_g1_i1|m.1709 type:complete len:641 (-)
c37020_g1_i1:38-1960(-)
AGGCTGACATTACTATCACTGTACCAATGAACTATATGAAAGTCGGGCTTCACATCTCTTCATAGCAATGGCGTATTATT
GACTCCTGGGAACTACCTGTGATATGGCCGCTAGGTGGTTGGGATATTACTGATGAAGGACTTGTGCGATAAGAATTATA
ACTAACTATGAAGCCGAAGCAAGTCTATTCAGCGGAAGAAAGCGAACTATTTAAAGCTCAAGGAAAAGTGATGGAAAACC
ACGTTATTCAAAAGGACGTCGACAGCTGCAAAGAAAATGGATACGCTGGTCAGAACTACTCGCCACACATTAGGGAGAAC
GGACATATGACAAATAATCTTTTCTCACATTCTAAAGAGAATGGACACGTGATGAACAAGCTCGCTGAAGTGTCATCACA
AAATGGACAAATGGTCAATAAACTCTCCATAGACAATGGATATGTGAAAAACACGGAAAATAGACATGTATCAAATACTT
TGTGTCCTCAGCATACAAAGAATGGACACGTCAGAAGCTTATCTAATGCAAGGCCAACACAGAATGGACATTTGCCTCAG
AAGAGTGATGAGTTTGTTGAATCATTTGAAGAAACGCCTCTGCTGGTGGCAATTCTCACCTACTTCAGCTACGGAATACT
CGTGATAGTTGGCCACATTAGGGACTTTCTCCGTTACCATGGCTTTGAAAAAGTGAAAACATGCACAGAACCAAAACTTC
CGGGCTTTGTCCCGCTGTACGCTTCATGGGAAAGCTTCTTCACGCGGAACTTGTACAGACGGGTGCGGGATTGCTGGAAC
CGGCCTATTGGCAGTGTAGCGGGGGCACATATGGATCTCGTGGAGCGACGGACGCCAGACTATGGATGGAACTTTGAAAT
GACGGGTACAAAGAGACGAGTGATGAACTTCGGATCCTACAACTACCTGGGTTTCTCCGAGAATGAGGGGCCGTGTACTG
ACGTCGTTGAAGTTACCACGAGGGAGGAAGGGGTCGGGGTGTGCGCCGCCAGGCAGGAGCTTGGCTACTTTGATATTCAC
CGAGAACTGGATGAAATGACTGCAGAGTTTCTAGGCGTGGAGGCTGCAGTTTCCTTCCCAATGGGCTTTGCTACCAACTC
CATGAACATGCCGTGTCTTGTCAGTAAGGGTTGTTTGATTTTGAGTGATGAGTTGAACCACGCATCACTCGTATTAGGAG
CTCGCCTTTCTGGAGCCGGTATCAAGATCTATAAACATAACAATATGAAAGATTTAGAGAAGAAATTACGCGAGGCAATA
GTTCAAGGGCAGCCACGCACACATCGGCCCTGGAAGAAAATACTCATCGTGGTGGAGGGTGTGTACAGTATGGAAGGCTC
TATCGTCCGTCTCCCCGAGATAATCGCTTTAAAGAAGAAGTACAAAGCTTACTTGTACCTGGATGAAGCCCACAGTATAG
GAGCCATGGGGCCACATGGGCGTGGAGTTGTAGACTACTTCGGGCTGGATCCTCGAGATGTCGATATTCTCATGGGGACA
TTCACAAAGAGCTTCGGGGCAGCTGGGGGATACATCGCCGGGACGAAGCAACTGATCAATCATCTACGAGTCCATTCACA
CAGTGCCATCTATGCATGTTCAATATCGCCACCAGTAGCACGACAGATCATACATTCCATGAAGACCATCATGGGTCGAG
ACGGAACCAATCTAGGCCGACAGAGGATCCTGCAGCTGGCGTGGAATATCCAGTATTTCCGGAAGGGCTTACAGAAGAGG
GGATTCATCGTGTATGGCAACAAGGATTCTCCAGTGGTTCCTTTGCTAATATATTTACCTGCTAAAACATGTGCGTTTAG
CAGGATGTGCAGTGAGCGAGGACTAGGCATTGTGGTGGTTGGTTTTCCAGCAACCCCTATAATTGAGTCTAGAGCTCGCT
TCTGTCTATCAGCCGCCCACACCAAGGATATGCTCGACAAGGCATTGGCTATTATAGACGAGGTAGGAGATTTGTTGTTC
ATCAAGTATTCACGGCTCACACCACCACCTTCCACCCACGAGGAGGAGTTAGAGAAACAAAAATCTTCAGTGTCAAATCA
TATACAGTGATGTGATCTATTTCAAGAAGAAGCTATAGTGGTGCTAC
>Clanculus_margaritarius_1_mantle_c37610_g2_i1 m.3363 c37610_g2_i1|g.3363 ORF
c37610_g2_i1|g.3363 c37610_g2_i1|m.3363 type:complete len:367 (+)
c37610_g2_i1:93-1193(+)
TCACTGAGGTGAAGAACAGTAATTCTGTGGAAGTCCCTCAATTTCACCCTGCGCTTCCAAGCCGTCAGCACTTGTGAAAT
TTCTCCATAAGCATGTCTACCTCAACAAGGAAAACTCTTAGGATCGGGTCAAGAGAAAGCAAGCTCGCTTTGATCCAGTC
CAACTATTTGATATCCTTGTTAAAGGAGAAAAACCCGGATGTCAACTTTGAAACGGTTACCATGACGACACCCGGCGACA
GAGATCTAAGCCAATCAATCGCGAAGATCGGTAACGCAGCCCTTTGGTCACTTGAGCTGGAGAACTCTCTGAAGGCCGGT
AAAGTGGACTTGATTGTCCACTCTTTGAAGGACGTAGCTATACACCTACCTGATGGTCTCGTCCTAGGATGTATACTTAA
GCGAGCAAACCCACACGATGCCATTGTCATGACAACACAAAATCAGGGGAAAACTTTAGCAACGTTGCCAAAAGGAAGTG
TTGTAGGCACCAGTTCGTTACGAAGGGCGGCACAGATCAAAAGGAATTACCCTCACCTACAGATATTCGACATCCGAGGT

AATATTGATGGTCGGATTAAGAATCTGGATGAGAGCGGGCAGTATGACGCACTGTGTCTAGCTGCCGCCGCACTCGAAAG
AATGGGCCCCGAATATGAGAAGAGAACATCACAGATCCTGTCTGCTAAAGACTGTATGTACTGTGTGAGTCAAGCGGCCC
TGGCGGTGGAGTGTCGAGCAGACGACAAGCAGGTTCTGGACATCCTGGATCCTTTCCATGACAGGAACACCGTCATCAGA
GTCGTCGCCGAGAGGGCGTACCTTGGGAGGCTGATGGGCGGATGCAGCGAGCCTATAGGGACCGAGTCGGCCCTGGAGAA
CAACACTCTGACACTGAAGGGAGGCGTGTTTAGCGCCGAGGGAGACGAGGCAGTCCTGGACGAAGAGACGCAAGACCTGC
CGGGAAATATCAAAGATATCATTCAGGCACCAAGTGGACCTCTCAAGCACTACGCGTCAATAGTGACGGGTAGTAGAATC
AGTCCAGAAACTCTGCAGGCTGCGGAGAAATTGGGATTGGATCTCGGAGACAGGATGGCCAAGAATGGGGCAGAGAGGAT
ACTGGACGTTGTCCGAAAGGAGATCGCAGCGTCCAAAGATGCCACTAAAGCAGCAAAGAATGGACATATCTGACTTAAAC
ATGTCTAAGCGTACCGACCCTGTTAGCCGGAGATTTTATATTAGTTCAGGATATTTTTTATTTTTGTTTTGTTGCCTGTA
ATGAAACATTATGGGTAATCGTTTTCAAGAAATTTACCATATACAACATCAGAAGGATTTTACAGCTTGTTTAGGGTTTT
GGTTTTAGATCCACAAAATCGGTTTTATGAAGAGACCGTAAGTGATATTTGAAACACTGTCCTGAGAGTTGGTATTATCA
TAGGTTTAAGACAATCTAATGCTGAGATCCTTTTATGAAACAG
>Clanculus_margaritarius_1_mantle_c37760_g1_i1 m.4903 c37760_g1_i1|g.4903 ORF c37760_g1_i1|g.4903 c37760_g1_i1|m.4903 type:5prime_partial len:177 (+) c37760_g1_i1:1-531(+)
GCTGGGTTCATCTTTACAACAGCTCTACCCCCAACAACACTAAGTGGAGCCCTGGCATCCATTGATGTGTTGGCGAGTGA
TGAAGGCCGAGATCTCCGTGCCCAACATCAGAGTAACGTCCGCTACCTACGAGACAAGTTGACTCATGAAGGAATTCCGG
CCATGCACAGCCCCAGTCACATCATACCAATACACGTTGGCGATGCCCTCAAAGCTACCAAGGTGTCGAACGATCTTATC
CAGGACCATGGCATTTACGTCCAGGCCATCAACTATCCGACAGTCGCTCGTGGACTGGAAAGACTTCGTGTGGCCCCAAC
TCCTCACCATACCCGGGAAATGATGGACGCCTTCGTTGACAGCGTAGTCGACACCTGGAAGGCAAATGACCTTGACCTTT
ACAAGCCGATCTGTCCAAAGACATGTGAGAGCTGCAACAAACAACTCAAGCTCCAAGAGTTCTTCAAGCCTGATCCAGTT
TGTTCCCGCTCTAACTGTACATACTCCTCCCTTCAGGCGACTCTTGCATAAGGGCAACCTTGCATGTCACATGTTCAGTT
TGTGACCTTGACCTTAGTCCTGTAGCGACCAGGCTTTTATATTTGTGATTGAAATATATTCTCCTACTGACTTACTTAAC
CATAAACCCTTTGGCAGACAGTTTATTTTCAGTCATTCCCAATAATATCTGTGGTCACTATGTCGATTAAACAGTTAGTC
ACCATGATACTGATAATCACTAGATTCAAAATCTTAATTAGAACACTTTGTTGTGAATTATTTCTGCATATTGAAAAGAA
ATTTAATTTATGATGCGAAAATTTAACAATATATTAGAAGTGACTGGTTATTAACCAGGATGAATTTATTTATCTACATA
CAAGTGTTTTGTGCAGAACTTGGAACCAGGTTTGAATTGGTCTTATTCAATATTTGGTGTATATTATCGTCTCCAATAAA
TGGGTATATAAGTGTGTTCATTTCATACAAATGTTTTATAATAAACATGTGCAATTATTAGTCATACTTTGTCTCATTAA
AATTTCAATTTTTAATAAAATTTTTATTATTATCTGTCAGGTTTCAAAACCATTAAAGTTTTGTGCTAAGAAAGATGACC
TTAAGAGGTATAACTATAAGTGTATCATTAAATAATTGTTTGTAAAGTGGTTCAAC
>Clanculus_margaritarius_1_mantle_c37760_g1_i2 m.3737 c37760_g1_i2|g.3737 ORF c37760_g1_i2|g.3737 c37760_g1_i2|m.3737 type:5prime_partial len:250 (+) c37760_g1_i2:1-750(+)
GAGATGTGTGACGTGGCCCACAAGTACGGTGCCTTAACCTTCGTGGATGAAGTCCATGCTGTGGGGCTGTACGGCAAACA
TGGAGCTGGCATTGCTGAAAGAGATGGATGTCCAGAAAAAGTTGACATTCTGTCAGGAACTCTTGGAAAGGCATTTGGCA
ATATGGGAGGCTACATAGTTGGGACGTCCAATACTATTGACATGATACGGAGCTATGCTGCTGGGTTCATCTTTACAACA
GCTCTACCCCCAACAACACTAAGTGGAGCCCTGGCATCCATTGATGTGTTGGCGAGTGATGAAGGCCGAGATCTCCGTGC
CCAACATCAGAGTAACGTCCGCTACCTACGAGACAAGTTGACTCATGAAGGAATTCCGGCCATGCACAGCCCCAGTCACA
TCATACCAATACACGTTGGCGATGCCCTCAAAGCTACCAAGGTGTCGAACGATCTTATCCAGGACCATGGCATTTACGTC
CAGGCCATCAACTATCCGACAGTCGCTCGTGGACTGGAAAGACTTCGTGTGGCCCCAACTCCTCACCATACCCGGGAAAT
GATGGACGCCTTCGTTGACAGCGTAGTCGACACCTGGAAGGCAAATGACCTTGACCTTTACAAGCCGATCTGTCCAAAGA
CATGTGAGAGCTGCAACAAACAACTCAAGCTCCAAGAGTTCTTCAAGCCTGATCCAGTTTGTTCCCGCTCTAACTGTACA
TACTCCTCCCTTCAGGCGACTCTTGCATAAGGGCAACCTTGCATGTCACGTGTTCAGTTTGTGAACTTGACCTTAGTCCT
GTAGCGATCAGGCTTTTATATTTGTGATGGAAAAATATTCTCCTACTGACTTACTTAACCAAAAACTCTTCGGCAGATAG
TTTATTTTCAGTCATTCCCAATGATATCTGTGGTCACTATGTTGATTAAACAGTTAGTCACCATGATACTAAAAATCACT
AGATTCAAAATCTTAATTAGAACACTTTGTTGTGAATTATTTCTGCATATTGAAAAGAAATTTAATTTATGATGCAAAAA
TTGAAGAATATATTAGTAGTAATTGGTTAATAAATGAATTCATTTATCTACATACAAGTGTTTTGTGCAGAACTTGGAAC
CAGGTTTGAATTGGTCTTTAAAGTTCAAATTATTCAATATTTGGTGTATATTATCGTCTCCAATAAATGGGTATATAAGT
GTGTTCATTTCATACAAATGTTTTATAATAAACATGTGCAATTATTAGTCATTTTGTCTCATTACAATTTCAATTTTTAA
TCGAATTTTTATTATTATCTGTCAGGTTTCAAAACCATTAAAGTTTTGTGCTAAGAAAGATGACCTTAAGAGGTATAACT
ATAAGTGTATCATTAAATAATTGTTTGTAAAGTGGTTCAAC
>Clanculus_margaritarius_1_mantle_c37760_g2_i1 m.23250 c37760_g2_i1|g.23250 ORF c37760_g2_i1|g.23250 c37760_g2_i1|m.23250 type:internal len:129 (+) c37760_g2_i1:3-386(+)
AGCACTGGAACGCTGTAAGGACCGCACTGGACAAGCACGGGGCTGGTGCAGGTGGAACAAGGAACATCTCGGGGAACTCG
CCACTCCATGAACGTCTCGAACAACGTCTTGCTCGTCTGCACGACAAGGAGGCTGGCCTCATCTTCACCTCATGCTATGT
TGCCAATGACTCCACTCTGTTTACCTTACTCAGAGCACTTCCAGGTTGCCATATTTTCTCGGACGCGGGTAACCATGCCT
CCATGATTGCTGGTATCAGAACAAGTGGGGTACCGAAGCACATCTTCCGTCACAATGACCCCGATCACCTTGAGGAGCAG
CTCAAGAAGGTTGATGCCACCATCCCCAAAATAGTTGCCTTTGAGACAGTACATTCCATGGATGGATC

>Clanculus_margaritarius_1_mantle_c45374_g1_i1 m.25992 c45374_g1_i1|g.25992 ORF c45374_g1_i1|g.25992 c45374_g1_i1|m.25992 type:internal len:117 (+) c45374_g1_i1:2-349(+)
ACAGGCCGGCAGATATCTGCCAGAATATGGTAAAGCAAAAGGTGATAAGGCTTTCTTTGCTACGTGTCGAGACAAAGAAC
TTGTCAGTGAACTTACATTGCAGCCTATTGACAGGTTTGCCCTTGATGGTGCTATCATCTTCTCCGACATATTGGTTATC
CCCCTTGCCTTGGGGTTGACAGCGCTGAATGATCCAGGAAAGGGTCCTGTGTTTGCCGATCCCATCGAGAAACCAGAAGA
CGTTGATCGCCTGAATCCCAACTTTGATATTCACAAGGAGCTGGGTTATGTTTATGATGCCATAAATCTCACACGACAAA
AACTCGAAGGAAGAGTGCCGTTATTTGGA
>Clanculus_margaritarius_1_mantle_c68027_g1_i1 m.21413 c68027_g1_i1|g.21413 ORF c68027_g1_i1|g.21413 c68027_g1_i1|m.21413 type:internal len:139 (-) c68027_g1_i1:3-416(-)
CAGGCAGTGCAACAAAACTGGCGAAACTAATCTCTGATGGTCCCGACCAGGCAGGGAAACCATACTTGTACCCTTGCTCC
AACATCAGACGTGACGTTCTCATGGACCACATGAAGGAGAAAGAACTGGCAGTACAGGAAATTACTGCATATGAGACCAA
CCCAAATGAGAATCTGGAAAGCAGCTTAAAGGAAATGCTGGATAAGCAGGGCATTCCTGAATTTGCAGTGTTCTTCAGTC
CTGCTGGCGTACAATACACAGAACCACTTGTGAAGAAAGGTGTACTGCCCATGGATAAAATGAAGGTGATAGCTTTGGGT
CCTGCTACTAAGATGGAGGTGGAGTCCCGAGGGTTCAAGTTACACGGTGTTACAGCCAAACCAGAACCAGCCAGCCTTCT
CAGCGCCCTCAAGGAGTA
>Clanculus_margaritarius_1_mantle_c68853_g1_i1 m.17681 c68853_g1_i1|g.17681 ORF c68853_g1_i1|g.17681 c68853_g1_i1|m.17681 type:internal len:162 (+) c68853_g1_i1:1-483(+)
ACAGCATTATCTAAGATCGGTGAAAAGTCACTATTCACGAGGGAACTAGAGGAATCTCTGAAGGACGAAACAGTTGATCT
TGTGGTGCACTCACTGAAAGATTTACCCACAATTCTTCCTGAAGGACTTGTCATAGGTTGTGTGAATGAGCGTGATGATC
CCAATGATGCAGTTGTTATGCATCCAAAACACTCGGGGAAAACTTTGGATGATCTGCCAAAAGGGAGTGTGATTGGTACC
AGTTCCTTAAGGCGATGTGCACAGATAAAGAGAAACTATCCACATTTTGAAATCAAAGATATTAGGGGTAATCTGAATAC
ACGGTTCAAGAAACTTGATGAGGATGATGTTTATGATGCTATAATATTAGCTGTGGCTGGTCTAGATCGAATGGAATGGT
CTGATCGAATATCTCAGAAGTTGCCTGCTGATGTGTGTATGTATGCTGTGAGCCAGGGGGCTATAGCAGTAGAGTGCCGA
GTGAG
>Clanculus_margaritarius_1_mantle_c87276_g1_i1 m.12857 c87276_g1_i1|g.12857 ORF c87276_g1_i1|g.12857 c87276_g1_i1|m.12857 type:5prime_partial len:151 (-) c87276_g1_i1:164-616(-)
TGGTCACAGTCATGCCTACAGACTGGTCTGGCAGTCCAAGGTTGGTCCACTGCCCTGGTTGAGCCCACAGACAGATGATG
CTATCAAGGGTCTGGTTGCGAGAGGTCGCAAGAATCTACTTCTCGTGCCGATAGCCTTCACCAGCGACCATATTGAAACT
TTGTTTGAGTTGGACTACGAGTATGCACAGAATCTTGGGGCAGAGGTCGGTGTCAAGAACATCAGACGGGCAGCATCTCT
GAATGACAACCCTGTCTTCATTGAGGCCCTGGCCGACCTGGTAAAGACACATCTACACACCCAACAGGTGTGCACACCAC
AGCTCCTTCTCCGTTGTCCGATGTGCACCAACCCGGTGTGCGGTCTCGCCAAAGAGTTCTTCCGTGATCAGCAGCCCATC
CTTGACACTCTACGAGCTGAGGACGAGGAGCTGAAACTAGGGGCCAAGTCTTGATGTACTTTGAGTCTTCTGCAATCTGG
TTAAAATCATTGTTTTGATATGCAGGGTCTGATTTTCAGGAAATGAATTTTGATTCAAAGACTAACATTAACCCTATGGT
TACTGAATTGAATTTAAGTTTGACCAAACAATACCTGTGATTGGCACTCTGAGGGTT
>Clanculus_margaritarius_1_foot_c32922_g1_i1 m.8395 c32922_g1_i1|g.8395 ORF c32922_g1_i1|g.8395 c32922_g1_i1|m.8395 type:3prime_partial len:285 (-) c32922_g1_i1:2-853(-)
GCAAGCACCTGTTTCCAGCTGTGAGAACAATTAATATACTCGGCAACCATGAAGCGACTTGCATGTCCATTTCTCACCAA
GATACCAGTCAACAACTTGAGGCAGTTTCCTCAGCAGTTGCTGTCCTTTGCCAACCGATGTCCAGTGATGAACCACGCCG
TTCAGTACTCGTCCAGTGTTGCTGCCAGCCAGGACAGTCCCAACATGGGAGAGGGGTTGAAGTGTCCGTACCTGGCCAAT
GAGATGACAGTCCAAGAAGCCAGTCCAGCCATTCAACAAGATGTCATTGCTGTTAATAGACAAGAGGACAATCTGCTTCT
TGCCCAGGAGAGCTCTGGTCTCAGCAACACTGTGGCAGAACATGCAGCGTCCACCGCCGCCTCGGACAGTGTGACAGTCA
TGACAGGAGATACTGGGGTGGAGAAGAGCTCCAACTCAGGGGGGATGCAGGGCTGGAACTTGAGTGAGACCCTCAAGATG
CTGAAGAAATCTGGATCCGGCCCCAGTGGAGGAAAACCTCAGGATCTGTTCAACTATGAAGCCTTCTTTGCAAGTGAAGT
GGACAAGAAGAAAAGAGACCATTCCTATCGTATTTTCAAGAAAGTGATGCGCAATGCAAGTACCTTTCCTTTTGCTCAGG
AACATTCAGAAGGCCAGATGGACATCTCGGTGTGGTGTAGTAACGACTATCTGGGGATGAGCTGGCATCCAGAAGTCAGG
AACGCTGTAAGGACCGCACTGGACAAGCACGGGGCTGGTGCAGGTGGAACAAGGAACATCTCGGGGAACTCGCCACTCCA
TGAACGTCTCGAACAACGTCTTGCTCGTCTGCACGACAAGGAGGCTGGCCTCATCTTCACCTCATGCTATGTTGCCAATG
ACTCCACTCTGTTTACCTTAC
>Clanculus_margaritarius_1_foot_c38208_g1_i1 m.7342 c38208_g1_i1|g.7342 ORF c38208_g1_i1|g.7342 c38208_g1_i1|m.7342 type:internal len:330 (+) c38208_g1_i1:3-989(+)
GCACGATATCGAGACCCAGCGTCATATTGCCGTGTGTTTACAAACCCTTGCTTAGGTCAGTCCTGATTCAAGTCATGGCG
GATCACGCAGTTCTTCACAGTGGCTATCACCATCCGGTTCTACGGGCGTGGAATTCAGGCAACACTTCAATAACGCCAAA
CAATCTCATATACCCGCTATTTATCGTTGACGAGGAGGATGCAGTCCAAGAAATACCCAGCATGCCTGGCCAGTCCAGAT
ATGGCATCAAGCGTCTACAAGAAGCCATTGAACCACTTGTTAAGAAAGGGTTGAAAACTGTACTCCTTTTTGGTGTTCCT

GGAAAAATTTATAAGGACAATGAAGGCAGTGGTGCTGACATGCCAACAACTCCAGTTATACAAGCCATCACATTAATACG
CAAGTGGTTCCCGGAACTGTTGGTAGCGTGTGATGTGTGTCTGTGTCCTTACACCTGTCACGGACACTGTGGTATTCTAA
GAGAAGATGGCTCGTTAGATAACGAGGCGAGTATTGCCCGACTTGCAACTATTGCAGTCAACTATGCCAAAGCAGGCTGT
CAGGTAATCGCCCCTTCTGACATGATGGACGGAGGATCGGGGCAATAAAGAAAGGTCTCCATGCGGCAGGGCTTGGTAA
CAGAGTCAGTGTAATGAGCTACAGTGCCAAGTTTGCATCTAGTTTCTATGGACCTTTCCGTGATGCAGCTAAAAGTGCCC
CATCATTCGGCGATAGGAAGTGTTACCAACTGCCGCCTGGATCTATTGGTCTCGCAGAGAGGGCAGTGGACCGAGACGTG
GCTGAAGGGGCAGACATGTTGATGGTGAAACCTGGTCTTGCATACTTAGATGTCGTCAGAATGACAAAACAAAAATACCC
AACCCACCCGCTAGCAATCTATCACGTATCTGGGGAGTACGCCATGTTGCATCATGGCGCCACGCAGGGAGCGTTTGAGT
TGAAGGCAGTGGTACTGGAGTGTATGCAG
>Clanculus_margaritarius_1_foot_c45104_g1_i1 m.3780 c45104_g1_i1|g.3780 ORF
c45104_g1_i1|g.3780 c45104_g1_i1|m.3780 type:5prime_partial len:483 (-)
c45104_g1_i1:58-1506(-)
CGTGATAGTTGGCCATATTAGGGACTTTCTCCGTTACCATGGCTTTGAAAAAGTGAAAACATGCACAGAACCAAAACTTC
CGGGCTTTGTCCCGCTGTACGCTTCATGGGAAAGCTTCTTCACGCGGAACTTGTACAGACGGGTGCGGGATTGCTGGAAC
CGGCCTATTGGCAGTGTGGCGGGGGCACATATGGATCTCGTGGAGCGACGGACGCCAGACTATGGATGGAACTTTGAAAT
GACGGGTACAAAGAGACGAGTGATGAACTTCGGATCTTACAACTACCTAGGCTTCTCCGAGAATGAGGGGCCGTGTACTG
ACGTCGTGGAAGTTACCACGAGGGAGGAAGGGGTCGGGGTGTGCGCCGCCAGGCAGGAGCTTGGCTACTTTGATATTCAC
CGAGAACTGGATGAAATGACTGCAGAGTTTCTAGGCGTGGAGGCTGCAGTTTCCTTCCCAATGGGCTTTGCTACCAACTC
CATGAACATGCCGTGTCTTGTCAGTAAGGGTTGTTTGATTTTGAGTGATGAGTTGAACCACGCATCACTCGTATTAGGAG
CTCGCCTTTCTGGAGCCGGTATCAAGATCTATAAACATAACAATATGAAAGATTTAGAGAAGAAATTACGCGAGGCAATA
GTTCAAGGGCAGCCACGCACACATCGGCCCTGGAAGAAAATACTCATCGTGGTGGAGGGTGTGTACAGTATGGAAGGCTC
TATCGTCCGTCTCCCCGAGATAATCGCTTTAAAGAAGAAGTACAAAGCTTACTTGTACCTGGATGAAGCCCACAGTATAG
GAGCCATGGGGCCACATGGGCGTGGAGTTGTAGACTACTTCGGGCTGGATCCTCGAGATGTCGATATTCTCATGGGGACA
TTCACAAAGAGCTTCGGGGCAGCTGGGGGATACATCGCCGGGACGAAACAACTGATCAATCATCTACGAGTCCATTCACA
CAGTGCCATCTATGCATGTTCAATATCGCCACCAGTAGCACGACAGATCATACATTCCATGAAGACCATCATGGGTCGAG
ACGGAACCAATCTAGGCCGACAGAGGATCCAGCAGCTGGCGTGGAATATTCAGTATTTCCGGAAGGGCTTACAGAAGAGG
GGATTCATCGTGTATGGCAACAAAGACTCTCCAGTGGTTCCCTTGCTAATATATTTACCTGCTAAAACATGTGCGTTTAG
CAGGATGTGCAGTGAGCGAGGACTAGGCATTGTGGTGGTTGGTTTTCCAGCAACCCCTATAATTGAGTCTAGAGCTCGCT
TCTGTCTATCAGCCGCCCACACCAAGGATATGCTCGACAAGGCATTGGCTATTATAGACGAGGTAGGGGATTTGTTGTTC
ATCAAGTATTCACGGCTCACACCACCACCTTCCACCCACGAGGAGGAGTTAGAGAAACAAAAATCTTCAGTGTCAAATCA
TATACAGTGATGTGATCTATTTCAAGAAGAAGCTATAGTGGTGCTACAACTGGACCATTACAGGACC
>Clanculus_margaritarius_1_foot_c45732_g2_i1 m.5575 c45732_g2_i1|g.5575 ORF
c45732_g2_i1|g.5575 c45732_g2_i1|m.5575 type:5prime_partial len:301 (-)
c45732_g2_i1:286-1188(-)
GGTATCAGAACAAGTGGGGTACCGAAGCACATCTTCCGTCACAATGACCCCGATCACCTTGAGGAGCAGCTCAAGAAGGT
TGATGCCACCATCCCCAAAATAGTTGCCTTTGAGACAGTACATTCCATGGATGGATCTATCTGTCCCCTGAGGGAGATGT
GTGACGTGGCCCACAAGTACGGTGCCTTAACCTTCGTGGATGAAGTCCATGCTGTGGGGCTGTACGGCAAACATGGAGCT
GGCATTGCTGAAAGAGATGGATGTCCAGAAAAAGTTGACATTCTGTCAGGAACACTCGGAAAGGCATTTGGCAATATGGG
AGGCTACATAGTTGGGACGTCCAATACTATTGACATGATACGGAGCTATGCTGCTGGGTTCATCTTTACAACAGCTCTAC
CACCAACAACACTAAGTGGAGCCCTGGCATCCATTGATGTGTTGGCGAGTGATGAAGGCCGAGATCTCCGTGCCCAACAT
CAGAGTAACGTCCGCTACCTACGAGACAAGTTGACTCATGAAGGAATTCCGGCCATGCACAGCCCCAGTCACATCATACC
AATACACGTTGGCGATGCCCTCAAAGCTACCAAGGTGTCGAACGATCTTATCCAGGACCATGGCATTTACGTCCAGGCCA
TCAACTATCCGACAGTTGCTCGTGGACTGGAAAGACTTCGTGTGGCCCCAACTCCTCACCATACCCGGGAAATGATGGAC
GCCTTCGTTGACAGCGTAGTCGACACCTGGAAGGCAAATGACCTTGACCTTTACAAGCCGATCTGTCCAAAAACATGTGA
GAGCTGCAACAAACAACTCAAGCTCCAAGAGTTCTTCAAGCCTGATCCAGTTTGTTCCCGCTCTAACTGTACATACTCCT
CCCTTCAGGCGACTCTTGCATAAGGGCAACCTTGCATGTCACGTGTTCAGTTTGTGAACTTGACCTTAGTCCTGTAGCGA
TCAGGCTTTTATATTTGTGATGGAAAAATATTCTCCTACTGACTTACTTAACCAAAAACTCTTCGGCAGATAGTTTATTT
TCAGTCATTCCCAATGATATCTGTGGTCACTATGTTGATTAAACAGTTAGTCACCATGATACTAAAAATCACTAGATTCA
AAATCTTAATTAGAACACTTTGTTGTGAATTATTTCTGCATATTGAAAAGAAATTTAATTTATGATGC
>Clanculus_margaritarius_1_foot_c46228_g1_i1 m.3581 c46228_g1_i1|g.3581 ORF
c46228_g1_i1|g.3581 c46228_g1_i1|m.3581 type:complete len:368 (-)
c46228_g1_i1:225-1328(-)
CGGCTACGTTTAGGGGGCTTCCCACATCAATTAGGGCCCGCGTCCTTTCCCTTGGCCATAATAGTATAATAATTAGATTC
AGTATTAGATGCATTCGCTTCTGGTCTTTCAGACACTAGCTCCGCCCATAGGTTGTCACACTGAGGTGAAGAACAGTAAA
TCTGTGGAAGTCCCTCAATTTCACCCTGCGCGTCCAAGCCGTCAGCACTTGTGAAATTTCTCCATAAGCATGTCTACCTC
AACAAGGAAAACTCTTAGGATCGGGTCAAGAGAAAGCAAGCTCGCTTTGATCCAGTCCAACTATTTGATATCCTTGTTAA
AGGAGAAAAACCCGGATGTCAACTTTGAAACGGTTACCATGACGACACCCGGCGACAGAGATCTAAGCCAATCAATCGCG
AAGATCGGTAACGCAGCCCTTTGGTCACTTGAGCTGGAGAACTCTCTGAAGGCCGGTAAAGTGGACTTGATTGTCCACTC
TTTGAAGGACGTAGCTATACACCTACCTGATGGTTTCGTCCTAGGATGTATACTTAAGCGGGCAAACCCACACGATGCCA
TTGTCATGACAACACAAAATCAGGGGAAAACATTAGCAACGTTGCCAAAAGGAAGTGTTGTAGGCACCAGTTCGTTACGA

AGGGCGGCACAGATCAAAAGGAATTACCCTCACCTACAGATATTCGACATCCGAGGTAATATTGATGGTCGGATTAAGAA
TCTGGATGAGAGCGGGCAGTATGACGCACTGTGTCTAGCTGCCGCCGCACTCGAAAGAATGGGCCCCGAATATGAGAAGA
GAACATCACAGATCCTGTCTGCTAAAGACTGTATGTACTGTGTGAGTCAAGCGGCCCTGGCGGTGGAGTGTCGAGCAGAC
GACAAGCAGGTTCTGGACATCCTGGATCCTTTCCATGACAGGAACACCGTCATCAGAGTCGTCGCCGAGAGGGCGTACCT
TGGGAGGCTGATGGGCGGATGCAGCGAGCCTATAGGGACCGAGTCGGCCCTGGAGAACAACACTCTGACACTGAAGGGAG
GCGTGTTTAGTGCTGAGGGAGACGAGGCAGTCCTGGACGAAGAGACACAAGACCTGCCGGGAAATATCAAAGATATAATT
CAGGCAGCACCAAGTGGACCTCTAAAGCACTACGCGTCTATAGTGACGGGTAGTAGAATCAGTCCAGAAACTCTGCAGGC
TGCGGAGAAATTGGGATTGGATCTCGGAGACAGGATGGCCAAGAATGGGGCGGAGAGGATACTGGACGTTGTCCGAAAGG
AGATCGCAGCGTCCAAAAATGCCACTAAAGCAGCAAAGAATGGACATATCTGACTTAAACATGTCTAAGCGTACCGACCC
TGTTAGCCGGAGATTTTATATTAGTTCAGGATATTTTTTATTTTTGTTTTGTTGCCTGTAATGAAACATTATGGGTAATC
GTTTTCAAGAAATTTACCATATACAACATCAGAAGGATTTTACAGCTTGTTTAGGGTTTTGGTTTTAGATCCACAAAATC
GGTTTTATGAAGAGACCGTAAGTGATATTTGAAACAC
>Clanculus_margaritarius_1_foot_c98280_g1_i1 m.30855 c98280_g1_i1|g.30855 ORF
c98280_g1_i1|g.30855 c98280_g1_i1|m.30855 type:internal len:106 (-)
c98280_g1_i1:2-316(-)
CACACGACAAAAACTCGAAGGAAGAGTGCCGTTATTTGGATTCTCAGGAGCTCCGTGGACTTTGATGAAATATATGATAG
AAAACCTTGGTGCTGGACCAAGTCCAAACAAAGCACGCAGATTTCTTGTAGAGTACCCTGAAGCCGGCACGAAGCTGTTG
AAGATTCTGACGGGAGCAGTAGTACGCCATCTGGTGGAACAAGTTAGGGCTGGAGCACAGATACTTCAAGTCTTTGATTC
TAATGGTGGTGAATTGGGACCCAATCTGTTTACAAAATATGAACTGCCATGCCTACAGGAAATAGCGTACAAGGTCA
>Clanculus_margaritarius_2_mantle_c1654_g1_i1 m.8370 c1654_g1_i1|g.8370 ORF
c1654_g1_i1|g.8370 c1654_g1_i1|m.8370 type:5prime_partial len:166 (-)
c1654_g1_i1:95-592(-)
ACAGCACAGAGGTCGCGGCGACAGTCCAACGTGTAATGGAGGATCTTGGTCACAGTCATGCCTACCGACTGGTCTGGCAG
TCCAAGGTTGGTCCACTGCCCTGGTTGAGCCCACAGACAGATGATGCTATCAAGGGTCTGGTTGCGAGAGGTCGCAAGAA
TCTACTTCTCGTGCCGATAGCCTTCACCAGCGACCATATTGAAACCTTGTTTGAGTTGGACTACGAGTACGCACAGAATC
TTGGGGCAGAGGTCGGTGTCAAGAACATCAGACGGGCAGCATCACTGAATGACAACCCTGTCTTCATTGAGGCCCTGGCC
GACCTGGTGAAGACACATCTACACACCCAGCAAGTGTGCACACCTCAGCTCCTTCTCCGCTGTCCGATGTGCACCAACCT
GGTGTGCGGTCTCGCCAAAGAGTTCTTTCGTGATCAACAGCCCATCCTTGACACTCTACGAGCTGAGGACGAGGAACTGA
AACTAGGAGCCAAGTCTTGATGTACTTCCAGTCTTCTGCAATCTGATTAAAATCATTGTTTGATATGCAGGGTCAGGAAA
TAAGTTCTGATTCAAAGACTAACATTAACCCCGT
>Clanculus_margaritarius_2_mantle_c20351_g1_i1 m.1848 c20351_g1_i1|g.1848 ORF
c20351_g1_i1|g.1848 c20351_g1_i1|m.1848 type:complete len:367 (+)
c20351_g1_i1:171-1271(+)
AATAGTATAATAATTAGATTCAGCATTAGATGCATTCGCTTCTGGTCTTTCAGACACTAGCTCCGCCCATAGGTTGTCAC
ACTGAGGTGAAGAACAGTAATTCTGTGGAAGTCCCTCAATTTCACCCTGCGCTTCCAAGCCGTCAGCACTTGTGAAATTT
CTCCATAAGCATGTCTACCTCAACAAGGAAAACTCTTAGGATCGGATCAAGAGAAAGCAAGCTCGCTTTGATCCAGTCCA
ACTATTTGATATCCCTGTTAAAGGAGAAAAACCCGGATGTCAACTTTGAAACGGTTACCATGACGACACCCGGAGACCGA
GATCTAAGCCAATCAATCGCGAAGATCGGTAACGCAGCCCTTTGGTCACTTGAGCTGGAGAACTCTCTGAAGGCCGGTAA
AGTGGACTTGATTGTCCACTCTTTGAAGGACGTTGCTATAAACCTACCTGATGGCCTCGTCCTGGGATGTATACTTAAGC
GAGCAAACCCACACGATGCCATTGTCATGACAACACAAAATCAGGGGAAAACATTAGCAACGTTGCCAAAAGGAAGTGTT
GTAGGCACCAGTTCTTTACGAAGAGCGGCACAGATCAAAAGGAATTACCCTCACCTACAGATATTCGACATCCGAGGTAA
TATTGATGGTCGGATTAAGAATCTGGATGAGAGCGGGCAGTATGACGCACTGTGTCTAGCTGCCGCCGCACTCGAAAGAA
TGGGCCCCGAATATGAGAAGAGAACATCACAGATCCTGTCTGCTAAAGACTGTATGTACTGTGTGAGTCAAGCCGCACTG
GCGGTGGAGTGTCGAGCAGACGACAAGCAGGTTCTGGACATCCTGGATCCATTCCATGACAGGAACACCGTCATCAGAGT
CGTCGCCGAGAGGGCGTACCTAGGGAGGCTGATGGGCGGATGCAGCGAGCCTATAGGGACCGTGTCGGCCCTGGAGAACA
ACACTCTGACACTGAAGGGGGGAGTGTTTAGCGCTGAGGGAGACGAGGCAGTGCTAGATGAAGAGACACGGGACCTGCCG
GGAAATATCAAAGAGATCATTCAGGCACCAGGTGGACTTCTAAAGCCCTACGCATCTATAGTGACGGGAAGTAGGATCAG
TCCAGAAACGCTGCAGGCTGCGGAGAAATTGGGACTGGATCTCGGGGACAGGATGGCCAAGAATGGGGCGGAGAGGATAC
TGGACGTTGTCCGAAAAGAGATCGCAGCGTCCAAAGTTGCCACTAAAGCAGCAAAGAATGGACATATCTGACTTAAACAT
GTCTAAGCGTACCGACCCTGTTAGCCGGAGATTTTATATTAGTTCAGGATATTTTTTATTTTTGTTTTGTTGCCTGTAAT
GAAACATTATGGGTATGCCGGTAATCGTTTTCAAGAAATTTACCATATACAACATCAGAAGGATTTTACAGCTTGTTTAG
GGTTTTGGTTTTAGATCCACAAAATCGGATTTTTGAAACACTGTCCTGTAGAGTTGTTTCTTTTCATAGGCTTACGACTG
TGGTAGCACAGAGATCTGTTTGGAAACAGCGCCCATTCGGCGCCGTTTCCA
>Clanculus_margaritarius_2_mantle_c20351_g1_i2 m.2003 c20351_g1_i2|g.2003 ORF
c20351_g1_i2|g.2003 c20351_g1_i2|m.2003 type:complete len:367 (+)
c20351_g1_i2:171-1271(+)
AATAGTATAATAATTAGATTCAGCATTAGATGCATTCGCTTCTGGTCTTTCAGACACTAGCTCCGCCCATAGGTTGTCAC
ACTGAGGTGAAGAACAGTAATTCTGTGGAAGTCCCTCAATTTCACCCTGCGCTTCCAAGCCGTCAGCACTTGTGAAATTT
CTCCATAAGCATGTCTACCTCAACAAGGAAAACTCTTAGGATCGGATCAAGAGAAAGCAAGCTCGCTTTGATCCAGTCCA
ACTATTTGATATCCCTGTTAAAGGAGAAAAACCCGGATGTCAACTTTGAAACGGTTACCATGACGACACCCGGGGACAGA

GATCTAAGCCAATCAATCGCGAAGATCGGTAACGCAGCCCTTTGGTCACTTGAGCTGGAGAACTCTCTGAAGGCCGGTAA
AGTGGACTTGATTGTCCACTCTTTGAAGGACGTCGCTATAAACCTACCTGATGGTCTCGTCCTAGGATGTATACTTAAGC
GAGCAAACCCACACGATGCCATTGTCATGACAACACAAAATCAGGGGAAAACATTAGCAACGTTGCCAAAAGGAAGTGTT
GTAGGCACCAGTTCTTTACGAAGAGCGGCACAGATCAAAAGGAATTACCCTCACCTACAGATATTCGACATCCGAGGTAA
TATTGATGGTCGGATTAAGAATCTGGATGAGAGCGGACAGTATGACGCACTGTGTCTGGCTGCAGCAGCACTCGAAAGAA
TGGGCCCCGAATATGAGAAGAGAACATCACAGATCCTGTCTGCTAAAGACTGTATGTACTGTGTGAGTCAAGCCGCACTG
GCGGTGGAGTGTCGAGCAGACGACAAGCAGGTTCTGGACATCCTGGATCCATTCCATGACAGGAACACCGTCATCAGAGT
CGTCGCCGAGAGGGCGTACCTAGGGAGGCTGATGGGCGGATGCAGCGAGCCTATAGGGACCGTGTCGGCCCTGGAGAACA
ACACTCTGACACTGAAGGGAGGCGTGTTTAGTGCTGAGGGAGACGAGGCAGTCCTGGACGAAGAGGCGCAAGACCTGCCG
GGAAATATCAAAGATATCATTCAGGCACCAAGTGGACCTCTAAAGCACTACGCGTCTATAGTGACGGGTAGTAGAATCAG
TCCAGAAACTCTGCAGGCTGCAGAGAAATTGGGATTGGATCTCGGAGACAGGATGGCCAAGAAGGGGGCAGAGAGGATAC
TGGACGTTGTCCGAAAGGAGATCGCAGCGTCCAAAGATGCCACTAAAGCAGCAAAGAATGGACATATCTGACTTAAACAT
GTCTAAGCGTACCGACCCTGTTAGCCGGAGATTTTATATTAGTTCAGGATATTTTTTATTTTTGTTTTGTTGCCTGTAAT
GAAACATTATGGGTATGCCGGTAATCGTTTTCAAGAAATTTACCATATACAACATCAGAAGGATTTTACAGCTTGTTTAG
GGTTATGGTTTTAGATCCACAAAATCGGTTTTATGAAGAGAGCGTAAGTGATTTTTGAAACACTGT
>Clanculus_margaritarius_2_mantle_c20500_g1_i1 m.2200 c20500_g1_i1|g.2200 ORF
c20500_g1_i1|g.2200 c20500_g1_i1|m.2200 type:complete len:330 (-)
c20500_g1_i1:217-1206(-)
ACTCCGAGCGGACGCTCTACCTCTGCACCACGGAGGCGGTCAGTCATGTTTGATGTTACAGCCCAGAAAAAGTCCTGTGT
CATCCAATAGGCCCCGCCCACTCCCGCGTTGATTGGCTATAAATGTTCCAACAGAAGCGTGGAGTATATCTGCACGATAT
CGGGACCCAGCGTCATATTGCCGTGTGTTTACAAACCCTTGCTTAGGTCAGTCCTGATTGAAGTCATGGCGGATCACGCA
GTTCTTCACAGTGGCTATCACCATCCGGTTCTACGGGCGTGGAATTCAGGCAACACTTCAGTAACGCCAAACAACCTCAT
ATACCCGCTATTTATCGTTGACGAGGAGGATGCAGTCCAAGAAATACCCAGCATGCCTGGCCAGTCCAGATATGGCGTCA
AGCGTCTACAAGATGCCATTGAACCACTTGTTAAGAAAGGGTTGAAAACTGTACTCCTTTTTGGTGTTCCTGGAAAAATT
TATAAGGATAATGAAGGCAGTGGTGCTGACATGCCAACAACTCCAGTTATACAAGCCATCACATTAATACGCAAGTGGTT
CCCGGAACTGTTAGTAGCGTGTGATGTGTGTCTGTGTCCCTACACCTGCCACGGACACTGTGGTATTCTAAGAGAAGATG
GCTCATTAGATAATGAGGCGAGTATTGCCCGACTTGCCACTATCGCAGTCAACTATGCCAAAGCAGGCTGTCAGGTAATC
GCCCCTTCTGACATGATGGACGGAAGGATCGGGGCAATAAAGAAAGGTCTCCATGCGGCAGGGCTTGGTAACAGAGTCAG
TGTAATGAGCTACAGTGCCAAGTTTGCATCTAGTTTCTATGGACCTTTCCGTGATGCAGCTAAAAGTGCCCCATCGTTCG
GAGACAGGAAGTGTTACCAGCTGCCACCTGGATCTATTGGTCTCGCAGAGAGGGCAGTGGACCGAGATGTGGTTGAGGGA
GCAGACATGTTGATGGTGAAACCTGGCCTTGCATACTTAGATGTCGTCAGAATGACAAAACAAAAATACCCAACCCACCC
GCTAGCAATCTATCACGTATCTGGGGAGTATGCCATGTTGCATCATGGCGCCACGCAGGGAGCGTTTGAGTTGAAGGCAG
TGGTACTGGAGTGTCTGCAGAGTATGAGACGGGCAGGTGCTGATATAATTATCACCTACTTCGTTCCTGATCTCCTGGAC
TGGATTAAGTCATAGACTATCAGTGTCAGTTGTCATTGCACTTGAAAGTTGTCTACATTTTGGTCATTGGAAATATTACA
CATGGGCAAAACAAACTACCCTTTTGTGTGCAGAATTAAACTTTTGTAAGGGCATTGTGCCATCCTGTTATGAATTACCG
TATTCGACCCTATAACCGTCCATGCCCCTATAAGCGCCCACAATGCGTCAGGGCTCTGTTGTTCAAAGCTC
>Clanculus_margaritarius_2_mantle_c20663_g1_i2 m.286 c20663_g1_i2|g.286 ORF
c20663_g1_i2|g.286 c20663_g1_i2|m.286 type:complete len:606 (-)
c20663_g1_i2:1461-3278(-)
TTGCCACCATTACCTGCCTGTCAATGTGTCACCTGTGAAGAGGTCTATCAAGCAAGCACCTGTTTCCAGCTGTGAGAACA
ATTAATATACTCGGCAACCATGAAGCGACTTGCATGTCCATTTCTCACCAAGATACCAGTCAACAACCTACGGCAGTTTC
CTCAGCAGCTGCTGTCCTTTGCCAACCGATGTCCGGTGATGAACCACGCCGTTCACTACTCGTCCAGTGTTGCTGCCAGC
CAGGACAGTCCCAACATGGGAGAGGGGTTGAAGTGCCCGTACCTGGCCAATGAGATGACAGTTCAAGAAGCCAGTCCAGC
CATCCAACAAGATGTCATTGCTGTTAATAGACAAGAGGACAGTATGTTTCTTGCCCAGGAGAGCTCTGGTCTCAGCAACA
CTGTGGCAGAACATGCAGCGTCCACCGCCGCCTCGGACAGTGTGACAGTCATGACAGGAGATACTGGGGTGGAGAAGAGC
TCCAACTCAGGGGGGATGCAGGGCTGGAACTTCAGTGAGACCCTCAAGATGCTCAAGAAATCTGGATCGGGCCCCAGTGG
AGGAAAAACCTCAGGATCTGTTCAACTATGAAGCCTTCTTTGCAAGTGAAGTGGACAAGAAGAAAAGAGACCATTCCTATC
GTATTTTCAAGAAAGTGATGCGCAATGCAAGTACCTTTCCTTTTGCTCAGGAACATTCAGAAGGCCAGATGGACATCTCG
GTGTGGTGTAGTAACGACTATCTGGGGATGAGCTGGCATCCAGAAGTCAGGAACGCTGTAAGGACCGCACTGGACAAACA
TGGAGCTGGTGCAGGTGGAACAAGGAACATCTCGGGAAACTCGCCACTCCATGAACGTCTTGAACAACGTCTTGCTCGTC
TGCACGACAAGGAGGCTGGCCTCATCTTCACCTCATGCTATGTTGCCAATGACTCCACTCTGTTTACCTTACTCAGAGCA
CTTCCAGGTTGCCATATTTTCTCGGACGCGGGTAACCATGCCTCCATGATTGCTGGTATCAGAACAAGTGGGGTACCGAA
GCACATCTTCCGTCACAATGACCCCGATCACCTTGAGGAGCAGCTCAAGAAGGTTGATGCCACCATCCCCAAAATAGTTG
CCTTTGAGACAGTACATTCCATGGATGGATCTATCTGTCCCCTGAGAGAGATGTGTGACGTGGCCCACAAGTACGGTGCC
TTAACCTTCGTGGATGAAGTCCATGCTGTGGGACTGTACGGCAAACATGGAGCTGGCATTGCTGAGAGAGATGGATGTCC
AGAGAAAGTTGACATTCTGTCAGGAACACTCGGAAAGGCATTTGGCAATATGGGAGGCTACATAGTTGGGACGTCCAATA
CTATTGACATGATACGGAGCTATGCCGCTGGGTTCATCTTTACAACAGCTCTACCACCAACAACACTAAGTGGAGCCCTG
GCATCCATTGATGTGTTGGCGAGTGATGAAGGCCGAGATCTCCGTGCCCAACATCAGAGTAACGTCCGCTACCTACGAGA
CAAGTTGACTCATGAAGGAATTCCGGCCATGCACAGCCCCAGTCACATCATACCAATACACGTTGGCGATGCCCTCAAAG
CTACCAAGGTGTCGAACGATCTTATCCAGGACCATGGCATTTACGTCCAGGCCATCAACTATCCGACAGTCGCTCGTGGA

CTGGAAAGACTTCGTGTGGCCCCAACTCCTCACCATACCCGGGAAATGATGGACGCCTTCGTTGACAGCGTAGTCGACAC
CTGGAAGGCAAATGACCTTGACCTTTACAAGCCGATCTGTCCAAAAACATGTGAGAGCTGCAACAAACAACTCAAGCTCC
AAGAGTTCTTCAAGCCTGATCCAGTTTGTTCCCGCTCTAACTGTACATACTCTTCCCTTCAGGCGACTCTTGCATAAGGG
CAACCTTGCATGTCACATGTTCAGTTTGTGACCTTGACCTTAGTCCTGTAGCGACCAGGCTTTTATATTTGTGATTGAAA
TATATTCTCCTACTGACTTACTTAACCATAAACCCTTTGGCAGACAGTTTATTTTCAGTCATTCCCAATAATATCTGTGG
TCATTATGTCGATTAAACAGTTAGTCACCATGATACTGAAAATCACTAGATTCAAAATCTTAATTAGAACACTTTGTTGT
GAATTATTTCTGCATATTGAAAAGAAATTTAATTTATGATGCGAAAATTTAAGAATATATTAGAAGTGACTGGTTATTAA
CCAGGATGAATTTATTTATCTACATACAAGTGTTTTGTGCAAAACTTGGAACCAGGTTTGAATTGGTCTTATTCAATATT
TGGTGTATATTATCGTCTCCAATAAATGGGTATATTAGTGTGTTCATTTCATACAAATGTTTTATAATAAACATGTGCAA
TTATTAGTCATACTTTGTCTCAATAAAATTTCAATTTTTAATAAAATTTTTATTATTATCTGTCAGGTTTAAAACCATTA
AGGTTTTGTGCTAAAAAAGATGACCTTAAGAGGTATAACTATAATTGTATCATTAAATAATTGCTTGTAAAGTGGTTCAC
AGATTTTAAAACAATAAATAATTAATGTAAAAGTTATTTTAGCTGATCTTATTTGTTACAGAAAATGTAAGTTAATTAAG
TTAATTAATAACAGTCATGTTTGGAATCTCTTATCTGATGAACAAACTGACATTTGGGTCAATATTGTGATTGTTTCTTG
TGGGTTTGATACATAGTTGATTTTTGTTTGTTTGTAATTTTTCTAGATTCCAGTAATGATGTACATTATGTACATCCATG
ATTCCTGTGATCGTTGGGCACATTTTGTGTAATACAATTGTTTAATTATATATTGTCTTTATTTTGTAAAAAAAAAATTCA
GTTTATGAGCAAACATTTTAAAACTACAAAGGTAATCAATTCCATTACAAAAATGACCAAATAATCTGAAGAAATGCATT
CAGTAGAAATAAACTATAAACATGATAAATGCTTTATAAAAAAGTTTCTTCAAATGTTTGAATAATGAATTCAAAACATA
TTATTGATTAAGCGTATTTAGATGCTGTAATTGATGTATTTAATCGGTCCTTGACTACAGTTCGGACAGATCAAACATGT
ACATAAAACTGTGTCCTTTTACTGATAAACAAAGTAACAGGCGAACCTTTCAAATTCAGGATCTTTGGTCTGACTTCACA
AAACACAACAGTAAAGAATTAAACACCAAATATGGGTGTGGCTATACTTGTATGGGCGTGGTTATAATTAGTGGTTCATT
TTGTTTTTGTTTTGAATTAGACAATTTTTTTTAGTTCTAGTTCAGGGCCCAATTTCACAAAGCAACTTTAGCACTAAGGT
AACCTTAGTGATTTTAA
>Clanculus_margaritarius_2_mantle_c68931_g1_i1 m.6651 c68931_g1_i1|g.6651 ORF
c68931_g1_i1|g.6651 c68931_g1_i1|m.6651 type:internal len:232 (+)
c68931_g1_i1:2-694(+)
GCCAAAAGGGAGTGTGATTGGTACCAGTTCCTTAAGGCGATGTGCACAGATAAAGAGAAACTATCCACATTTTGAAATCA
AAGATATTAGGGGTAATCTGAATACACGGTTCAAGAAACTTGATGAGGATGATGTTTATGATGCTATAATATTAGCTGTG
GCTGATCTAGATCGAATGGAATGGTCTGATCGAATATCTCAGAAGTTGCCTGCTGATGTGTGTATGTATGCTGTGAGCCA
GGGGGCTATAGCAGTAGAATGCCGATTGAGGGACAAGAGTATCCTGGATATTCTGTATCCACTGCATCACCGGGAAACAG
CCCTACGATGTGTGGCAGAGAGGGCGTATCTCAGGAGACTGGAGGGTGGCTGTACTGTACCAGTGTCAGTTTTCACAGAG
ATCAAGGATGATAAGATGTCAATCAGTGGAGGTGTGTACAGCACAGATGGTGTACAGGCAGTGGAAGACTCACTGGAGTC
AGCATCTCTTTCTCAGAAGGAACAGGAGAGTGTAACAAATGGCTCCAGCACCTATTGCTCCATTGTCTGTGGCAAGGGAA
ACAACTCCAAGAGTTTCCTCGAGGCAGAGAAGCTGGGAATTGACCTCGCTGACAAGATGGTGAGACAAGGTGCAGGGGAG
ATATTAAGGATAGCTAAAAGAAAAGCAAGAGATGCGATTGAGGATGAGCGGGCA
>Calliostoma_zizyphinum_mantle_c10706_g1_i1 m.15232 c10706_g1_i1|g.15232 ORF
c10706_g1_i1|g.15232 c10706_g1_i1|m.15232 type:3prime_partial len:180 (-)
c10706_g1_i1:3-539(-)
CTGGCAGTTCATAAGGAGTATTTTCTATAACTGAGGCTTTATTACCAATGTTTTTAGGTCTGTTGCAGAGAAGTGGGTG
TGTCCGAAATGTAGGCGTCGCCCCTCAAGTGTGCATCTTTGATCCTCAAAGTTACGACACAAGACATGCAAGTTCTCACA
GTGGTCCGAAGACAGGTGTGATGATGTTGAATCTGGGAGGACCAGAAAAGACGGAAGATGTCCATGACTTCCTTCTGCGA
CTCTTCTTGGACAAGGATCTCATTCCACTACCTGCACAGAGCAAGCTTGCTCCAATCATTGCCAAGCGAAGAACTCCGTC
CATTCAACGACAGTATCGGAAGATTGGCGGTGGGTCGCCTATCAAACGCTGGACAGAATTACAAGGTCAAGGGATGGTGG
ATATACTGGACAAGATCAGTCCGGAAACAGCACCTCACAAGTTCTATGTGGGATTTCGTTACGCCCATCCGCTGACAGAA
GACATGATAGAACAGATGGAAGCGGATGGTATAGAAAGAGCCATTGCCTTCACCCAGTACCCACAATACAGCTGCTCTAC
AACAGGAAGTAGTTTGAACGCCATCTTTCGTCACTACCTCAAGCGTACCGGCCCCAGTAACCTTGTATGGAGTGTTATTG
ATCGATGGCCAACACATCCTGGCCTTGTTAAGGCATTTACACAAAACATACGTGAAGAAATTGCCAAGTTCCCGAACGA
>Calliostoma_zizyphinum_mantle_c10706_g1_i2 m.14528 c10706_g1_i2|g.14528 ORF
c10706_g1_i2|g.14528 c10706_g1_i2|m.14528 type:3prime_partial len:231 (-)
c10706_g1_i2:3-692(-)
CTAAATCGTTTTCGTGCAAGAAGGACGCACTTGATAACACGCAAGGAGAACCATGGCAAAAATCTTGGGTCGCCGGCTTA
CAGGTCTGTTGCAGAGAAGTGGGTGTGTCCGAAATGTAGGCGTCGCCCCTCAAGTGTGCATCTTTGATCCTCAAAGTTAC
GACACAAGACATGCAAGTTCTCACAGTGGTCCGAAGACAGGTGTGATGATGTTGAATCTGGGAGGACCAGAAAAGACGGA
AGATGTCCATGACTTCCTTCTGCGACTCTTCTTGGACAAGGATCTCATTCCACTACCTGCACAGAGCAAGCTTGCTCCAA
TCATTGCCAAGCGAAGAACTCCGTCCATTCAACGACAGTATCGGAAGATTGGCGGTGGGTCGCCTATCAAACGCTGGACA
GAATTACAAGGTCAAGGGATGGTGGATATACTGGACAAGATCAGTCCGGAAACAGCACCTCACAAGTTCTATGTGGGATT
TCGTTACGCCCATCCGCTGACAGAAGACATGATAGAACAGATGGAAGCGGATGGTATAGAAAGAGCCATTGCCTTCACCC
AGTACCCACAATACAGCTGCTCTACAACAGGAAGTAGTTTGAACGCCATCTTTCGTCACTACCTCAAGCGTACCGGCCCC
AGTAACCTTGTATGGAGTGTTATTGATCGATGGCCAACACATCCTGGCCTTGTTAAGGCATTTACACAAAACATACGTGA
AGAAATTGCCAAGTTCCCGAACGA

>Calliostoma_zizyphinum_mantle_c22534_g1_i1 m.16262 c22534_g1_i1|g.16262 ORF c22534_g1_i1|g.16262 c22534_g1_i1|m.16262 type:3prime_partial len:194 (+) c22534_g1_i1:109-687(+)
TGCCGCGAATGTAAACTAGGATACGTGCACAGTGCTGCAGAGCGTGAGAATGCCTGGTCAGTTCAACATGTCCCTGTGTA
AAGTGTGTTGACGGAGAGTTTATTGTTGATGACGATGTCGACTGCGGTGGTGTTAGGTGGCGGAGTGAGCGGACTGGCAG
CGGTCTATTACCTGCAGAAGTTCGCAGGTAACAAATTTGCGAAGATCATACTCTTGGAGGCATCGGACCGACTGGGCGGC
TGGGTTCAGTCTACCAGGTGTGACAATGGTGCTGTGTTTGAACGAGGACCACGTAGTCTCCGACCCGTTGGCGACTCGGG
AATAAACACACTTTTATTGGTGGAGGAACTTGGACTGAGTAGAAACGTTCTGCCATGTCTTAAGTCGAGTGCTCCAGCCA
AAAACCGATTCTTATACTTGAATAAGCAGCTGCATACACTACCGAACAGTTTTAAGTCCGTGGTAACAACTCAGACTCCG
TTTTCAAAACCGGTTTTATTGACGGCCCTTAAAGAGATGATTACAAAAAGGGCAGACAACTCTGATGAGACGGTTCATAG
CTTCATATCACGTAGATTTGGTTGTGAGCTTGCTGACTATGCAGTAGATCCGTTGTGTCGAGGAATATTTGCAGGCGATT
GCAGAGTTCTGAGTATGGACGCCTGTTTTCCGATGCTGACTGCATGG
>Calliostoma_zizyphinum_mantle_c25229_g1_i1 m.33589 c25229_g1_i1|g.33589 ORF c25229_g1_i1|g.33589 c25229_g1_i1|m.33589 type:internal len:124 (+) c25229_g1_i1:2-370(+)
GGGCCTCGTCACAAGGATGGCAGGTGTCATATCACAGTGTGTTCACACCCTTCACAATCTGACAGTTGTCACGGTAACCA
GACTTTCACCACATTTAAAAAAACACAGGTACAGTTTTCTGGGCCTGATTGTTGGTGGGACGGCATATTATCAAACACAT
GGAAAATCAAAGGCTTATGCACAGGCCATGGACATCCAGTCCAAGCGCTCACAGTGGATGGCTGAATCTATCACTGACAA
AGACCAGCTTCAAAAGAATTCCAGTGACATGAGGCATCGAATGGAAATGATGATAATGAGAATACAGGGCGAGGTGTGTC
GAGCTCTGGAGGAGGCAGATGGAGAGAAGAAATTTATGGTGGACCGGAGGG
>Calliostoma_zizyphinum_mantle_c25737_g1_i1 m.8207 c25737_g1_i1|g.8207 ORF c25737_g1_i1|g.8207 c25737_g1_i1|m.8207 type:5prime_partial len:286 (+) c25737_g1_i1:2-859(+)
GACTGTGATGGAGAGAGAGCATGGCTCTGTTATAAGAGGAATGCTTATCCCAAAAAAGAAATCAATTCCGTCTTCTGCAC
TGATCTCGAAGTCCAGATCAGAAAGTTGGGCATCATGGTCCCTGTCAACTGGGATGCAACAACTCACGGACACAATGGCT
GATGTGATTGGTCAGGATAAGAGGTCAGAGGTCAGGAGATTATCGCCCTGTACTGGAGTCAAAATACAGCATGATGGGAA
AATCAAGATTTCAACTGAGGGTGAAGATATTCTGGCTGACCATGTGTTCTCTTCATTGTATGCTAAATGTCTTGGCAGCC
TTCTTCCGCACAGTCCCCTCCATAAGGGTCTCATGTCGATATCAGCTGTGTCCGTCGCCGTGGTCAATCTGGAGTACCAG
GGGCACCTGCTTCCTGTGGAGGGGTTCGGTCACCTGCTGCCATCAATGGAGAGCGGCCCTGTATTGGGAGTAGTTTACGA
CTCGTGTGCATTCCCAGAACACAACCGTAAAGACATGGCGGCCAGTACGCGACTAACAGTGATGCTGGGAGGGGCATGGT
TCGATGAACTGGTAGCACTGACGAATCTTGATAAATCTCGGGTGGAGCAGCTTGCCATAGACACAGTGAGACAACACCTC
AACATCACCAGCAGTCCATGCTTCACCCAGGTGGCCTTACAGAAGGACTGTATACCTCAGTACATTGTTGGACACAACCG
CAATCTTGCAGTCCTGGAGAAATACCTCGCAGATGAAAAGATCCCTTTGACATTGATTGGCTCTTCATATCGCGGGCCTA
GCATCAACGACTGCATCAACAATACACGCCTAGCTGTGGAGAAAACCTGTGTCACATGACAGAGTGGATAGATTTACACA
TGACAGAGTGGATAGATTTAAACATAATGTGATAAAATTATCTGTGTCACATAATAAAGTAGATAAGTTTAAACATTATA
CTACTAATATAAATCCACATTCTAGGCATATTAATTCATTTAAGTAAATAAGCGTCCTGAATGTAGAATAGATAAAATAC
TTATATCCTAGAATACCTTTAACCATAACCGTGCGGC
>Calliostoma_zizyphinum_mantle_c26554_g1_i1 m.11751 c26554_g1_i1|g.11751 ORF c26554_g1_i1|g.11751 c26554_g1_i1|m.11751 type:internal len:288 (-) c26554_g1_i1:2-862(-)
ACAAGAATGGCCGCGACCCAAACACATGCACAGATGCCCGCACAGGAGTTCGCTCCCCTTCAAAATGACCTGGTGCTACG
TGCCGCTAGAGGTGAGAAGACGGAGAAAGTGCCGGTATGGGTGATGAGACAAGCTGGGCGCTACCTGCCAGAGTACCTCA
AAGCTAAAGGAGATAAAGCTTTTTTCGCTACCTGTAGAGATAAGGAGCTTGTTAGTGAACTGACAATACAGCCCATTGAC
AGATTTTCGCTGGATGCAGCTATAATATTTTCAGACATTTTGGTTATCCCCCTTGCACTTGGGTTGAAGGCACAGAATGA
TCCTGGACAGGGCCCGGTTTTTGCCGACCCTATCCAAAAGCCTGAAGATTTGGACCGACTCAATCCAGACTTCAACATCA
TCGAGGAACTGGGCTATGTGTATGAAGCCATCACACTCACCAGACAAAAGCTCAAGGGAAGGGTGCCACTATTTGGCTTC
TCCGGTGCTCCATGGACATTGATGAAGTACATGATAGAGAATCTCGGTGCTGGACCCAACCCCAACAAGACGCGGCGCTT
CCTTATAGAGCATCCCGATGCTGGCGAGAAACTGCTGCAGATCCTGACCGACGCAGTAGTGCGCCACCTGGTGGAGCAGG
TTCGAGCTGGAGCGCAGATTTTACAAGTGTTTGACTCCAGCTGTGGTGAGTTGGGTCCCACTCTGTTCACAAAGTTTGAG
TTGCCATGTCTGAAAGAGATCGCATTCAGAGTCAAGAAGACTGTCAAAGAAGAGGGGCTGGAGCTGATTCCAATGGTTGT
CTTTGCCAAGGATGCCTACTTTGCAACGAAACAGCTTGCTGGTACAGACTATGATGTGATGC
>Calliostoma_zizyphinum_mantle_c29010_g1_i1 m.8838 c29010_g1_i1|g.8838 ORF c29010_g1_i1|g.8838 c29010_g1_i1|m.8838 type:5prime_partial len:273 (+) c29010_g1_i1:2-820(+)
CCGCCGATGGGAAAGGGAAGAGGGCGGGGGAGGTATCTCCTGTGTTATGCAAGATGCCACTGTGTTTGAGAAAGCCGGGG
TTAACATATCCATTGTCCATGGCAACCTCCCCCCTAGTGCTGTGCAACAGATGAAATCAAGAGGAAAATTGTTGGAGGGC
TCCTCTCTTCCATTCTTTGCTGCTGGCATCAGTGCAGTGATACACCCAAAAAATCCGCATATACCAACAGTTCACTTTAA
CTACCGGTACTTTGAAGTGGAAACGAAAACAGGCAAGCAGTGGTGGTTTGGAGGTGGGACTGATCTCACACCCAACTATC
TAGTTGAAGAGGATGTTGTTAACTTCCATAAGACACTGAAGGCTGCCTGTGACAAGCACAATAAGTCTTACTATGGCCGA
TTCAAGAAGTGGTGCGACAACTACTTCTTCATCAAACACAGAGGGGAGAGTCGGGGTGTTGGAGGAATATTCTTCGACGA

CATTGATGAGCCGAATCCAGATGCAGCTTTCAAGTTTGTCACATCATGTGCAGAATCTGTGGTGCCCTCCTACTTGCCGA
TTGTCTTGAAGCACAAGTTCGACGGTTACAGCTATGATGAAAGGAAGTGGCAGCTACTGCGACGAGGGCGGTATGTGGAG
TTTAACCTCATATACGACCGCGGCACCAAGTTCGGTCTGTACACCCCCGGGGCGAGATATGAGAGTATCCTCATGTCACT
TCCTCTGAATGCTAGATGGGAGTACTGCCACTCGCCAACCCCAGGATCAAAAGAGGCCAAGCTAACAGAAGTGCTCCGCA
ACCCAAGGGACTGGGTATAAGGGGAGGAAGACCGGTAGTAATCATCAAAATCTACAATATGCAATTAGTTGAAGACGTGC
CAAGACATATACACTATACTTGCAAAGTGCTAATATAATTTCATTCACATACAGTAGTACAGTTCGCATTCCATGATTTT
GTTTTATGTAAAACAAATGAAAGAAGTCAACTTTGTAAGAGTTGCATTTTCACTTTAAAAAAAAATATAGTGA
>Calliostoma_zizyphinum_mantle_c2993_g1_i1 m.12913 c2993_g1_i1|g.12913 ORF
c2993_g1_i1|g.12913 c2993_g1_i1|m.12913 type:internal len:270 (+) c2993_g1_i1:2-
808(+)
GACACAAGACAAGGACGGCCGATTTTATCATCATTGTATCAAGATGTCGACGAAGAAGATGATTAAAGTGGGATCCCGCA
AGAGCAACCTTGCTCTGATTCAAACCAACACTGTGATAGACAAACTGAAGAAGGTTCATCCACATTTAGAGTTTGATGTT
GTTACCATGACAACTGTTGGAGACAGAATTCTTGACTCAGCTCTCTCTAAGATTGGCGAAAAAGCTTTATTTACAAGAGA
ACTGGAAGATTCTTTGCTGGACGGTACAGTTTCCTTTGTTGTGCATTCTCTGAAAGACCTGCCCACATCACTTCCTGATG
ATCTCATTATCGGCTGTGTTAATGAACGAGATGATCCTCACGATGCTGTCGTCATGCACAAGAAACACAACTATAAAACT
CTAGATGATCTGCCAGAGGGAAGTGTTGTGGGTACCAGCTCCCTGAGACGCTGTGCACAGATTCGAAGACGTTACCCACA
CCTGGAGATCAAAGATATCAGAGGAAACCTCAACACAAGGTTTCGAAAACTGGACGAAGATGACGTGTACGATGCCATTG
TACTGGCTGTAGCCGGTTTGGACCGGATGGACTGGTCAGACCGAATATCACAGAAGCTGCCGGTAGATGTGTGTATGTAT
GCAGTGAGCCAAGGAGCAGTGGCGGTGGAATGTCGAGCCACAGACACGGAGATGATGGATATCCTGTCTCCACTCCACCA
CCATGACACGGCACTACAGTGTGTTGCAGAGAGAGCTTACCTCAAACGACTGGAAGGTGGGTGTACAGTGCCTGTATGTG
TCAACGCG
>Calliostoma_zizyphinum_mantle_c45984_g1_i1 m.16403 c45984_g1_i1|g.16403 ORF
c45984_g1_i1|g.16403 c45984_g1_i1|m.16403 type:internal len:228 (+)
c45984_g1_i1:2-682(+)
CATCTCTCCGCTCCAGGAGCAGTTGAAAAACCGCCTCGCTCGTCTGCATGATAAAGAGGCTGGCCTCATTTTCACCTCAT
GCTATGTCGCAAATGACTCGACACTCTTCACACTACTCAAAGCTCTGCCAGGATGTCACATCTTCTCCGATGCGGGGAAC
CATGCCTCCATGATAGCAGGAATCCGAACAAGTGGAGCCCCCAAACATGTATTCCGTCATAACGACCCTGAACATCTCGA
GGAACTACTGAAGAAGGTGGACGTCAATGTGCCTAAAATTGTTGCCTTTGAAACTGTACATTCCATGGATGGTTCCATCT
GTCCTCTGACGGAAATGTGCGATGTTGCCCACAAGTATGGCGCCCTGACCTTTGTGGATGAAGTCCATGCTGTTGGTCTC
TATGGTAACCATGGTGCTGGAATCGCAGAGAGAGATGGATGTCCGGAGAAAGTTGACATGATCTCAGGAACTCTAGGCAA
GGCATTTGGCAATATGGGAGGCTATGTTGTGGGAAGCGCCAACACCATTGATATGATACGAAGTTATGCTGCCGGGTTCA
TCTTCACCACTGCCCTTCCTCCAACAACACTCAGTGGAGCGCTGGCTTCCATTGATTTATTGGCTGGTGACGAAGGACGT
GAACTTCGAGCCAGACATCAGAACAATGTGCAGTACCCACGAG
>Calliostoma_zizyphinum_mantle_c70732_g1_i1 m.24082 c70732_g1_i1|g.24082 ORF
c70732_g1_i1|g.24082 c70732_g1_i1|m.24082 type:5prime_partial len:166 (-)
c70732_g1_i1:4-501(-)
TTCAAGGTAAACTTTGCCACAAAATGGAAGAGTTGGATGGCCAAGCTCGTTTCAGGGTTGACAAATGGGACCGTAAAGCA
GGTGGCGGTGGCATATCTTGTGTAATGGAGAACGGTGCAGTTTTCGAAAAGGCTGGCATTAACATTAGCGTCATCCACGG
AACACTCAGTCCTAATGCTGTCAAGGAAATGCGTGCCAGGCACAAAGAGATTGATGTCGACAAAGATTGCAAATTCTTTG
CCTGTGGTATCAGTTCAGTCATTCATCCTTTAAATCCCTACGTGCCCACTACGCACTTCAACTTCCGCTATTTTGAGGTA
GACATGGGGGCTGGTCGCAAGTTTTGGTGGTACGGTGGCGGTTCAGACTTAACTCCGTATTATCTGTTTGAAGACGATGC
CAAGCACTTCCACGAGCAATTCAAGCAAGCCTGCGACAAACACAACTCATCCTATTACAGTCGGTTTAAGCAATGGTGTG
ATGATTACTTTTTTCTGTAGCAT
>Calliostoma_zizyphinum_mantle_c75662_g1_i1 m.10632 c75662_g1_i1|g.10632 ORF
c75662_g1_i1|g.10632 c75662_g1_i1|m.10632 type:3prime_partial len:275 (-)
c75662_g1_i1:3-824(-)
CAGGTAATGGTATGGTACACGGAATGTAACATGTAACTTTTGCAAGCACGAACAGATATGACATTAGGAACGTAAAAACA
TGTCTGTGGTCAAGTAATGTGCTATCTCGTCGGATATGTGATTGCTTGTCTGGTGATGGCTGAAGTCAAGAAGAGGCTGG
TTGTGTTATTTAAAGCCACCAAGGATAAGGAAGAAGACAAGTTTGCCAAGGAACTGAAAAAGTGCGGCTATGAAGCAGTA
AGTATCCCTGTTTTGACATTTACATTTGTCAATGTGGACAAGCTAAAGTCTCACCTGGATAATCTGGACACATTCTCAGC
TATCATCCTGACAAGTCCCCGAGCAGTGGAGGCCGTTAACAAAGCTGCAGAAGGAACAGCAGACAAAGCCAAGAGATTCA
GCTCGATCAAGTGCTATGTTGTTGGTAAAGCCACTGGTATTTTAGCCAAAAAGGCTGGATTCACAACAACAGGAGAGGAA
TCAGGGAGTGCTGCTGAGCTTGCCAAACTCATTTCAGATGGTTCAACTGCAGAAACCAAGCCATTCTTGTATCCATGTGC
TAACATACGTCGTGATGTCTTGGTGGAACACTTGAAAGTAAAAGAGCTCAAAATTGAGGAGGTGACAGCCTATGAGACCA
ACCCCAACGCCAGTCTGGAGAATTCTGTGATTGAGATGCTTGACAAACAGGGATTGCCCGAGTTTGCAGTATTCTTCAGC
CCATCTGGCGTGCAGTACACAGAGCCATTAGTCAAGAAAGGAGTGCTACCAATGGACAAGATCAAGGTTATAGCGCTGGG
ACCGGCCACAAAGTTGGAACTAGAGTCGAGGGACTTCAAACTCCATGGTATCACAGCCAAACCAGAGCCTGTCAGTCTGG
TCACAGTTTTAATACAAGAGAAGGCGGAAACAGAGGGAGA

>Calliostoma_zizyphinum_mantle_c79246_g1_i1 m.32213 c79246_g1_i1|g.32213 ORF c79246_g1_i1|g.32213 c79246_g1_i1|m.32213 type:internal len:130 (+) c79246_g1_i1:2-388(+)
ATTGGCGTTGCTTACTGATGCTGTTGTCACTCATCTGGTCTGCCAAGCCGTCGCAGGAGCTCAGTTGTTACAAGTATTCG
AATCGCACGCTGGTGTCCTCACACCTGCTCTTTTCAACGCATTTGCTCTACCTTATCTCAAGCAAATTGTTGGTCAACTG
CGAAAAGAATTAGTTGAAAGGCATGGTTATACCAGCTCCGATTTGCCGCCCGTTATTATATTTGCAAAAGACGGCCATTT
TGCCATCGACCAGTTGGTGAATGATGCGGGCTACGATGTAGTCAGTTTAGATTGGACCATTTCGCTAGACCAAGCACAAG
ATGTAGCTAATAACAAAGTCACTCTTCAGGGAAATCTGGATCCCTGTGCTCTGTATGCACCCAACGATG
>Calliostoma_zizyphinum_mantle_c8094_g1_i1 m.29755 c8094_g1_i1|g.29755 ORF c8094_g1_i1|g.29755 c8094_g1_i1|m.29755 type:5prime_partial len:117 (+) c8094_g1_i1:2-352(+)
GTGCTACCAGTTGCCCCCTGGGTCAATCGGCCTGGCAGAAAGAGCAGTTGATCGAGATGTGGCTGAAGGGGCAGACATGC
TGATGGTCAAACCAGGCCTCGCTTACCTGGATGTGGTCAGAATGACCAAACAAAAGTACCCGACCCACCCTCTCGCTATC
TACCATGTATCTGGAGAGTATGCGATGCTGCATCACGGAGCCTCACAGGGAGCATTCGAGCTGAAGGCTGTGGTCTTGGA
GTGCCTGCAGAGCATGAGAAGAGCAGGTGCTGACATTATCATCACCTATTTCGTCCCTGATCTGCTAAACTGGTTGAACA
CATCCGAGAAACAGGCCAAGGAAGTTGCTTGATTGGAAGATGGAGTTGATAATCCTGCATAAAAAAAAGTTCAACAGTTC
ATGGGCATTTTTTTGAGTGA
>Calliostoma_zizyphinum_mantle_c90431_g1_i1 m.33052 c90431_g1_i1|g.33052 ORF c90431_g1_i1|g.33052 c90431_g1_i1|m.33052 type:internal len:126 (-) c90431_g1_i1:3-377(-)
CCGCAAGTGGTTCCCTCAACTCCTGGTTGCATGTGATGTTTGTCTGTGTCCCTACACATGTCACGGACACTGTGGTATTC
TGTGGGAAGATGGAACGCTCGACAACGCAGCCAGTATAGCAAGACTAGCAGACATATCTGTTGCCTACGCTAAAGCAGGT
TGCCAGGTGATTGCTCCATCAGATATGATGGATGGTAGAATAGGAGCCATCAAGAAAGGACTACATGCAGCTGGTCTAGC
CAACAAGGTGGCGGTGCTGAGCTACAGCGCCAAGTTTGCATCCAGTTTTTATGGACCTTTCAGAGATGCTGCAAAAAGTG
CCCCATCATTCGGAGACAGGAAGTGCTACCAGTTGCCCCCTGGGTCGATCGGACTGGC
>Clanculus_pharaonius_mantle_c13026_g1_i1 m.8590 c13026_g1_i1|g.8590 ORF c13026_g1_i1|g.8590 c13026_g1_i1|m.8590 type:3prime_partial len:334 (-) c13026_g1_i1:1-999(-)
CTGCCTGTCAATGTGTCACCTGTAGCCAGGTCTATCAAATACACATCTGTTTCCCGCTGTGAGAACAATTAACATACTCG
GCAACAATGAAGCGACTTGCGTGCCCATTTCTCACCAAGATACCGGTGAACAACTTGCGACAGTTTCCACAGCAGCTGCT
GTCCTTTGCCAACCGGTGTCCGGTGATGAACCATGCTGTGCAGTACTCCTCCAGTGTGGCTGTCAGCCAGGACAGTCCCA
GCTTGGAGGAGGGGTCGAGGTGCCCATACCTGGCCAATGAGATGACAGTTCAGGAAGCCAGTCCAGCCATCCAACAAGAT
GTCATTGCTGTTAGTAGGCAAGAGGACCATCTGCTTCTGGCCCAGGAGAGTGCTGGTCTGAGTGACACTGTGGCAGAACA
TGCAGCCTTCACTGCCGCCTCGGACAGCGTGACAGTTATGGCAGGAGATACTGGAGTGGAAAAGAGTTCCAACTCAGGGG
GGATGCAGGGCTGGAACCTGAGTGAGACCCTCAAGATGCTGAAGAAATCTGGATCAGGGCCCAGTGGAGGTAAACCTCAG
GATCTGTTCAACTATGAAGCCTTCTTTGCGAATGAAGTTGACAAGAAGAAAAAGGACCATTCCTATCGTATTTTCAAGAA
AGTGATGCGCAATGCAAGTACCTTTCCATTTGCTCGGGAACATTCAGACGGCGAGATGGACATCTCTGTATGGTGTAGTA
ACGACTACCTGGGGATGAGCTGGCACCCAGAGGTCAGGGAGGCTGTAAGGACTGCCCTTGACAAGCACGGAGCTGGTGCT
GGCGGCACAAGGAACATCTCGGGGAACTCACCACTCCATGAACGTCTAGAACAGCGTCTTGCTCGTTTGCATGACAAAGA
GGCTGGCCTCATCTTCACCTCATGTTATGTTGCCAACGACTCAACTCTGTATACTCTACTCAAAGCACTTCCAGGCTGCC
ATATCTTCTCGGACGCGGGTAACCATGCCTCCATGATTGCTGGTATCAGAACAAGTGGAGTACCGAAGCACATCTTCCGT
CACAACGACCCCGATCACCTTGAGGAACAACTCAAGAAGGTGGAT
>Clanculus_pharaonius_mantle_c16373_g1_i1 m.8032 c16373_g1_i1|g.8032 ORF c16373_g1_i1|g.8032 c16373_g1_i1|m.8032 type:5prime_partial len:307 (+) c16373_g1_i1:3-923(+)
GTAACGCAGCTCTTTGGTCACTTGAACTGGAGAACTCTCTGAAGGCCGGTAAAGTGGACATGATTGTCCACTCTTTGAAA
GATGTCGCTATATCCCTTCCTGATGATCTCGTCCTAGGGTGTATACTTAAGCGAGCAAACCCTCACGATGCCATTGTCAT
GACCGCACAGAATCAGGGAAAAACATTAGCAACGTTGCCAAAAGGAAGTGTTGTAGGCACCAGTTCTTTACGGAGAGCCG
CTCAGATCAAGAGGAATTACCCACACCTACAGATATTTGACATCCGAGGTAATATTGATGGCCGAATCAAGAACCTAGAT
GAGAGCGGACAGTATGATGCACTGTGTCTAGCTGCCGCCGCACTCGAAAGAATGGGCCCCGAATATGAGAAGAGAACATC
ACAGATCCTGTCTGCTAAAGACTGCATGTACTGTGTGAGTCAGGCCGCCCTGGCGGTGGAGTGTCGAGCAGACGACACGG
AGGTTCTGGATATCCTGGATCCATTCCATGACAGGAATACGGTCATCAGAGTCGTCGCCGAGAGGGCGTACCTCGGGAGG
CTGATGGGCGGATGCAGCGAACCAATAGGGACCGAGTCGACTCTGGAGAACAACACTCTGACACTGAAGGGAGGAGTTTT
TAACGCCGAGGGAGACGAAGCAATACTGGACGAAGAGACACGAGTCCTGCCGGGAAATATCAAAGATATATATCAGGCAC
CAAGCGGACCTCAAAAGCACTACGCGTCTATTGTGACAGGAAAGAGAATCAGTCCTGAGACGCTGCAGGCTGCGGAGAAA
CTGGGACTGGATCTAGGGGATAGGATGGCCAAGAAGGGGGCGGAGAAGATACTGGACACTGTCCGAAAAGAGATCGCAGC
ATCCAAAGATGCTAACAATGCAGCAAAGAATGGACACCTCTGACTTGAACTTGTAGTCTAAGTGTACTGACCCTGTTAGC
CGGAGATTTTATATTAGTTAAGGATATTTTGTAGTTTTGTTTCACCCAGCCTGTAATGACACATTATGGCTATGCTGGTA
ATCGTTTTCAGGAAAGTTAGTATACAAAGTACAAGTTTGAATTGGCGATATATACAAACGAAGTATTTTACAGCTTGTTT
AGTGTTTGTATGGGTTTGAAA

>Clanculus_pharaonius_mantle_c18584_g1_i1 m.3973 c18584_g1_i1|g.3973 ORF c18584_g1_i1|g.3973 c18584_g1_i1|m.3973 type:complete len:370 (+) c18584_g1_i1:265-1374(+)
GACAGTACAATAGCCACAGCAATACTACACGTGGCTGGAAGTGAGTGGTTGATTGAAAACCTTTGCAGTATCCTGCTGTA
CTACACGAGGGCTCCGGACTCAGCGAGTGTTTGATATAACACTATACTTATTGACAACCAGTTTGATTCAAATTTTAAAG
GAGGCGGAAGTCGTTGCGGAGAGTTTCTACGTGGCAGACGACGAGGCAGCGTGACTGTGTTGTTTACATGATCAGATCCG
CACATCTAATCCGCATTAATTGAGATGTTTGCCACGAAAACTGTCAAGGTTGGATCTCGGAAGAGTAAGCTTGCACTTAT
ACAAACCAACTCTGTGATAGAGAAATTGAAAAAGATTCATCCACACTTAGAGTTTGAAGTTGTTACTATGACAACAGTTG
GTGACAGAATTCTCGATTCAGCATTATCTAAGATTGGTGAAAAGTCATTATTCACAAAGGAACTAGAGGAATCTCTGAAG
GATGAGACAGTTGATCTTGTGGTGCACTCACTGAAAGATTTACCCACAATACTTCCTGAAGGACTTGTCATAGGTTGTGT
GAATGAACGTGATGATCCCAATGATGCAGTTGTGATGCATCCAAAACACTCCGGGAAAACTCTGGATGATCTGCCAAAAG
GGAGTGTGATTGGTACCAGTTCCTTAAGACGATGTGCTCAGATAAAGAGAAACTATCCACATTTGGAAATCCAAGATATT
AGAGGTAATCTTAATACACGGTTCAAGAAACTTGATGAGGATGATGTTTATGATGCTATAATACTAGCTGTGGCTGGTCT
AGATCGAATGGAATGGTCTGATCGAATATCTCAGAAGTTGCCTGCAGATGTGTGTATGTATGCCGTTAGTCAGGGGGCCA
TCGCAGTAGAGTGTCGTGTGAGGGACAAGAACATCCTGGATATTCTGTATCCTCTGCATCACTGCGAGACAGCCCTTCGA
TGTGTGGCAGAGAGGGCATATCTCAGGAGACTGGAGGGTGGCTGTACTGTACCAGTTTCAGTTTTCACAGAGATCAAGGA
TAATAAGATGTCTATACGTGGAGGTGTGTACAGCACAGATGGTGTAGAGGCAGTTGAAGACTCACTGGAGTCAACGTTTC
TTTCTCAGGAGGAACAGCAAACTGTAACAAATGGCTCCAACACCTATTGCTCCATTGTCTGTGGCAAGGGAAACAACTCG
AAGAGTTTCCTCGTGGCAGAGAAGCTGGGCATTGACCTCGCTGACAAGATGGTGGAACAAGGTGCAGGGGAAATTTTAAG
GATAGCTAAGCGAAAAGCAAGAGATGCGATCGAGGATGAGCGGGAAAGAAAGAAACTCCGAAGTACAAATGCCAAATCGG
AAGTGTCTTCATAATACGGACATGTATGTCAGAATGAATTACAACTACATTTTAAGTCAAGGTCCCTTATCCTGCTGTGA
ATTCCATAGTGTTTGTGTGGGCTGTAACAGATTATTTAACTACCGCTTTAAACAGATTTAACTTCTGTGGGTTGATGCTA
ACCCTTTAACTGCAAGAGAAACACCTTTGTCAGTCCCTGGTTAGTCCGGGGTCAGTCCCTGTGAGCTATGCTGTGGAAAT
TGCAGCCAACATGATGCCGCTTGACCCTGCTCGGAGCTTAGGCTTAGCACTGCTCAAGAATCTCACCAGGCGGGGATTCT
TTTGCCAAACCGTAATGCTAACCGTTACCTTCCCCAATCACCCATAACTCATTTGACAAATTTGACTATATGCTTATTTG
CTTATTATGAGAAACATGAGTGGTCTAGATGTATCGGCATATACAGAAGTTTATGTTGAAACTAAAGCTCTCAGCACCAC
TCT
>Clanculus_pharaonius_mantle_c18584_g1_i4 m.4147 c18584_g1_i4|g.4147 ORF c18584_g1_i4|g.4147 c18584_g1_i4|m.4147 type:complete len:370 (+) c18584_g1_i4:217-1326(+)
CACACACACACAGGCAGAGAGGTCGCATATAGCAATCGTAACTCATGCCAGTGACTAGTCAAAATACTAGAACTCCAAAC
AGTGTCAATACCTTAGTCTCATACTACCTTAGGAGGCGGAAGTCGTTGCGGAGAGTTTCTACGTGGCAGACGACGAGGCA
GCGTGACTGTGTTGTTTACATGATCAGATCCGCACATCTAATCCGCATTAATTGAGATGTTTGCCACGAAAACTGTCAAG
GTTGGATCTCGGAAGAGTAAGCTTGCACTTATACAAACCAACTCTGTGATAGAGAAATTGAAAAAGATTCATCCACACTT
AGAGTTTGAAGTTGTTACTATGACAACAGTTGGTGACAGAATTCTCGATTCAGCATTATCTAAGATTGGTGAAAAGTCAT
TATTCACAAAGGAACTAGAGGAATCTCTGAAGGATGAGACAGTTGATCTTGTGGTGCACTCACTGAAAGATTTACCCACA
ATACTTCCTGAAGGACTTGTCATAGGTTGTGTGAATGAACGTGATGATCCCAATGATGCAGTTGTGATGCATCCAAAACA
CTCCGGGAAAACTCTGGATGATCTGCCAAAAGGGAGTGTGATTGGTACCAGTTCCTTAAGACGATGTGCTCAGATAAAGA
GAAACTATCCACATTTGGAAATCCAAGATATTAGAGGTAATCTTAATACACGGTTCAAGAAACTTGATGAGGATGATGTT
TATGATGCTATAATACTAGCTGTGGCTGGTCTAGATCGAATGGAATGGTCTGATCGAATATCTCAGAAGTTGCCTGCAGA
TGTGTGTATGTATGCCGTTAGTCAGGGGGCCATCGCAGTAGAGTGTCGTGTGAGGGACAAGAACATCCTGGATATTCTGT
ATCCTCTGCATCACTGCGAGACAGCCCTTCGATGTGTGGCAGAGAGGGCATATCTCAGGAGACTGGAGGGTGGCTGTACT
GTACCAGTTTCAGTTTTCACAGAGATCAAGGATAATAAGATGTCTATACGTGGAGGTGTGTACAGCACAGATGGTGTAGA
GGCAGTTGAAGACTCACTGGAGTCAACGTTTCTTTCTCAGGAGGAACAGCAAACTGTAACAAATGGCTCCAACACCTATT
GCTCCATTGTCTGTGGCAAGGGAAACAACTCGAAGAGTTTCCTCGTGGCAGAGAAGCTGGGCATTGACCTCGCTGACAAG
ATGGTGGAACAAGGTGCAGGGGAAATTTTAAGGATAGCTAAGCGAAAAGCAAGAGATGCGATCGAGGATGAGCGGGAAAG
AAAGAAACTCCGAAGTACAAATGCCAAATCGGAAGTGTCTTCATAATACGGACATGTATGTCAGAATGAATTACAACTAC
ATTTTAAGTCAAGGTCCCTTATCCTGCTGTGAATTCCATAGTGTTTGTGTGGGCTGTAACAGATTATTTAACTACCGCTT
TAAACAGATTTAACTTCTGTGGGTTGATGCTAACCCTTTAACTGCAAGAGAAACACCTTTGTCAGTCCCTGGTTAGTCCG
GGGTCAGTCCCTGTGAGCTATGCTGTGGAAATTGCAGCCAACATGATGCCGCTTGACCCTGCTCGGAGCTTAGGCTTAGC
ACTGCTCAAGAATCTCACCAGGCGGGGATTCTTTTGCCAAACCGTAATGCTAACCGTTACCTTCCCCAATCACCCATAAC
TCATTTGACAAATTTGACTATATGCTTATTTGCTTATTATGAGAAACATGAGTGGTCTAGATGTATCGGCATATACAGAA
GTTTATGTTGAAACTAAAGCTCTCAGCACCACTCT
>Clanculus_pharaonius_mantle_c18584_g1_i8 m.7761 c18584_g1_i8|g.7761 ORF c18584_g1_i8|g.7761 c18584_g1_i8|m.7761 type:complete len:201 (+) c18584_g1_i8:265-867(+)
GACAGTACAATAGCCACAGCAATACTACACGTGGCTGGAAGTGAGTGGTTGATTGAAAACCTTTGCAGTATCCTGCTGTA
CTACACGAGGGCTCCGGACTCAGCGAGTGTTTGATATAACACTATACTTATTGACAACCAGTTTGATTCAAATTTTAAAG
GAGGCGGAAGTCGTTGCGGAGAGTTTCTACGTGGCAGACGACGAGGCAGCGTGACTGTGTTGTTTACATGATCAGATCCG
CACATCTAATCCGCATTAATTGAGATGTTTGCCACGAAAACTGTCAAGGTTGGATCTCGGAAGAGTAAGCTTGCACTTAT

ACAAACCAACTCTGTGATAGAGAAATTGAAAAAGATTCATCCACACTTAGAGTTTGAAGTTGTTACTATGACAACAGTTG
GTGACAGAATTCTCGATTCAGCATTATCTAAGATTGGTGAAAAGTCATTATTCACAAAGGAACTAGAGGAATCTCTGAAG
GATGAGACAGTTGATCTTGTGGTGCACTCACTGAAAGATTTACCCACAATACTTCCTGAAGGACTTGTCATAGGTTGTGT
GAATGAACGTGATGATCCCAATGATGCAGTTGTGATGCATCCAAAACACTCCGGGAAAACTCTGGATGATCTGCCAAAAG
GGAGTGTGATTGGTACCAGTTCCTTAAGACGATGTGCTCAGATAAAGAGAAACTATCCACATTTGGAAATCCAAGATATT
GTATCCTTTCTCACACACCTTCCTCATGTTCCTGGTAGTTTTATTTTCACTTACCGTAGGTCTTCATGGAATTCCTTTAC
AATCACTGATGGTGTTTTTTATGAGAAATGGTGGACAAATAGTGTACAGTTGCCTTTTGCTACTTAAAATTGTTCAGAAG
TTACTTTTGGTTTTGGTCTTTCAATGGTTGTAAGTTGGGCTCATGTTGATAATCATGGAGGAAGCATTCATAGAATTATA
TACATGTATATGTATTCCTGGATCACAGAAGAGATTTTTCAAATCACTTAACGTAGACCATGTCTTCATCCTTTACAATC
ACTGATGGTGTCTTTATGAGTAATGGTGTGCATGTCTAGCAAGTAGTGTACAGGTGCCTTTTGTTACTTAAAGACTGTGT
TCGACCGTGAACTTTTGATGGATCCGAATGTCGAGTGGGGATTCCCTTTTGCA
>Clanculus_pharaonius_mantle_c18993_g1_i1 m.2959 c18993_g1_i1|g.2959 ORF
c18993_g1_i1|g.2959 c18993_g1_i1|m.2959 type:5prime_partial len:280 (-)
c18993_g1_i1:1335-2174(-)
AGGAACAACTCAAGAAGGTGGATGCCACCATCCCCAAAATTGTTGCCTTTGAGACTGTACATTCCATGGATGGATCTATC
TGCCCCCTGAGAGAAATGTGTGACGTTGCCCACAAGTACGGTGCCTTAACCTTTGTGGACGAAGTCCATGCCGTGGGTCT
GTATGGCAAACATGGAGCTGGCATTGCTGAAAGGGATGGATGTCCAGAGAAAGTTGACATTCTATCTGGAACTCTTGGAA
AGGCGTTTGGCAATATGGGAGGCTACATAGTTGGAACGTCCAATACAATTGACATGATACGAAGCTACGCCGCTGGGTTC
ATCTTCACAACAGCTCTACCCCCTACAACACTGAGTGGAGCCTTGGCATCCATTGATGTTTTGGCAAGTGATGAAGGCCG
AGATCTCCGTGCCCGACATCAGAGTAACGTCCGCTACCTACGAGATAAGTTGACTCATGAAGGAATCCCGGCCATGCACA
GCCCCAGTCACATCATACCAATACACGTTGGAGACGCTCAAAAAGCTACCCAGGTTTCAAATGATCTTATCCAGGACCAT
GGCATTTACGTTCAGGCCATCAACTATCCGACAGTCGCTCGTGGATTGGAGAGACTTCGTGTGGCCCCAACTCCTCACCA
CACCCGGGAAATGATGGATGCCTTTGTTGACAGCGTGGTCGAAACCTGGAAAGCAAATGACCTTGACCTTTACAATCCGA
TCTGCCCGAAGACATGCGAGAGCTGCAACAAACAACTCAAGCTCCAAGAGTTCTTCAAACCTGATCCTGTTTGTTCCCGC
TCCAATTGCACATACTCTTCCCTTCAGGCGACTCTTGCATAAGTGCAACAATACATGTCACGTGTTCAGTTTTTGACCTT
GACCTTAGTCCTGTAGTGACCAGGCTTTTGTATTTGTGATTCAAAAATATTCTCATACTGGCTTACTTAACCATTAACCC
TTTGGCAGACCCCTCATTTCCAGTTATATCTGGAAATATCTGTGGTCACTGAGTTGGCTACATAGTTGGCCAACATGATGC
TCAAAATAAAACTCACTTGATTGAAAATCTTAATTAGAACACCTTTTTGTGCAAACTATTTCTGAATATTGAAAAGAAAT
TAAATGTTATGATGCAAAAAATGAAGAGTGTATTAGTAATGACTGGTTATTAACCAGAATAAAAGATTCATTTATTAATA
TATAAGTATTTTGTGCACAACCTGGCTGCAGATGTGAGTTGATCTTAAGTTCAAATTATCCAATATTTGATGTAATGTCT
CTTAGAATCAGATATATCAATGTGTTCATTTCCAACAAATCTTTGATAAAAGTGTGCAATTAGTAAGTCGTATTTTGTCT
CATAAACTTTGACCTTTTATTAGAATATTTATTATTTTCTGTCAGTTTAAATTAAAGTTTTGTTTATATTTTGTGCTAAA
AAGAAGACCTCAAAAATTTGGTAACTATAAGTGTATCATTAAACAATTGTTTGTAAAGTGGTTGAAAGATTTGAAAACAA
TAAACAATAATGGCAGTCATTTTTCCGACCTTGTTGAGTCACTGATTACAGAAAATATGAACTTGCTTTGTTTCAACAGT
CATGTTTGGAATCTCTCATCTGATGAACAAACTGACATTTGGGTCAATATTGTGATTTTTTATGGGGGGGGGGGGTTTCA
TACATAGTTTAATTCTTGTTTGTTTGTTATTTATCTAGATTCTAGCAATGATTTATTTGTACATCCTTGACTCCCGTGGC
CATTGGCAAATTTGTATTTTGCAAATTTTTAATGATGTAATAATTATCTATGTAAATTCCTTTTAATTAATGAGCAAAAA
AAAAATAAAACTACAAAGGTAATAAGTTACAAATAAATTTTACAAAAATAACCAAATATACTGAAAGAACTAACTTTTAA
CTTGGCAATTACTGTATAATTATTTAAATACTGATATTTAAATAATGAAAATATAATATTGATTGATGTATTAAGTGCCC
TGATACTGTAAACACTCCTTGGCTACATTTTGGACAGATGAAGTATGTACATAAAGATATGTTCTTTGACTGATAAACAA
AGTAACAGTTGACCCTTTTAAGTGTCCTGTCCTTTGTCTGATTTCACAAAACACAACAGTAAAGAAGGGCTCTCGATGCG
TTCCCTACCCCTCAAC
>Clanculus_pharaonius_mantle_c2508_g1_i1 m.14858 c2508_g1_i1|g.14858 ORF
c2508_g1_i1|g.14858 c2508_g1_i1|m.14858 type:5prime_partial len:162 (+)
c2508_g1_i1:1-486(+)
GATATGATGGATGGAAGGATTGGAGCAATAAAGAAAGGTCTCCATGCAGCAGGACTTGGTAACAGAGTCAGTGTGATGAG
CTACAGCGCCAAGTTTGCATCGAGTTTCTATGGACCTTTCCGTGACGCAGCTAAAAGCGCCCCATCGTTCGGAGACAGAA
AGTGTTATCAACTACCTCCTGGGTCTATTGGTCTCGCGGAGAGGGCAGTGGACCGAGATGTGGCAGAGGGTGCGGACATG
TTGATGGTGAAGCCAGGCCTTGCATACTTAGATGTTGTCAGAATGACCAAACAAAAATACCCGACCCACCCCTTAGCAAT
CTATCACGTGTCTGGAGAGTATGCTATGTTGCATCATGGCGCCAAGCAGGGGGCGTTTGAGTTGAAGGCGGTAGTCCTAG
AGTGTTTGCAGAGTATGAGACGGGCAGGTGCAGATATAATTATCACCTACTTTGTTCCTGATCTCCTGGACTGGATCAAG
TCATAGACTCTCATGGTCGCAGTCGCCAATACACTTTGAAAGTTGTCTACAATCATGTTGGTCATCAAAAATAATATTTC
AAACAACAAAACAACAAACTACCTTTTGAGTGCTGAATATTAGTTTTGTAAGGGCATATTGCTAGCCTATTTTGTGGCGA
CTTGTTTAAAGAGCTTGTAATCAGCTCATT
>Clanculus_pharaonius_mantle_c36956_g1_i1 m.30373 c36956_g1_i1|g.30373 ORF
c36956_g1_i1|g.30373 c36956_g1_i1|m.30373 type:internal len:101 (+)
c36956_g1_i1:3-302(+)
AATTGGCAAGGCAGGAAATTGCTGAGTACGAGACCAACTCAAATGAAAATTTGGAGAGGAGCTTAAAGGAAATGCTGGAG
AAGCAGGGAATTCCTGAATTTGCAGTGTTCTTCAGTCCCTCAGGTGTACAATACACGGAGCCGCTTGTGAAGAAAGGTGT
ACTGCCAATGGATAAAATGAAGGTGATAGCTTTGGGTCCTGCAACCAAGTTGGAGGTGGAGTCTCGAGGGTTCAAACTAC

ACGGTGTTACAGCCAAACCAGAACCAGCCAGTCTCATCACCGCCCTCAGCGAGGAGAAACCGG
>Clanculus_pharaonius_mantle_c57098_g1_i1 m.27422 c57098_g1_i1|g.27422 ORF c57098_g1_i1|g.27422 c57098_g1_i1|m.27422 type:internal len:115 (-) c57098_g1_i1:1-342(-)
TGACTGAAGGAACTCAACCAAGAATGGCCAGAGTAATATCACAGTGTTTGCACACCCTCCAGAGTCTTACAGTTGTCACA
GTAACAAGAATTTCACCACGCATCAAGAAACATAGGTATGGTTTCCTTGGATTTGTGATTGGTGGATCTGCATACTATCA
GACAAATGGCAAATCAAAGGTTTATGCCGAGGCCTTTGATGCAAGGTCTCAGAGATCAAAGTGGATGGCAGACCCAGTCA
CTGACATGGAGGAGTTACAAAAGAATTCCTCTGACATGAGAAGCAGAATGGAAATGCTTATAATGCGAATACAGGGTGAA
GTATGCAGAGCTCTTGAAGATAGC
>Clanculus_pharaonius_mantle_c6567_g1_i1 m.30022 c6567_g1_i1|g.30022 ORF c6567_g1_i1|g.30022 c6567_g1_i1|m.30022 type:internal len:103 (+) c6567_g1_i1:2-307(+)
GCCAGAGTATGGTCAAGCAAAAGGTGATAAAGCTTTCTTTGCTACATGTCGAGACAAAGAGCTTGTCAGTGAACTTACGT
TGCAGCCTATTGACAGGTTTCCCCTTGATGGTGCTATCATCTTCTCCGACATACTGGTTATCCCCCTTGCCTTGGGATTA
ACTGCTCTGAATGATCCAGGAAAGGGTCCTGTGTTTGCTGATCCCATTGAGAAGCCAGAGGATGTTGATCGCCTAAATCC
CAGCTTTGATATTCACAAGGAGCTGGGTTACGTTTATGATGCAATCAATCTCACACGACACAAACTG
>Clanculus_pharaonius_mantle_c70866_g1_i1 m.24442 c70866_g1_i1|g.24442 ORF c70866_g1_i1|g.24442 c70866_g1_i1|m.24442 type:internal len:133 (-) c70866_g1_i1:2-397(-)
CGGCGGAGGTTCCCCCATCAAGCGCTGGACAGAACTGCAGGGTCAGGGAATGGTGGAACTTTTAGATAAAATCAGCCCTG
AGACTGCGCCACATAAACTCTATGTTGGTTTTCGTTACGCACATCCACTTACTGAGGATGCCATAGATCAGATGGAACAA
GATGGAATTGAGAGAGCAGTTGCTTTCACACAGTACCCACAATACAGCTGTTCTACAACAGGAAGTAGTTTAAATGCAAT
CTACCGACATTATTCAAACCGTCCCGGCCCAAGCAACTTGGTTTGGAGTGTGATAGATAGATGGCCGACACATGCAGGAC
TTGTTAAGGCTTTCGCACAAAGCATCAGGGAGGAAATTGCCAGGTTTCCAGAGGCAAGATCCGGAAGAGCACACGTCT
>Clanculus_pharaonius_mantle_c79862_g1_i1 m.28513 c79862_g1_i1|g.28513 ORF c79862_g1_i1|g.28513 c79862_g1_i1|m.28513 type:internal len:109 (+) c79862_g1_i1:3-326(+)
GCACGATAGCGAGACCCAGCGTCATATTGACGTGTGTTTACAAACCCTTGCTTAGGTCAGTCCTGATTGAAGTCATGGCG
GATCACGCAGTTCTTCACAGTGGCTATCACCATCCGGTTCTACGGGCGTGGAATTCAGTCAACACTTCTATAACGCCAGA
CAACCTCATATACCCGCTATTTATCGTTGACGAGGAGGATGCAGTTCAAGAAATACCCAGCATGCCCGGCCAGTCCAGAT
ATGGCGTCAAGCGCCTACAAGAAGCCATTGAACCACTTGTTAAGAAAGGGTTGAAAACTGTACTACTTTTTGGTGTTCCA
GGGAAGA
>Clanculus_pharaonius_mantle_c81961_g1_i1 m.19892 c81961_g1_i1|g.19892 ORF c81961_g1_i1|g.19892 c81961_g1_i1|m.19892 type:internal len:168 (+) c81961_g1_i1:2-502(+)
GACAGGTGCAGTAGTGTGCCATCTAGTGGAACAAGTCAGGGCTGGAGCACAGATTCTTCAAGTCTTTGATTCTAATGGTG
GTGAGTTAGGACCAAATCTGTTTACAAAATATGAACTGCCGTGTCTGCAGGAAATAGCTTACAAGGTCAAGATGGAGGTG
AAACAACAAGAACTTGAACTTGTACCCATGGTTGTTTTTGCAAAGGACGCCCATTTTGCCACAGAGCAGCTGGCTGGTAT
AGGCTATGATGTTGTACAAATAGACTGGACTCAAAGTCCAACACATGCAAGACGTCTGACTGGTTCGAAAGTCACACTGC
AGGGAAATTTGGATCCAGTGAATTTATTTGGAACTGATGAAGAGATTCGTCACCAAGTAAAGGAGATGGTTCAGAAGTTC
GGGACGCAGCGCTACATTGCAACTCTAGGTCACGGTGTGATGAAAGACACAAACCCAGATAAACTGGGCGTGTTCATCGA
TGCCGTCCACAAATACTCGAGG

>Uros_hit_Clanculus_margaritarius_1_mantle_c68027_g1_i1
GSATKLAKLISDGPDQAGKPYLYPCSNIRRDVLMDHMKEKELAVQEITAYETNPNENLESSLKEMLDKQGIPEFAVFFSP
AGVQYTEPLVKKGVLPMDKMKVIALGPATKMEVESRGFKLHGVTAKPEPASLLSALKE
>Uros_hit_Calliostoma_zizyphinum_mantle_c75662_g1_i1
MCYLVGYVIACLVMAEVKKRLVVLFKATKDKEEDKFAKELKKCGYEAVSIPVLTFTFVNVDKLKSHLDNLDTFSAIILTS
PRAVEAVNKAAEGTADKAKRFSSIKCYVVGKATGILAKKAGFTTTGEESGSAAELAKLISDGSTAETKPFLYPCANIRRD
VLVEHLKVKELKIEEVTAYETNPNASLENSVIEMLDKQGLPEFAVFFSPSGVQYTEPLVKKGVLPMDKIKVIALGPATKL
ELESRDFKLHGITAKPEPVSLVTVLIQEKAETEG
>Uros_hit_Clanculus_pharaonius_mantle_c36956_g1_i1
LARQEIAEYETNSNENLERSLKEMLEKQGIPEFAVFFSPSGVQYTEPLVKKGVLPMDKMKVIALGPATKLEVESRGFKLH
GVTAKPEPASLITALSEEKP
>Ppox_hit_Calliostoma_zizyphinum_mantle_c22534_g1_i1
MTMSTAVVLGGGVSGLAAVYYLQKFAGNKFAKIILLEASDRLGGWVQSTRCDNGAVFERGPRSLRPVGDSGINTLLLVEE
LGLSRNVLPCLKSSAPAKNRFLYLNKQLHTLPNSFKSVVTTQTPFSKPVLLTALKEMITKRADNSDETVHSFISRRFGCE
LADYAVDPLCRGIFAGDCRVLSMDACFPMLTAW
>Ppox_hit_Calliostoma_zizyphinum_mantle_c25737_g1_i1
TVMEREHGSVIRGMLIPKKKSIPSSALISKSRSESWASWSLSTGMQQLTDTMADVIGQDKRSEVRRLSPCTGVKIQHDGK
IKISTEGEDILADHVFSSLYAKCLGSLLPHSPLHKGLMSISAVSVAVVNLEYQGHLLPVEGFGHLLPSMESGPVLGVVYD
SCAFPEHNRKDMAASTRLTVMLGGAWFDELVALTNLDKSRVEQLAIDTVRQHLNITSSPCFTQVALQKDCIPQYIVGHNR
NLAVLEKYLADEKIPLTLIGSSYRGPSINDCINNTRLAVEKTCVT*
>Alas_hit_Clanculus_margaritarius_1_mantle_c37020_g1_i1
MKPKQVYSAEESELFKAQGKVMENHVIQKDVDSCKENGYAGQNYSPHIRENGHMTNNLFSHSKENGHVMNKLAEVSSQNG
QMVNKLSIDNGYVKNTENRHVSNTLCPQHTKNGHVRSLSNARPTQNGHLPQKSDEFVESFEETPLLVAILTYFSYGILVI
VGHIRDFLRYHGFEKVKTCTEPKLPGFVPLYASWESFFTRNLYRRVRDCWNRPIGSVAGAHMDLVERRTPDYGWNFEMTG
TKRRVMNFGSYNYLGFSENEGPCTDVVEVTTREEGVGVCAARQELGYFDIHRELDEMTAEFLGVEAAVSFPMGFATNSMN
MPCLVSKGCLILSDELNHASLVLGARLSGAGIKIYKHNNMKDLEKKLREAIVQGQPRTHRPWKKILIVVEGVYSMEGSIV
RLPEIIALKKKYKAYLYLDEAHSIGAMGPHGRGVVDYFGLDPRDVDILMGTFTKSFGAAGGYIAGTKQLINHLRVHSHSA
IYACSISPPVARQIIHSMKTIMGRDGTNLGRQRILQLAWNIQYFRKGLQKRGFIVYGNKDSPVVPLLIYLPAKTCAFSRM
CSERGLGIVVVGFPATPIIESRARFCLSAAHTKDMLDKALAIIDEVGDLLFIKYSRLTPPPSTHEEELEKQKSSVSNHIQ
*
>Alas_hit_Clanculus_margaritarius_1_mantle_c37760_g1_i1
AGFIFTTALPPTTLSGALASIDVLASDEGRDLRAQHQSNVRYLRDKLTHEGIPAMHSPSHIIPIHVGDALKATKVSNDLI
QDHGIYVQAINYPTVARGLERLRVAPTPHHTREMMDAFVDSVVDTWKANDLDLYKPICPKTCESCNKQLKLQEFFKPDPV
CSRSNCTYSSLQATLA*
>Alas_hit_Clanculus_margaritarius_1_mantle_c37760_g1_i2
EMCDVAHKYGALTFVDEVHAVGLYGKHGAGIAERDGCPEKVDILSGTLGKAFGNMGGYIVGTSNTIDMIRSYAAGFIFTT
ALPPTTLSGALASIDVLASDEGRDLRAQHQSNVRYLRDKLTHEGIPAMHSPSHIIPIHVGDALKATKVSNDLIQDHGIYV
QAINYPTVARGLERLRVAPTPHHTREMMDAFVDSVVDTWKANDLDLYKPICPKTCESCNKQLKLQEFFKPDPVCSRSNCT
YSSLQATLA*
>Alas_hit_Clanculus_margaritarius_1_mantle_c37760_g2_i1
HWNAVRTALDKHGAGAGGTRNISGNSPLHERLEQRLARLHDKEAGLIFTSCYVANDSTLFTLLRALPGCHIFSDAGNHAS
MIAGIRTSGVPKHIFRHNDPDHLEEQLKKVDATIPKIVAFETVHSMDG
>Alas_hit_Clanculus_margaritarius_1_foot_c32922_g1_i1
MKRLACPFLTKIPVNNLRQFPQQLLSFANRCPVMNHAVQYSSSVAASQDSPNMGEGLKCPYLANEMTVQEASPAIQQDVI
AVNRQEDNLLLAQESSGLSNTVAEHAASTAASDSVTVMTGDTGVEKSSNSGGMQGWNLSETLKMLKKSGSGPSGGKPQDL
FNYEAFFASEVDKKKRDHSYRIFKKVMRNASTFPFAQEHSEGQMDISVWCSNDYLGMSWHPEVRNAVRTALDKHGAGAGG
TRNISGNSPLHERLEQRLARLHDKEAGLIFTSCYVANDSTLFTL
>Alas_hit_Clanculus_margaritarius_1_foot_c45104_g1_i1
VIVGHIRDFLRYHGFEKVKTCTEPKLPGFVPLYASWESFFTRNLYRRVRDCWNRPIGSVAGAHMDLVERRTPDYGWNFEM
TGTKRRVMNFGSYNYLGFSENEGPCTDVVEVTTREEGVGVCAARQELGYFDIHRELDEMTAEFLGVEAAVSFPMGFATNS
MNMPCLVSKGCLILSDELNHASLVLGARLSGAGIKIYKHNNMKDLEKKLREAIVQGQPRTHRPWKKILIVVEGVYSMEGS
IVRLPEIIALKKKYKAYLYLDEAHSIGAMGPHGRGVVDYFGLDPRDVDILMGTFTKSFGAAGGYIAGTKQLINHLRVHSH
SAIYACSISPPVARQIIHSMKTIMGRDGTNLGRQRIQQLAWNIQYFRKGLQKRGFIVYGNKDSPVVPLLIYLPAKTCAFS
RMCSERGLGIVVVGFPATPIIESRARFCLSAAHTKDMLDKALAIIDEVGDLLFIKYSRLTPPPSTHEEELEKQKSSVSNH
IQ*
>Alas_hit_Clanculus_margaritarius_1_foot_c45732_g2_i1
GIRTSGVPKHIFRHNDPDHLEEQLKKVDATIPKIVAFETVHSMDGSICPLREMCDVAHKYGALTFVDEVHAVGLYGKHGA
GIAERDGCPEKVDILSGTLGKAFGNMGGYIVGTSNTIDMIRSYAAGFIFTTALPPTTLSGALASIDVLASDEGRDLRAQH

QSNVRYLRDKLTHEGIPAMHSPSHIIPIHVGDALKATKVSNDLIQDHGIYVQAINYPTVARGLERLRVAPTPHHTREMMD
AFVDSVVDTWKANDLDLYKPICPKTCESCNKQLKLQEFFKPDPVCSRSNCTYSSLQATLA*
>Alas_hit_Clanculus_margaritarius_2_mantle_c20663_g1_i2
MKRLACPFLTKIPVNNLRQFPQQLLSFANRCPVMNHAVHYSSSVAASQDSPNMGEGLKCPYLANEMTVQEASPAIQQDVI
AVNRQEDSMFLAQESSGLSNTVAEHAASTAASDSVTVMTGDTGVEKSSNSGGMQGWNFSETLKMLKKSGSGPSGGKPQDL
FNYEAFFASEVDKKKRDHSYRIFKKVMRNASTFPFAQEHSEGQMDISVWCSNDYLGMSWHPEVRNAVRTALDKHGAGAGG
TRNISGNSPLHERLEQRLARLHDKEAGLIFTSCYVANDSTLFTLLRALPGCHIFSDAGNHASMIAGIRTSGVPKHIFRHN
DPDHLEEQLKKVDATIPKIVAFETVHSMDGSICPLREMCDVAHKYGALTFVDEVHAVGLYGKHGAGIAERDGCPEKVDIL
SGTLGKAFGNMGGYIVGTSNTIDMIRSYAAGFIFTTALPPTTLSGALASIDVLASDEGRDLRAQHQSNVRYLRDKLTHEG
IPAMHSPSHIIPIHVGDALKATKVSNDLIQDHGIYVQAINYPTVARGLERLRVAPTPHHTREMMDAFVDSVVDTWKANDL
DLYKPICPKTCESCNKQLKLQEFFKPDPVCSRSNCTYSSLQATLA*
>Alas_hit_Calliostoma_zizyphinum_mantle_c45984_g1_i1
ISPLQEQLKNRLARLHDKEAGLIFTSCYVANDSTLFTLLKALPGCHIFSDAGNHASMIAGIRTSGAPKHVFRHNDPEHLE
ELLKKVDVNVPKIVAFETVHSMDGSICPLTEMCDVAHKYGALTFVDEVHAVGLYGNHGAGIAERDGCPEKVDMISGTLGK
AFGNMGGYVVGSANTIDMIRSYAAGFIFTTALPPTTLSGALASIDLLAGDEGRELRARHQNNVQYPR
>Alas_hit_Clanculus_pharaonius_mantle_c13026_g1_i1
MKRLACPFLTKIPVNNLRQFPQQLLSFANRCPVMNHAVQYSSSVAVSQDSPSLEEGSRCPYLANEMTVQEASPAIQQDVI
AVSRQEDHLLLAQESAGLSDTVAEHAAFTAASDSVTVMAGDTGVEKSSNSGGMQGWNLSETLKMLKKSGSGPSGGKPQDL
FNYEAFFANEVDKKKKDHSYRIFKKVMRNASTFPFAREHSDGEMDISVWCSNDYLGMSWHPEVREAVRTALDKHGAGAGG
TRNISGNSPLHERLEQRLARLHDKEAGLIFTSCYVANDSTLYTLLKALPGCHIFSDAGNHASMIAGIRTSGVPKHIFRHN
DPDHLEEQLKKVD
>Alas_hit_Clanculus_pharaonius_mantle_c18993_g1_i1
EQLKKVDATIPKIVAFETVHSMDGSICPLREMCDVAHKYGALTFVDEVHAVGLYGKHGAGIAERDGCPEKVDILSGTLGK
AFGNMGGYIVGTSNTIDMIRSYAAGFIFTTALPPTTLSGALASIDVLASDEGRDLRARHQSNVRYLRDKLTHEGIPAMHS
PSHIIPIHVGDAQKATQVSNDLIQDHGIYVQAINYPTVARGLERLRVAPTPHHTREMMDAFVDSVVETWKANDLDLYNPI
CPKTCESCNKQLKLQEFFKPDPVCSRSNCTYSSLQATLA*
>Cpox_hit_Calliostoma_zizyphinum_mantle_c25229_g1_i1
GLVTRMAGVISQCVHTLHNLTVVTVTRLSPHLKKHRYSFLGLIVGGTAYYQTHGKSKAYAQAMDIQSKRSQWMAESITDK
DQLQKNSSDMRHRMEMMIMRIQGEVCRALEEADGEKKFMVDRR
>Cpox_hit_Calliostoma_zizyphinum_mantle_c29010_g1_i1
RRWEREEGGGGISCVMQDATVFEKAGVNISIVHGNLPPSAVQQMKSRGKLLEGSSLPFFAAGISAVIHPKNPHIPTVHFN
YRYFEVETKTGKQWWFGGGTDLTPNYLVEEDVVNFHKTLKAACDKHNKSYYGRFKKWCDNYFFIKHRGESRGVGGIFFDD
IDEPNPDAAFKFVTSCAESVVPSYLPIVLKHKFDGYSYDERKWQLLRRGRYVEFNLIYDRGTKFGLYTPGARYESILMSL
PLNARWEYCHSPTPGSKEAKLTEVLRNPRDWV*
>Cpox_hit_Calliostoma_zizyphinum_mantle_c70732_g1_i1
QGKLCHKMEELDGQARFRVDKWDRKAGGGGISCVMENGAVFEKAGINISVIHGTLSPNAVKEMRARHKEIDVDKDCKFFA
CGISSVIHPLNPYVPTTHFNFRYFEVDMGAGRKFWWYGGGSDLTPYYLFEDDAKHFHEQFKQACDKHNSSYYSRFKQWCD
DYFFL*
>Cpox_hit_Clanculus_pharaonius_mantle_c57098_g1_i1
TEGTQPRMARVISQCLHTLQSLTVVTVTRISPRIKKHRYGFLGFVIGGSAYYQTNGKSKVYAEAFDARSQRSKWMADPVT
DMEELQKNSSDMRSRMEMLIMRIQGEVCRALEDS
>Fech_hit_Clanculus_margaritarius_1_mantle_c87276_g1_i1
GHSHAYRLVWQSKVGPLPWLSPQTDDAIKGLVARGRKNLLLVPIAFTSDHIETLFELDYEYAQNLGAEVGVKNIRRAASL
NDNPVFIEALADLVKTHLHTQQVCTPQLLLRCPMCTNPVCGLAKEFFRDQQPILDTLRAEDEELKLGAKS*
>Fech_hit_Clanculus_margaritarius_2_mantle_c1654_g1_i1
STEVAATVQRVMEDLGHSHAYRLVWQSKVGPLPWLSPQTDDAIKGLVARGRKNLLLVPIAFTSDHIETLFELDYEYAQNL
GAEVGVKNIRRAASLNDNPVFIEALADLVKTHLHTQQVCTPQLLLRCPMCTNLVCGLAKEFFRDQQPILDTLRAEDEELK
LGAKS*
>Fech_hit_Calliostoma_zizyphinum_mantle_c10706_g1_i1
MMLNLGGPEKTEDVHDFLLRLFLDKDLIPLPAQSKLAPIIAKRRTPSIQRQYRKIGGGSPIKRWTELQGQGMVDILDKIS
PETAPHKFYVGFRYAHPLTEDMIEQMEADGIERAIAFTQYPQYSCSTTGSSLNAIFRHYLKRTGPSNLVWSVIDRWPTHP
GLVKAFTQNIREEIAKFPN
>Fech_hit_Calliostoma_zizyphinum_mantle_c10706_g1_i2
MAKILGRRLTGLLQRSGCVRNVGVAPQVCIFDPQSYDTRHASSHSGPKTGVMMLNLGGPEKTEDVHDFLLRLFLDKDLIP
LPAQSKLAPIIAKRRTPSIQRQYRKIGGGSPIKRWTELQGQGMVDILDKISPETAPHKFYVGFRYAHPLTEDMIEQMEAD
GIERAIAFTQYPQYSCSTTGSSLNAIFRHYLKRTGPSNLVWSVIDRWPTHPGLVKAFTQNIREEIAKFPN
>Fech_hit_Clanculus_pharaonius_mantle_c70866_g1_i1
GGGSPIKRWTELQGQGMVELLDKISPETAPHKLYVGFRYAHPLTEDAIDQMEQDGIERAVAFTQYPQYSCSTTGSSLNAI
YRHYSNRPGPSNLVWSVIDRWPTHAGLVKAFAQSIREEIARFPEARSGRAHV
>Alad_hit_Clanculus_margaritarius_1_mantle_c34804_g1_i1

TISRPSVILPCVYKPLLRSVLIEVMADHAVLHSGYHHPVLRAWNSGNTSITPNNLIYPLFIVDEEDAVQEIPSMPGQSRY
GVKRLQEAIEPLVKKGLKTVLLFGVPGKIYKDNEGSGADMPTTPVIQAITLIRKWFPELLVACDVCLCPYTCHGHCGILR
EDGSLDNEASIARLATIAVNYAKAGCQVIAPSDMMDGRIGAIKKGLHAAGLGNRVSVMSYSAKFASSFYGPFRDAAKSAP
SFGDRKCYQLPPGSIGLAERAVDRDVAEGADMLMVKPGLAYLDVVRMTKQKYPTHPLAIYHVSGEYAMLHHGATQGAFEL
KAVVLECLQSMRRAGADIIITYFVPDLLDWIKS*
>Alad_hit_Clanculus_margaritarius_1_foot_c38208_g1_i1
TISRPSVILPCVYKPLLRSVLIQVMADHAVLHSGYHHPVLRAWNSGNTSITPNNLIYPLFIVDEEDAVQEIPSMPGQSRY
GIKRLQEAIEPLVKKGLKTVLLFGVPGKIYKDNEGSGADMPTTPVIQAITLIRKWFPELLVACDVCLCPYTCHGHCGILR
EDGSLDNEASIARLATIAVNYAKAGCQVIAPSDMMDGRIGAIKKGLHAAGLGNRVSVMSYSAKFASSFYGPFRDAAKSAP
SFGDRKCYQLPPGSIGLAERAVDRDVAEGADMLMVKPGLAYLDVVRMTKQKYPTHPLAIYHVSGEYAMLHHGATQGAFEL
KAVVLECMQ
>Alad_hit_Clanculus_margaritarius_2_mantle_c20500_g1_i1
MADHAVLHSGYHHPVLRAWNSGNTSVTPNNLIYPLFIVDEEDAVQEIPSMPGQSRYGVKRLQDAIEPLVKKGLKTVLLFG
VPGKIYKDNEGSGADMPTTPVIQAITLIRKWFPELLVACDVCLCPYTCHGHCGILREDGSLDNEASIARLATIAVNYAKA
GCQVIAPSDMMDGRIGAIKKGLHAAGLGNRVSVMSYSAKFASSFYGPFRDAAKSAPSFGDRKCYQLPPGSIGLAERAVDR
DVVEGADMLMVKPGLAYLDVVRMTKQKYPTHPLAIYHVSGEYAMLHHGATQGAFELKAVVLECLQSMRRAGADIIITYFV
PDLLDWIKS*
>Alad_hit_Calliostoma_zizyphinum_mantle_c8094_g1_i1
CYQLPPGSIGLAERAVDRDVAEGADMLMVKPGLAYLDVVRMTKQKYPTHPLAIYHVSGEYAMLHHGASQGAFELKAVVLE
CLQSMRRAGADIIITYFVPDLLNWLNTSEKQAKEVA*
>Alad_hit_Calliostoma_zizyphinum_mantle_c90431_g1_i1
RKWFPQLLVACDVCLCPYTCHGHCGILWEDGTLDNAASIARLADISVAYAKAGCQVIAPSDMMDGRIGAIKKGLHAAGLA
NKVAVLSYSAKFASSFYGPFRDAAKSAPSFGDRKCYQLPPGSIGL
>Alad_hit_Clanculus_pharaonius_mantle_c2508_g1_i1
DMMDGRIGAIKKGLHAAGLGNRVSVMSYSAKFASSFYGPFRDAAKSAPSFGDRKCYQLPPGSIGLAERAVDRDVAEGADM
LMVKPGLAYLDVVRMTKQKYPTHPLAIYHVSGEYAMLHHGAKQGAFELKAVVLECLQSMRRAGADIIITYFVPDLLDWIK
S*
>Alad_hit_Clanculus_pharaonius_mantle_c79862_g1_i1
TIARPSVILTCVYKPLLRSVLIEVMADHAVLHSGYHHPVLRAWNSVNTSITPDNLIYPLFIVDEEDAVQEIPSMPGQSRY
GVKRLQEAIEPLVKKGLKTVLLFGVPGK
>Pbgd_hit_Clanculus_margaritarius_1_mantle_c37610_g2_i1
MSTSTRKTLRIGSRESKLALIQSNYLISLLKEKNPDVNFETVTMTTPGDRDLSQSIAKIGNAALWSLELENSLKAGKVDL
IVHSLKDVAIHLPDGLVLGCILKRANPHDAIVMTTQNQGKTLATLPKGSVVGTSSLRRAAQIKRNYPHLQIFDIRGNIDG
RIKNLDESGQYDALCLAAAALERMGPEYEKRTSQILSAKDCMYCVSQAALAVECRADDKQVLDILDPFHDRNTVIRVVAE
RAYLGRLMGGCSEPIGTESALENNTLTLKGGVFSAEGDEAVLDEETQDLPGNIKDIIQAPSGPLKHYASIVTGSRISPET
LQAAEKLGLDLGDRMAKNGAERILDVVRKEIAASKDATKAAKNGHI*
>Pbgd_hit_Clanculus_margaritarius_1_mantle_c68853_g1_i1
TALSKIGEKSLFTRELEESLKDETVDLVVHSLKDLPTILPEGLVIGCVNERDDPNDAVVMHPKHSGKTLDDLPKGSVIGT
SSLRRCAQIKRNYPHFEIKDIRGNLNTRFKKLDEDDVYDAIILAVAGLDRMEWSDRISQKLPADVCMYAVSQGAIAVECR
V
>Pbgd_hit_Clanculus_margaritarius_1_foot_c46228_g1_i1
MSTSTRKTLRIGSRESKLALIQSNYLISLLKEKNPDVNFETVTMTTPGDRDLSQSIAKIGNAALWSLELENSLKAGKVDL
IVHSLKDVAIHLPDGFVLGCILKRANPHDAIVMTTQNQGKTLATLPKGSVVGTSSLRRAAQIKRNYPHLQIFDIRGNIDG
RIKNLDESGQYDALCLAAAALERMGPEYEKRTSQILSAKDCMYCVSQAALAVECRADDKQVLDILDPFHDRNTVIRVVAE
RAYLGRLMGGCSEPIGTESALENNTLTLKGGVFSAEGDEAVLDEETQDLPGNIKDIIQAAPSGPLKHYASIVTGSRISPE
TLQAAEKLGLDLGDRMAKNGAERILDVVRKEIAASKNATKAAKNGHI*
>Pbgd_hit_Clanculus_margaritarius_2_mantle_c20351_g1_i1
MSTSTRKTLRIGSRESKLALIQSNYLISLLKEKNPDVNFETVTMTTPGDRDLSQSIAKIGNAALWSLELENSLKAGKVDL
IVHSLKDVAINLPDGLVLGCILKRANPHDAIVMTTQNQGKTLATLPKGSVVGTSSLRRAAQIKRNYPHLQIFDIRGNIDG
RIKNLDESGQYDALCLAAAALERMGPEYEKRTSQILSAKDCMYCVSQAALAVECRADDKQVLDILDPFHDRNTVIRVVAE
RAYLGRLMGGCSEPIGTVSALENNTLTLKGGVFSAEGDEAVLDEETRDLPGNIKEIIQAPGGLLKPYASIVTGSRISPET
LQAAEKLGLDLGDRMAKNGAERILDVVRKEIAASKVATKAAKNGHI*
>Pbgd_hit_Clanculus_margaritarius_2_mantle_c20351_g1_i2
MSTSTRKTLRIGSRESKLALIQSNYLISLLKEKNPDVNFETVTMTTPGDRDLSQSIAKIGNAALWSLELENSLKAGKVDL
IVHSLKDVAINLPDGLVLGCILKRANPHDAIVMTTQNQGKTLATLPKGSVVGTSSLRRAAQIKRNYPHLQIFDIRGNIDG
RIKNLDESGQYDALCLAAAALERMGPEYEKRTSQILSAKDCMYCVSQAALAVECRADDKQVLDILDPFHDRNTVIRVVAE
RAYLGRLMGGCSEPIGTVSALENNTLTLKGGVFSAEGDEAVLDEEAQDLPGNIKDIIQAPSGPLKHYASIVTGSRISPET
LQAAEKLGLDLGDRMAKKGAERILDVVRKEIAASKDATKAAKNGHI*
>Pbgd_hit_Clanculus_margaritarius_2_mantle_c68931_g1_i1

PKGSVIGTSSLRRCAQIKRNYPHFEIKDIRGNLNTRFKKLDEDDVYDAIILAVADLDRMEWSDRISQKLPADVCMYAVSQ
GAIAVECRLRDKSILDILYPLHHRETALRCVAERAYLRRLEGGCTVPVSVFTEIKDDKMSISGGVYSTDGVQAVEDSLES
ASLSQKEQESVTNGSSTYCSIVCGKGNNSKSFLEAEKLGIDLADKMVRQGAGEILRIAKRKARDAIEDERA
>Pbgd_hit_Calliostoma_zizyphinum_mantle_c2993_g1_i1
TQDKDGRFYHHCIKMSTKKMIKVGSRKSNLALIQTNTVIDKLKKVHPHLEFDVVTMTTVGDRILDSALSKIGEKALFTRE
LEDSLLDGTVSFVVHSLKDLPTSLPDDLIIGCVNERDDPHDAVVMHKKHNYKTLDDLPEGSVVGTSSLRRCAQIRRRYPH
LEIKDIRGNLNTRFRKLDEDDVYDAIVLAVAGLDRMDWSDRISQKLPVDVCMYAVSQGAVAVECRATDTEMMDILSPLHH
HDTALQCVAERAYLKRLEGGCTVPVCVNA
>Pbgd_hit_Clanculus_pharaonius_mantle_c16373_g1_i1
NAALWSLELENSLKAGKVDMIVHSLKDVAISLPDDLVLGCILKRANPHDAIVMTAQNQGKTLATLPKGSVVGTSSLRRAA
QIKRNYPHLQIFDIRGNIDGRIKNLDESGQYDALCLAAAALERMGPEYEKRTSQILSAKDCMYCVSQAALAVECRADDTE
VLDILDPFHDRNTVIRVVAERAYLGRLMGGCSEPIGTESTLENNTLTLKGGVFNAEGDEAILDEETRVLPGNIKDIYQAP
SGPQKHYASIVTGKRISPETLQAAEKLGLDLGDRMAKKGAEKILDTVRKEIAASKDANNAAKNGHL*
>Pbgd_hit_Clanculus_pharaonius_mantle_c18584_g1_i1
MFATKTVKVGSRKSKLALIQTNSVIEKLKKIHPHLEFEVVTMTTVGDRILDSALSKIGEKSLFTKELEESLKDETVDLVV
HSLKDLPTILPEGLVIGCVNERDDPNDAVVMHPKHSGKTLDDLPKGSVIGTSSLRRCAQIKRNYPHLEIQDIRGNLNTRF
KKLDEDDVYDAIILAVAGLDRMEWSDRISQKLPADVCMYAVSQGAIAVECRVRDKNILDILYPLHHCETALRCVAERAYL
RRLEGGCTVPVSVFTEIKDNKMSIRGGVYSTDGVEAVEDSLESTFLSQEEQQTVTNGSNTYCSIVCGKGNNSKSFLVAEK
LGIDLADKMVEQGAGEILRIAKRKARDAIEDERERKKLRSTNAKSEVSS*
>Pbgd_hit_Clanculus_pharaonius_mantle_c18584_g1_i4
MFATKTVKVGSRKSKLALIQTNSVIEKLKKIHPHLEFEVVTMTTVGDRILDSALSKIGEKSLFTKELEESLKDETVDLVV
HSLKDLPTILPEGLVIGCVNERDDPNDAVVMHPKHSGKTLDDLPKGSVIGTSSLRRCAQIKRNYPHLEIQDIRGNLNTRF
KKLDEDDVYDAIILAVAGLDRMEWSDRISQKLPADVCMYAVSQGAIAVECRVRDKNILDILYPLHHCETALRCVAERAYL
RRLEGGCTVPVSVFTEIKDNKMSIRGGVYSTDGVEAVEDSLESTFLSQEEQQTVTNGSNTYCSIVCGKGNNSKSFLVAEK
LGIDLADKMVEQGAGEILRIAKRKARDAIEDERERKKLRSTNAKSEVSS*
>Pbgd_hit_Clanculus_pharaonius_mantle_c18584_g1_i8
MFATKTVKVGSRKSKLALIQTNSVIEKLKKIHPHLEFEVVTMTTVGDRILDSALSKIGEKSLFTKELEESLKDETVDLVV
HSLKDLPTILPEGLVIGCVNERDDPNDAVVMHPKHSGKTLDDLPKGSVIGTSSLRRCAQIKRNYPHLEIQDIVSFLTHLP
HVPGSFIFTYRRSSWNSFTITDGVFYEKWWTNSVQLPFAT*
>Urod_hit_Clanculus_margaritarius_1_mantle_c45374_g1_i1
QAGRYLPEYGKAKGDKAFFATCRDKELVSELTLQPIDRFALDGAIIFSDILVIPLALGLTALNDPGKGPVFADPIEKPED
VDRLNPNFDIHKELGYVYDAINLTRQKLEGRVPLFG
>Urod_hit_Clanculus_margaritarius_1_foot_c98280_g1_i1
TRQKLEGRVPLFGFSGAPWTLMKYMIENLGAGPSPNKARRFLVEYPEAGTKLLKILTGAVVRHLVEQVRAGAQILQVFDS
NGGELGPNLFTKYELPCLQEIAYKV
>Urod_hit_Calliostoma_zizyphinum_mantle_c26554_g1_i1
TRMAATQTHAQMPAQEFAPLQNDLVLRAARGEKTEKVPVWVMRQAGRYLPEYLKAKGDKAFFATCRDKELVSELTIQPID
RFSLDAAIIFSDILVIPLALGLKAQNDPGQGPVFADPIQKPEDLDRLNPDFNIIEELGYVYEAITLTRQKLKGRVPLFGF
SGAPWTLMKYMIENLGAGPNPNKTRRFLIEHPDAGEKLLQILTDAVVRHLVEQVRAGAQILQVFDSSCGELGPTLFTKFE
LPCLKEIAFRVKKTVKEEGLELIPMVVFAKDAYFATKQLAGTDYDVM
>Urod_hit_Calliostoma_zizyphinum_mantle_c79246_g1_i1
LALLTDAVVTHLVCQAVAGAQLLQVFESHAGVLTPALFNAFALPYLKQIVGQLRKELVERHGYTSSDLPPVIIFAKDGHF
AIDQLVNDAGYDVVSLDWTISLDQAQDVANNKVTLQGNLDPCALYAPND
>Urod_hit_Clanculus_pharaonius_mantle_c6567_g1_i1
PEYGQAKGDKAFFATCRDKELVSELTLQPIDRFPLDGAIIFSDILVIPLALGLTALNDPGKGPVFADPIEKPEDVDRLNP
SFDIHKELGYVYDAINLTRHKL
>Urod_hit_Clanculus_pharaonius_mantle_c81961_g1_i1
TGAVVCHLVEQVRAGAQILQVFDSNGGELGPNLFTKYELPCLQEIAYKVKMEVKQQELELVPMVVFAKDAHFATEQLAGI
GYDVVQIDWTQSPTHARRLTGSKVTLQGNLDPVNLFGTDEEIRHQVKEMVQKFGTQRYIATLGHGVMKDTNPDKLGVFID
AVHKYSR
