# Supplementary material for: Colorful seashells: Identification of haem pathway genes associated with the synthesis of porphyrin shell color in marine snails
Source: Ecol Evol. 2017 Oct 30;7(23):10379–97. doi: 10.1002/ece3.3552 (PMC5723588; doi:10.1002/ece3.3552)
Supplement: Supplementary file 4 [file ECE3-7-10379-s004.docx]

**Appendix 4. Mean Normalised values from qPCR for the first five genes in the haem pathway, calculated using 18S as a reference gene.**

| **Species** | **#** | **Tissue** | **ALAS (mean)** | **ALAS (S.E)** | **ALAD (mean)** | **ALAD (S.E)** | **PBGD (mean)** | **PBGD (S.E.)** | **UROS (mean)** | **UROS (S.E.)** | **UROD (mean)** | **UROD (S.E.)** |
| --- | --- | --- | --- | --- | --- | --- | --- | --- | --- | --- | --- | --- |
| *Clanculus margaritarius* | 1 | columellar muscle | 2.17E-06 | 1.59E-07 | 1.84E-06 | 1.22E-07 | 3.22E-05 | 2.85E-06 | 1.23E-05 | 7.23E-07 | 3.37E-05 | 3.72E-06 |
| *Clanculus margaritarius* | 2 | columellar muscle | 2.46E-06 | 2.33E-07 | 2.25E-06 | 2.64E-07 | 4.60E-05 | 4.68E-06 | 1.96E-05 | 2.06E-06 | 2.74E-05 | 3.95E-06 |
| *Clanculus margaritarius* | 1 | mantle | 2.31E-06 | 3.29E-08 | 2.55E-06 | 1.36E-07 | 5.16E-05 | 4.62E-06 | 1.28E-05 | 6.11E-07 | 3.01E-05 | 2.31E-06 |
| *Clanculus margaritarius* | 2 | mantle | 2.58E-06 | 7.35E-08 | 4.29E-06 | 1.03E-07 | 6.14E-05 | 2.20E-06 | 1.50E-05 | 1.30E-06 | 3.44E-05 | 5.06E-07 |
| *Clanculus margaritarius* | 1 | coloured foot | 5.22E-07 | 4.51E-08 | 5.41E-07 | 4.83E-08 | 2.55E-05 | 2.04E-06 | 1.69E-06 | 1.59E-07 | 6.45E-06 | 4.18E-07 |
| *Clanculus margaritarius* | 2 | coloured foot | 2.61E-07 | 3.04E-08 | 5.64E-07 | 6.03E-08 | 1.92E-05 | 2.42E-06 | 2.44E-06 | 2.81E-07 | 3.71E-06 | 4.04E-07 |
|  |  |  |  |  |  |  |  |  |  |  |  |  |
| *Clanculus pharaonius* | 1 | columellar muscle | 1.64E-06 | 4.88E-08 | 2.72E-06 | 1.60E-07 | 1.06E-04 | 2.50E-06 | 3.09E-05 | 1.01E-06 | 1.70E-05 | 1.12E-06 |
| *Clanculus pharaonius* | 2 | columellar muscle | 2.29E-06 | 7.62E-08 | 2.51E-06 | 1.18E-07 | 3.67E-05 | 2.31E-06 | 1.97E-05 | 6.82E-07 | 1.50E-05 | 1.34E-06 |
| *Clanculus pharaonius* | 3 | columellar muscle | 1.55E-06 | 5.72E-08 | 2.23E-06 | 8.81E-08 | 3.52E-05 | 1.00E-06 | 2.05E-05 | 1.09E-06 | 1.18E-05 | 1.11E-06 |
| *Clanculus pharaonius* | 4 | columellar muscle | 1.81E-06 | 1.02E-07 | 2.32E-06 | 1.48E-07 | 3.89E-05 | 1.78E-06 | 3.18E-05 | 2.54E-06 | 9.67E-06 | 3.85E-07 |
| *Clanculus pharaonius* | 5 | columellar muscle | 1.91E-06 | 7.42E-08 | 2.98E-06 | 1.87E-07 | 9.47E-05 | 9.60E-06 | 2.80E-05 | 2.06E-06 | 1.64E-05 | 8.39E-07 |
| *Clanculus pharaonius* | 6 | columellar muscle | 2.57E-06 | 2.54E-07 | 2.79E-06 | 4.09E-07 | 4.18E-05 | 4.06E-06 | 1.97E-05 | 1.65E-06 | 1.18E-05 | 1.21E-06 |
| *Clanculus pharaonius* | 1 | mantle | 3.22E-06 | 1.37E-07 | 3.22E-06 | 5.28E-07 | 7.87E-05 | 1.19E-05 | 1.42E-05 | 9.19E-07 | 1.73E-05 | 1.81E-06 |
| *Clanculus pharaonius* | 2 | mantle | 8.30E-06 | 5.74E-07 | 8.06E-06 | 6.06E-07 | 1.18E-04 | 6.30E-06 | 1.09E-05 | 4.49E-07 | 1.08E-05 | 1.24E-06 |
| *Clanculus pharaonius* | 3 | mantle | 4.77E-06 | 3.26E-07 | 6.48E-06 | 6.40E-07 | 1.54E-04 | 4.40E-06 | 1.58E-05 | 1.41E-06 | 2.15E-05 | 9.08E-07 |
| *Clanculus pharaonius* | 4 | mantle | 1.30E-05 | 8.68E-07 | 9.02E-06 | 5.71E-07 | 2.01E-04 | 1.87E-05 | 1.02E-05 | 8.47E-07 | 9.97E-06 | 7.30E-07 |
| *Clanculus pharaonius* | 5 | mantle | 2.82E-06 | 2.06E-07 | 2.81E-06 | 2.79E-07 | 4.22E-05 | 3.50E-06 | 1.17E-05 | 1.65E-06 | 1.55E-05 | 1.13E-06 |
| *Clanculus pharaonius* | 6 | mantle | 4.18E-06 | 3.64E-07 | 2.78E-06 | 2.94E-07 | 4.48E-05 | 4.38E-06 | 1.22E-05 | 2.26E-06 | 1.54E-05 | 1.14E-06 |
| *Clanculus pharaonius* | 1 | coloured foot | 5.62E-06 | 5.99E-07 | 3.63E-06 | 3.04E-07 | 1.27E-04 | 1.02E-05 | 1.61E-05 | 1.93E-06 | 7.42E-06 | 9.69E-07 |
| *Clanculus pharaonius* | 2 | coloured foot | 3.24E-06 | 4.58E-07 | 8.63E-07 | 1.43E-07 | 3.36E-05 | 4.63E-06 | 6.98E-06 | 1.03E-06 | 2.00E-06 | 3.42E-07 |
| *Clanculus pharaonius* | 3 | coloured foot | 2.01E-06 | 1.81E-07 | 1.06E-06 | 7.84E-08 | 2.80E-05 | 2.04E-06 | 2.68E-06 | 2.21E-07 | 2.01E-06 | 1.50E-07 |
| *Clanculus pharaonius* | 4 | coloured foot | 4.18E-06 | 7.26E-07 | 1.97E-06 | 3.45E-07 | 6.17E-05 | 9.89E-06 | 8.50E-06 | 1.53E-06 | 1.27E-06 | 2.21E-07 |
| *Clanculus pharaonius* | 5 | coloured foot | 4.44E-06 | 2.05E-07 | 2.44E-06 | 3.36E-07 | 5.26E-05 | 5.90E-06 | 1.43E-05 | 8.10E-07 | 4.40E-06 | 2.33E-07 |
| *Clanculus pharaonius* | 6 | coloured foot | 3.16E-06 | 4.94E-07 | 1.32E-06 | 2.54E-07 | 4.25E-05 | 5.88E-06 | 1.00E-05 | 1.39E-06 | 2.30E-06 | 4.70E-07 |
|  |  |  |  |  |  |  |  |  |  |  |  |  |
| *Calliostoma zizyphinum* | 1 | columellar muscle | 7.29E-07 | 1.10E-07 | 8.58E-08 | 2.01E-08 | 1.81E-03 | 2.32E-04 | 1.86E-07 | 5.84E-09 | 1.57E-03 | 6.55E-05 |
| *Calliostoma zizyphinum* | 2 | columellar muscle | 2.47E-06 | 1.11E-06 | 3.34E-07 | 1.26E-07 | 9.92E-03 | 6.58E-04 | 5.83E-08 | 1.86E-08 | 8.59E-03 | 1.59E-03 |
| *Calliostoma zizyphinum* | 3 | columellar muscle | 2.64E-06 | 7.89E-07 | 9.47E-08 | 3.17E-08 | 4.25E-03 | 3.33E-04 | 6.07E-08 | 9.29E-09 | 1.78E-03 | 1.46E-04 |
| *Calliostoma zizyphinum* | 4 | columellar muscle | 6.90E-07 | 1.88E-07 | 1.74E-08 | 1.92E-08 | 2.15E-03 | 4.40E-04 | 1.75E-07 | 2.77E-08 | 1.20E-03 | 7.38E-05 |
| *Calliostoma zizyphinum* | 1 | mantle | 1.66E-05 | 9.44E-07 | 9.24E-07 | 1.08E-08 | 2.73E-03 | 2.70E-04 | 1.89E-08 | 1.37E-09 | 6.24E-03 | 1.20E-04 |
| *Calliostoma zizyphinum* | 2 | mantle | 1.70E-05 | 1.19E-06 | 7.93E-07 | 6.43E-08 | 5.21E-03 | 6.34E-04 | 2.11E-08 | 1.85E-09 | 5.70E-03 | 4.67E-04 |
| *Calliostoma zizyphinum* | 3 | mantle | 2.14E-05 | 2.79E-06 | 4.68E-07 | 7.54E-08 | 3.07E-03 | 1.52E-04 | 3.28E-08 | 3.62E-09 | 3.10E-03 | 1.92E-04 |
| *Calliostoma zizyphinum* | 4 | mantle | 9.69E-05 | 9.33E-06 | 5.49E-07 | 5.99E-08 | 3.39E-03 | 3.07E-04 | 4.25E-08 | 4.94E-09 | 2.98E-03 | 2.93E-04 |
